# Molecular Origins of Functional Diversity in Benzylisoquinoline Alkaloid Methyltransferases

**DOI:** 10.3389/fpls.2019.01058

**Published:** 2019-08-30

**Authors:** Jeremy S. Morris, Peter J. Facchini

**Affiliations:** Department of Biological Sciences, University of Calgary, Calgary, AB, Canada

**Keywords:** benzylisoquinoline, alkaloid, methyltransferase, specialized metabolism, molecular evolution

## Abstract

*O*- and *N*-methylations are ubiquitous and recurring features in the biosynthesis of many specialized metabolites. Accordingly, the methyltransferase (MT) enzymes catalyzing these modifications are directly responsible for a substantial fraction of the vast chemodiversity observed in plants. Enabled by DNA sequencing and synthesizing technologies, recent studies have revealed and experimentally validated the trajectories of molecular evolution through which MTs, such as those biosynthesizing caffeine, emerge and shape plant chemistry. Despite these advances, the evolutionary origins of many other alkaloid MTs are still unclear. Focusing on benzylisoquinoline alkaloid (BIA)-producing plants such as opium poppy, we review the functional breadth of BIA *N*- and *O*-MT enzymes and their relationship with the chemical diversity of their host species. Drawing on recent structural studies, we discuss newfound insight regarding the molecular determinants of BIA MT function and highlight key hypotheses to be tested. We explore what is known and suspected concerning the evolutionary histories of BIA MTs and show that substantial advances in this domain are within reach. This new knowledge is expected to greatly enhance our conceptual understanding of the evolutionary origins of specialized metabolism.

## Introduction

The incredible diversity of plant metabolism has been a topic of fascination for centuries, and yet our appreciation for its scope continues to grow. A recent study examined more than a hundred thousand metabolite–plant species relationships and concluded that each species contains, on average, 4.7 unique metabolites, which sum to an estimate of more than 1 million distinct metabolites across the kingdom (Afendi et al., 2012). An earlier analysis of a smaller database calculated the existence of only 1.6 unique metabolites per plant species, suggesting that our estimates of the breadth of plant metabolism will continue to grow as we collect more data (Shinbo et al., 2006). These numbers are in line with previous estimates ranging from 200,000 to 1,000,000 plant metabolites in total (Saito and Matsuda, 2009).

Alkaloids, broadly defined as low-molecular weight heterocyclic nitrogenous compounds, are thought to occur in roughly 20% of plant species (Ziegler and Facchini, 2008). At least 12,000 unique molecules of this class are known, which can be classified as either protoalkaloids (e.g., mescaline, ephedrine), where the nitrogen is not cyclic, pseudoalkaloids (e.g., steroidal and diterpene alkaloids, caffeine), where the primary biosynthetic origin is not an amino acid, and “true” alkaloids where most of the molecule, including the heterocyclic nitrogen, is derived from an amino acid precursor (Hegnauer, 1988; Waterman, 1998). This latter group is most diverse and includes biosynthetic end products derived from phenylalanine, tyrosine, tryptophan, ornithine, arginine, lysine, histidine, and anthranillic acid.

Although the foundation of plant alkaloid chemical diversity begins with the combination and rearrangement of the aforementioned building blocks, each basic carbon skeleton can give rise to a great number of “decorated” variants with various functional group substitutions that alter the molecule’s biochemical characteristics. For example, *N*-methylation of xanthine/xanthosine during biosynthesis of caffeine allows for the production of up to seven differentially methylated products (Huang et al., 2016). Similarly, the potential for two *N*-methyl and four *O*-methyl groups on simple benzylisoquinoline alkaloids (BIAs) such as norlaudanosoline makes up to 30 distinct molecules possible. The addition of methyl groups to an alkaloid molecule can have important consequences regarding its chemical properties and thus shift biological activity. Methylation can invert the polarity of an electronegative moiety, shift the molecule’s stereoelectronic profile, increase overall hydrophobicity, increase steric bulk, and promote or prevent certain conformations of the molecule (Wessjohann et al., 2014). In the extreme case, methylation of a tertiary amine results in a quaternary ammonium cation, which is substantially more hydrophilic and lipophobic. For example, *O*-methylation of the monoterpene indole alkaloid noribogaine results in a much less polar compound (ibogaine), which is readily sequestered to lipophilic compartments of the mammalian brain (Zubaran, 2000). The *O*-methylated molecule displays differential binding to neurotransmitter receptors versus the parent compound, resulting in substantially more toxicity as measured by the dose required to induce tremors and cerebellar damage. Similarly, the *O*-methylated BIA thebaine is much more of a stimulant and much less of an effective painkiller than morphine, which is fully *O*-demethylated (Navarro and Elliott, 1971).

### Plant Alkaloid *O*-Methyltransferases

**Figure 1:**
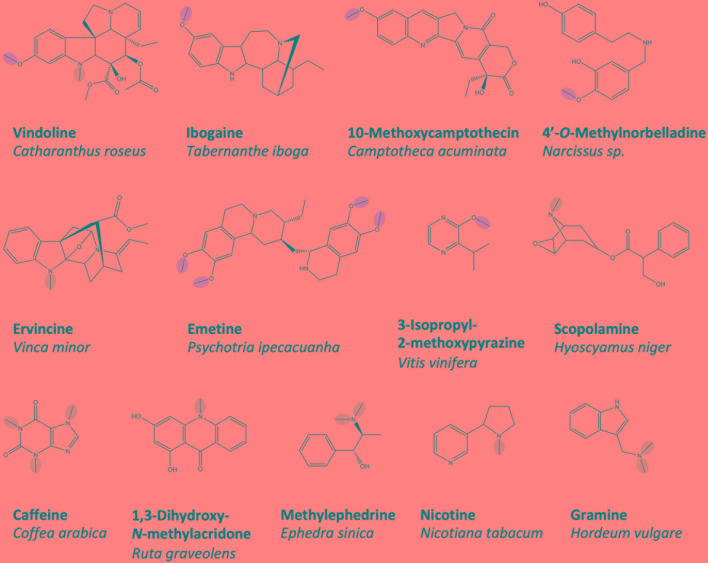
A selection of plant alkaloids biosynthesized by *O*- and *N*-methyltransferases, which have been cloned and functionally characterized *in vitro*. *O*- and *N*-methyl groups reportedly installed by the enzymes are circled in *red* and *green*, respectively. Representative benzyliosqinoline alkaloids produced are shown in **Figure 2**.

Underlying the massive number of differentially methylated plant alkaloids is a large and heterogeneous group of methyltransferase (MT) enzymes thought to be specialized for various substrates (**Figure 1**). Several *O*-methyltransferase (OMT) enzymes, which participate in the terminal steps of monoterpene indole alkaloid biosynthesis, have been identified and cloned, including an OMT leading to the production of vindoline in *Catharanthus roseus* (Levac et al., 2008), an OMT producing ibogaine in *Tabernanthe iboga* (Farrow et al., 2018) and a 10-hydroxycamptothecin OMT from *Camptotheca acuminata* (Salim et al., 2018). Three OMTs contributing to the biosynthesis of monoterpene isoquinoline alkaloids such as emetine have been cloned from *Psychotria ipecacuanha* (Nomura and Kutchan, 2010). Studies on the biosynthesis of Amaryllidaceae alkaloids such as galanthamine in *Narcissus* spp. and *Lycoris aurea* allowed for the isolation of two norbelladine 4′OMT enzymes (Kilgore et al., 2014; Sun et al., 2018). Although not traditionally included in most lists of alkaloids due to an unclear biosynthetic origin, the volatile heterocyclic nitrogenous methoxypyrazines, which contribute to the flavor profile of grapes, also require *O*-methylation in their biosynthesis. To date, four *Vitis vinifera* OMTs implicated in this pathway have been cloned (Dunlevy et al., 2010, Dunlevy et al., 2013; Guillaumie et al., 2013). Quite a few OMTs implicated in benzylisoquinoline alkaloid biosynthesis have been cloned, and these will be reviewed in a dedicated section below.

### Plant Alkaloid *N*-Methyltransferases

Particularly well studied are *N*-methyltransferases (NMTs), which catalyze the terminal biosynthetic steps producing xanthine alkaloids (e.g. caffeine) in various plants. Cloned representatives include caffeine synthase, theobromine synthase and 7-methylxanthosine synthase from *Coffea arabica* (Mizuno et al., 2003a; Mizuno et al., 2003b) as well as homologs in *Camellia*, *Theobroma*, *Paullinia*, and *Citrus* (Kato et al., 2000; Yoneyama et al., 2006; Schimpl et al., 2014; Huang et al., 2016). Cloned NMTs contributing to the biosynthesis of monoterpene indole alkaloids include one from *C. roseus* leading to the production of vindoline (Liscombe et al., 2010) and two related picrinine NMTs from *Apocynaceae* species (Levac et al., 2016). NMTs from less intensively studied pseudoalkaloid pathways have also been cloned, such as those implicated in the biosynthesis of gramine (Larsson et al., 2006) and ephedrine in *Hordeum vulgare* and *Ephedra sinica*, respectively (Morris et al., 2018). The large number of BIA NMTs that have been characterized at the molecular level will be discussed further in the body of this work.

Although most known alkaloid MTs contribute to the final stages of biosynthesis, this is not a firm rule. Putrescine NMT (PMT), which synthesizes *N*-methylputrescine, catalyzes the first step in pathways leading to several alkaloid classes including the pyridines (e.g., nicotine), tropane alkaloids (e.g., scopolamine), or calystegines in various plant species (Biastoff et al., 2009). A larger number of PMTs have been cloned, including those from *Nicotiana tabacum*, *Solanum tuberosum*, and various other Solanaceae and Convolvulaceae species (Hibi, 1994; Stenzel et al., 2006; Teuber et al., 2007; Junker et al., 2013). Similarly, an anthranilate NMT diverts metabolic flux away from tryptophan biosynthesis into the acridone alkaloid biosynthetic pathway and has been cloned from *Ruta graveolens* (Rohde et al., 2007).

Despite the ever-growing list of characterized and cloned MTs, there remain many biosynthetic pathways to explore. For example, *O*- and *N*-methylation of phenethylamine alkaloids in many lineages including the Cactaceae (e.g., mescaline), tryptamine alkaloids in *Acacia*, *Citrus*, *Phalaris*, and others (e.g., *N*,*N*-dimethyltryptamine), quinolizidine alkaloids in Fabaceae (e.g., *N*-methylcytisine), and the diverse Amaryllidaceae alkaloids remains understudied (Smith, 1977a; Smith, 1977b; Wink, 1984; Jin and Xu, 2013).

### Caffeine Biosynthesis as a Model of Research Potential

The wealth of cloned alkaloid MTs has proven to be a fertile area in which to examine the relationships between enzyme function and plant biochemistry. Aside from the characterization of natural variants and concomitant identification of sequence–function correlations, modern structural biology and DNA manipulation methods have allowed experimental approaches to directly probe the features controlling enzyme properties. In select cases, the molecular evolutionary trajectories, which resulted in extant enzyme, and their specific properties have also been elucidated, providing some insight into the origins of specialized biochemical pathways and the exceptional chemodiversity of plants.

Perhaps the best example of the research sequence described above relates to the biosynthesis of caffeine and other xanthine alkaloids. Building on a long history of research using radiolabeled tracers to elucidate the pathway, workers eventually showed unequivocally that caffeine is synthesized from xanthosine *via* a series of *N*-methylation reactions (Suzuki and Takahashi, 1975; Suzuki and Takahashi, 1976; Ashihara et al., 1996; Kato et al., 1996; Mösli Waldhauser et al., 1997a; Mösli Waldhauser et al., 1997b; Kato et al., 1999). Shortly thereafter, cDNAs encoding these enzymes were cloned from *Camellia sinensis* (Kato et al., 2000) and *Coffea arabica* (Ogawa et al., 2001; Uefuji, 2003; Mizuno et al., 2003a, Mizuno et al., 2003b) and later from *Paullinia cupana* (Schimpl et al., 2014), *Theobroma cacao* (Yoneyama et al., 2006), and *Citrus sinensis* (Huang et al., 2016). Examination of their coding sequences showed that these xanthine NMTs belong to the SABATH (salicylic acid, benzoic acid, theobromine) family of methyltransferases, which typically methylate oxygen atoms, suggesting a relatively recent change of function in xanthine alkaloid-producing species. Intriguingly, greater sequence similarity between the functionally distinct MTs within one species (e.g., 80% identity between CaXMT, CaMXMT, and CaDXMT from *C. arabica*) compared to those of analogous function in other plants (e.g., less than 40% identity between CaDXMT and TCS1 from *C. sinsensis*) lead to the hypothesis that xanthine MTs in different lineages have parallel and convergent evolutionary histories.

With coding sequences available to produce heterologous proteins, the molecular determinants of function were studied (Yoneyama et al., 2006; McCarthy and McCarthy, 2007). Active-site features facilitating binding of xanthine molecules, in general (e.g., hydrophobic pocket), and individual residues implicated in substrate specificity (e.g., Ser316 and Tyr356 in CaXMT hydrogen bonding with xanthine but not methylxanthine) were proposed based on comparative analysis of crystal structures in complex with various substrates. Next, site-directed mutagenesis allowed for experimental validation of these hypotheses (Yoneyama et al., 2006; Huang et al., 2016; Jin et al., 2016).

More recently, whole genome sequencing of *Coffea*, *Camellia*, and *Theobroma* species began to unveil the genetic mechanisms leading to the evolution of caffeine biosynthesis (Argout et al., 2011; Denoeud et al., 2014; Xia et al., 2017; Wei et al., 2018). Taken together, the studies strongly suggested the occurrence of multiple independent and convergent evolutionary trajectories, in which gene duplication and functional divergence lead to caffeine biosynthesis. However, these results did not yet explain how or why this biochemical feature arose so readily in distantly related plants.

Clarification of one evolutionary trajectory leading to caffeine biosynthesis in *Citrus* was recently provided by a paleomolecular biology method known as ancestral enzyme reconstruction (Thornton, 2004; Huang et al., 2016). Barkmann and colleagues showed that an ancestral SABATH enzyme was likely exapted to catalyze *N*-methylation of caffeine pathway intermediates and that, following its duplication, a single amino acid substitution in each of the descendant enzymes was sufficient to create a fully functional caffeine biosynthetic pathway. Their results concerning the unusually short mutational distance between SABATH OMT and xanthine NMT functions showed how evolutionary innovation was able to repeatedly converge on caffeine biosynthesis in multiple plant orders.

The extent of our knowledge surrounding the evolution of caffeine biosynthesis showcases what can be achieved with modern research tools and paradigms. In the following section, we outline the current state of knowledge regarding *O*- and *N*-methyltransferases involved in BIA biosynthesis and their contribution to host plant chemodiversity. Drawing on structural and functional studies, we outline what is known regarding the molecular determinants of their differing activities and explore what is suspected regarding their evolution. We show that the field is ripe for substantial advances paralleling those obtained with respect to caffeine biosynthesis and suggest key hypotheses and experiments by which they may be tested.

## Contribution of Methyltransferases to Benzylisoquinoline Alkaloid Biosynthesis

BIAs have been studied for centuries (Hagel and Facchini, 2013 and references therein), and much of their biosynthesis has been revealed, albeit only in a handful of model systems, which can only approximate the biosynthetic diversity in the thousand or more BIA-producing plant species (Shulgin and Perry, 2002). These discoveries and their historical context have been extensively reviewed elsewhere and will only be summarized here in order to highlight the involvement of methyltransferases. A tremendous number of BIA MTs have been characterized in plant extracts, and although these have contributed greatly to our understanding of BIA biosynthesis, we will focus herein on those that have been cloned and studied at the molecular level. The nine Ranunculales species from which BIA MTs have been characterized at this level are *Papaver somniferum*, *P. bracteatum*, *Glaucium flavum*, *Thalictrum flavum*, *Coptis japonica*, *C. chinensis*, *C. teeta*, *Dactylicapnos scandens*, and *Eschscholzia californica*. Literature reports concerning the isolation and characterization of each of the MTs discussed are cited in **Supplementary Table 1**.

**Figure 2:**
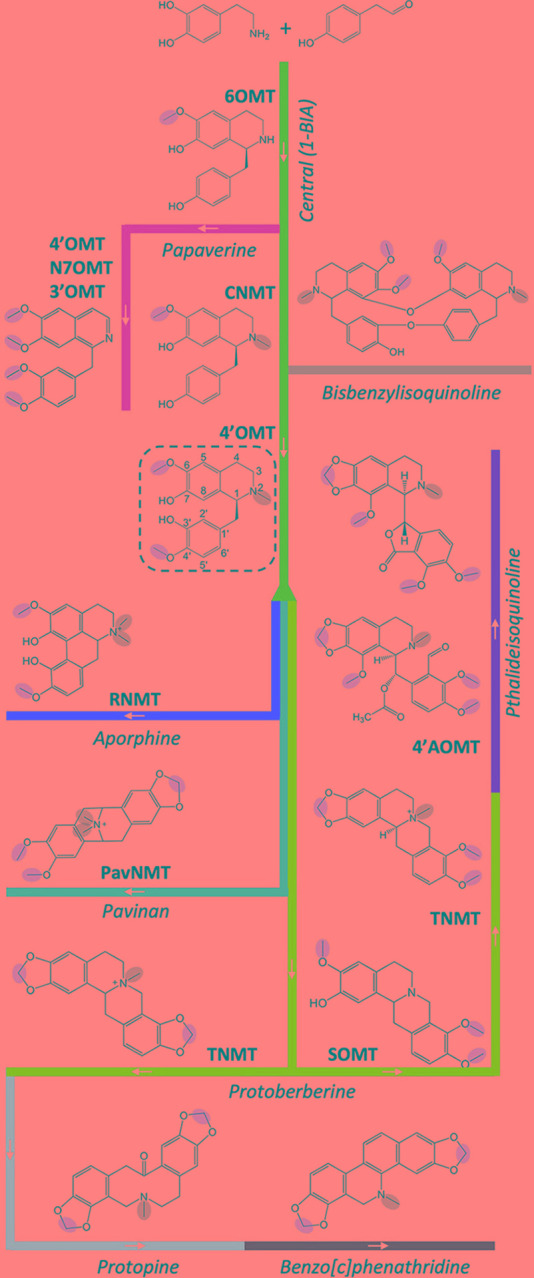
Contributions of *O*- and *N*-methyltransferases to BIA biosynthesis. A central pathway (*dark blue*) leads to the core 1-BIA intermediate (*S*)-reticuline (dashed rectangle) from which various branch pathways diverge, including those leading to aporphines (*red*), pavinans (*purple*), protoberberines (*light blue*), protopines (*pink*), benzo[c]phenanthridines (*green*), and pthalideisoquinolines (*brown*). The bisbenzylisoquinolines (*gray*) are typically produced by dimerization of various 1-BIA intermediates. The branch pathway to papaverine (*yellow*) is unusual in diverging from the central pathway prior to *N*-methylation. The 6OMT (norcoclaurine 6-*O*-methyltransferase), 4′-OMT (3′-hydroxy-*N*-methylcoclaurine 4′-*O*-methyltransferase), N7OMT (norreticuline 7-*O*-methyltransferase), 3′OMT (3′-*O*-methyltransferase), SOMT (scoulerine *O*-methyltransferase), 4′-AOMT (4′‐*O*‐desmethyl‐3‐*O*‐acetylpapaveroxine 4′-*O*-methyltransferase), CNMT (coclaurine *N*-methyltransferase), RNMT (reticuline *N*-methyltransferase), PavNMT (pavine *N*-methyltransferase), and TNMT (tetrahydroprotoberberine *N*-methyltransferase) are drawn on branches representing their major or physiologically relevant activities. *O*- and *N*-methyl groups reportedly installed by the enzymes are circled in *red* and *green*, respectively. A detailed BIA biosynthetic pathway is available in Hagel and Facchini (2013).

In *P. somniferum*, the most studied BIA model organism, a central pathway for BIA biosynthesis begins with a condensation of two tyrosine derivatives (dopamine and 4-hydroxyphenylacetaldehyde) to form (*S*)-norcoclaurine (**Figure 2**). Acting on this base skeleton, norcoclaurine-6-*O*-methyltransferase (6OMT) transfers a methyl group onto one of the isoquinoline moiety hydroxyl groups to yield (*S*)-coclaurine. Given the core role of this enzymes, it is unsurprising that nine cognate cDNAs from six species have been isolated and shown to encode this activity (**Table 1**; **Supplementary Table 1**) (Morishige et al., 2000; Ounaroon et al., 2003; Facchini and Park, 2003; Samanani, 2005; Tamura et al., 2006; Desgagné-Penix and Facchini, 2012; Chang et al., 2015; Robin et al., 2016; He et al., 2018). Interestingly, several species (e.g., *G. flavum*, *C. chinensis*) seem to express multiple distinct transcripts encoding enzymes with this activity despite sharing only 45–55% amino acid identity (**Supplementary Figure 1**). Downstream in the central pathway, a first *N*-methyl group is installed by coclaurine *N*-methyltransferase (CNMT), which has been cloned from four BIA-producing species (Choi et al., 2002; Facchini and Park, 2003; Samanani, 2005; Minami et al., 2008; Liscombe et al., 2009; Desgagné-Penix and Facchini, 2012; Hagel et al., 2015). Notably, four transcripts encoding enzymes with CNMT activity (59–66% AA identity; **Supplementary Figure 2**) have been cloned from *G. flavum*; however, their individual contributions to biosynthesis in the host plant remain to be assessed. The final step in the central pathway is catalyzed by 3′-hydroxy-*N*-methylcoclaurine 4′-*O*-methyltransferase (4′OMT) and yields the triple-methylated central intermediate (*S*)-reticuline. Corresponding transcripts have been cloned from three species (Morishige et al., 2000; Facchini and Park, 2003; Ounaroon et al., 2003; Ziegler et al., 2005; Desgagné-Penix and Facchini, 2012; Chang et al., 2015). Widespread occurrence of the above three MTs in BIA-producing species is consistent with the current model in which all end product alkaloids derive from reticuline or, less commonly, from upstream central pathway intermediates (Hagel et al., 2015).

**Table 1:** *O*- and *N*-methyltransferases reportedly able to catalyze the various major BIA biosynthetic activities *in vitro*. Details of the activities and corresponding citations are provided in Supplementary Table 1.

6OMTs	4′OMTs	N7OMT	7OMTs	3′OMTs	SOMTs	4′AOMTs	CNMTs	RNMTs	TNMTs
GflOMT1	GflOMT1	PsN7OMT	GflOMT1	PsSOMT1	GflOMT1	PsSOMT2: SOMT3	GflNMT1	PsRNMT	GflNMT2
GflOMT2	GflOMT2		GflOMT6	GflOMT1	GflOMT2	PsSOMT2: 6OMT	GflNMT4	GflNMT4	GflNMT3
Ps6OMT	Ps4’OMT2		PsSOMT1	GflOMT2	GflOMT6		GflNMT5	GflNMT5	PsTNMT
Ps7OMT	Ps7OMT		Ps7OMT	GflOMT6	GflOMT7		GflNMT6	TfPavNMT	EcTNMT
Cj6OMT	Cj4’OMT		Cc6OMT1	Ec7OMT	PsSOMT1		PsCNMT	EsPaNMT*	PbTNMT
Cc6OMT1			Cc6OMT2	EcS/ROMT	PsSOMT2		CjCNMT		TfPavNMT
Cc6OMT2			Ct6/7OMT		PsSOMT3		TfCNMT		TfCNMT
Ct6/7OMT			Ec7OMT		CjSOMT		EsPaNMT*		EsPaNMT*
Tf6OMT			EcS/ROMT		CjCoOMT				
					CtSOMT				
					Ec7OMT				
					EcS/ROMT				

6OMT, 6-O-methyltransferase 4′OMT, 4′-O-methyltransferase; N7OMT, norreticuline 7-O-methyltransferase; 7OMT, 7-O-methyltransferase; 3′OMT, 3′-O-methyltransferase; SOMT, scoulerine-O-methyltransferase; 4′AOMT, 4′‐O‐desmethyl‐3‐O‐acetylpapaveroxine 4′-O-methyltransferase; CNMT, coclaurine N-methyltransferase; RNMT, reticuline N-methyltransferase; TNMT, tetrahydroprotoberberine N-methyltransferase. Ephedra sinica phenylalkylamine N-methyltransferase (EsPaNMT), denoted with an asterisk, has promiscuous activity with various BIAs but does not participate in their biosynthesis in planta. Dactylicapnos scandens cortyuberine 7-O-methyltransferase (DsC7OMT) and Eschscholzia californica 10-hydroxysanguinarine O-methyltransferase (EcG11OMT) have unique substrate ranges and are not included in the table.

The 4′-*O*-methylation also contributes to papaverine biosynthesis *via* a branch that diverges from the central pathway prior to the action of CNMT and, instead, passes through (*S*)-norreticuline. Next, the exceptionally substrate-specific norreticline 7-*O*-methyltransferase (N7OMT) installs a third *O*-methyl to yield (*S*)-norlaudanine. The single N7OMT cloned to date occurs in *P. somniferum*, which is consistent with the somewhat-restricted taxonomic distribution of papaverine (Shulgin and Perry, 2002; Pienkny et al., 2009; Desgagné-Penix and Facchini, 2012). Although the preponderance of evidence at this time supports an *N*-desmethylated biosynthetic scheme for papaverine, it was also proposed that biosynthesis might pass through (*S*)-reticuline. Indeed, a reticuline 7-*O*-methyltransferase (7OMT) has also been cloned from *P. somniferum*, along with eight additional transcripts encoding 7OMT-like enzymes from five species (Ounaroon et al., 2003; Fujii et al., 2007; Dang and Facchini, 2012; Desgagné-Penix and Facchini, 2012; Chang et al., 2015; Purwanto et al., 2017; He et al., 2018). The 3′-*O*-methylation, which completes the series of four methylations required to yield papaverine, may be catalyzed in part *via P. somniferum* SOMT1 despite the fact that its *in vitro* activity substantially favors other substrates (Dang and Facchini, 2012). An additional five transcripts encoding enzymes with comparable 3′OMT activities have been cloned from three species (Fujii et al., 2007; Chang et al., 2015; Purwanto et al., 2017). Aside from papapverine, many 3′- and 7-*O*-methylated simple BIAs (e.g., laudanine, laudanosine) and potential derivatives (e.g., jatorrhizine, tetrahydropalmatine) are known; however, the involvement of any given OMT in their biosynthesis remains speculative due to a lack of *in planta* experimental evidence.

Starting from (*S*)-reticuline, a short branch yields the taxonomically widespread aporphine alkaloid (*S*)-magnoflorine (e.g., Ranunculales, Laurales, Magnoliales, Sapindales, Piperales, *etc*.) (Shulgin and Perry, 2002). Acting on either reticuline or its aporphine derivative (corytuberine), *P. somniferum* reticuline *N*-methyltransferase (RNMT, named as such to reflect *in vitro* substrate preference; **Table 1**; **Supplementary Table 1**) installs a second methyl group resulting in a quaternary nitrogen atom. To date, three additional transcripts encoding RNMT-like enzymes have been reported in two other BIA-producing plant species, both of which are also known to accumulate (*S*)-magnoflorine and related quaternary alkaloids (Shulgin and Perry, 2002; Liscombe et al., 2009; Hagel et al., 2015; Torres et al., 2016). Of particular note is the enzyme cloned from *T. flavum* and named pavine *N*-methyltransferase (TfPavNMT). Whereas the enzyme efficiently catalyzes the signature RNMT-like activity and might contribute to quaternary aporphine biosynthesis, it also uniquely accepts pavinan alkaloids and is thought to participate in the biosynthesis of *N*-methylescholzidine, an uncommon BIA, which accumulates in *Thalictrum* spp (Liscombe et al., 2009). In *D. scandens*, a corytuberine 7-*O*-methyltransferase (DsC7OMT) was identified, and preliminary analysis linked it to the biosynthesis of isocorydine (He et al., 2017).

Several longer branch pathways employing MT reactions also diverge from (*S*)-reticuline to produce protoberberines (e.g., stylopine), protopines (e.g., protopine), benzo[c]phenanthridines (e.g., sanguinarine), and pthatlideisoquinolines (e.g., noscapine) (**Figure 2**). Acting on the first protoberberine intermediate, scoulerine 9-*O*-methyltransferase (SOMT) installs a third *O*-methyl group to yield tetrahydrocolumbamine. In agreement with the relatively common occurrence of protoberberines in BIA-producing plants, known enzymes with SOMT activity are encoded by 12 transcripts isolated from five different species (Takeshita et al., 1995; Morishige et al., 2000; Fujii et al., 2007; Dang and Facchini, 2012; Chang et al., 2015; Purwanto et al., 2017; He at al., 2018). A functionally similar enzyme, which preferentially targets the 2-hydroxyl of quaternary protoberberine columbamine, was cloned only from *C. japonica* (CjCoOMT) (Morishige et al., 2002). En route to noscapine biosynthesis, tetrahydroprotoberberine NMT (TNMT) transfers a methyl group onto a bicyclic nitrogen atom, yielding the quaternary product *N*-methylcanadine. In addition to the canonical representative isolated from *P. somniferum*, six other TNMT-like enzymes have been cloned from four other species (Samanani, 2005; Liscombe and Facchini, 2007; Liscombe et al., 2009; Hagel et al., 2015; Torres et al., 2016). Intriguingly, the final *O*-methylation required to produce noscapine was recently shown to involve a heterodimer composed of PsSOMT2 and either PsSOMT3 or Ps6OMT (Li and Smolke, 2016; Park et al., 2018). No equivalent enzymes are presently known in other species, which is consistent with the lack of reports of noscapine in BIA producing species outside Papaveraceae (Shulgin and Perry, 2002). Aside from the role outlined above, TNMTs also participate in a separate branch pathway leading first to protopines and then to benzo[c]phenanthridines *via* synthesis of (*S*)-*cis*-*N*-methylstylopine. This product accumulates to a substantial degree in *T. flavum*, which is inconsistent with the relatively modest stylopine NMT activity reported for TfPavNMT (Liscombe et al., 2009). Thus, it seems likely that one of the additional NMT transcripts recently identified in that species’ transcriptome encodes an enzyme more dedicated to protoberberine substrates (Hagel et al., 2015). Acting further downstream in the benzo[c]phenanthridine branch pathway, a functionally unique OMT from *E. californica* (EcG11OMT) apparently targets 10-hydroxysanguinarine (Purwanto, 2017).

Aside from the above, a number of transcripts have been cloned and found to encode enzymes with high homology to BIA MTs but with no discernible activity *in vitro* (Liscombe and Facchini, 2007; Chang et al., 2015; Morris and Facchini, 2016; Purwanto et al., 2017; He et al., 2018). Although these may, in fact, be inactive with respect to BIAs, it remains possible that they have not been assayed under appropriate conditions or with proper substrates. These mysterious transcripts include at least one NMT each from *P. somniferum* and *Arabidopsis*, as well as a large number of OMTs from *G. flavum*, *P. somniferum*, *E. californica*, and *Coptis* spp. In the latter group, substantial homology to other types of plant OMTs makes identification of those targeting BIAs quite challenging.

As revealed in **Table 1**, the majority of BIA MTs are known to catalyze many additional reactions beyond those prototypical conversions represented by their names and position on orderly, linear biosynthetic pathways as traditionally drawn (**Figure 2**). Nevertheless, the targeted nature of *in vitro* biochemical characterization means that all reports necessarily underestimate the catalytic range of BIA MTs. The lack of specificity reported for BIA MTs occurs at two levels: substrate promiscuity, wherein the enzyme can methylate a number of different molecules, and product promiscuity, wherein a single substrate is methylated one or more times at various positions to yield different products (O’Brien and Herschlag, 1999; Hult and Berglund, 2007). On the other hand, BIA MTs do not show catalytic promiscuity, which is the ability to carry out distinct types of chemical transformations. Thus far, BIA OMTs and NMT have only been shown to catalyze *O*- and *N*-methylation, respectively, unlike certain SABATH MTs that target both *O* and *N* atoms (Huang et al., 2016). As shown for PsRNMT with respect to magnoflorine biosynthesis, the most substantial activity of an enzyme *in vitro* may not correlate with its function *in planta* (Morris and Facchini, 2016). This widespread promiscuity has led to an appreciation for the existence of multidimensional “metabolic grids”, which diversify the potential routes by which a plant may make any given end product. Experimental evidence for major, minor, or even “silent” routes in BIA biosynthesis has been given by gene knockdown experiments in whole plants and cell cultures (Fujii et al., 2007; Desgagné-Penix and Facchini, 2012).

## Molecular and Structural Determinants of Function

### *O*-Methyltransferases

To date, structures have been reported for *T. flavum* norcoclaurine 6OMT (Tf6OMT; PDB 5ICE) and *P. somniferum* scoulerine *O*-methyltransferase 1 (PsSOMT1; PDB 6I6K) (Robin et al., 2016; Cabry et al., 2019). While these structures have allowed for the generation of compelling hypotheses concerning substrate binding and catalysis, relatively little experimental work (e.g., site-directed mutagenesis) is presently available in support of their validity. The overall structures, each composed of an N-terminal dimerization domain linked to a C-terminal substrate binding domain, are consistent with those previously reported for SAM-dependent OMTs in plants such as *Medicago sativa* Caffeic acid OMT (MsCOMT; PDB 1KYZ), *M. trunculata* isoflavonoid OMT (MsIOMT; PDB 1FP2), and *M. sativa* chalcone OMT (MsChOMT; PDB 1FP1) (Zubieta et al., 2001; Zubieta, 2002).

#### Dimerization

**Figure 3:**
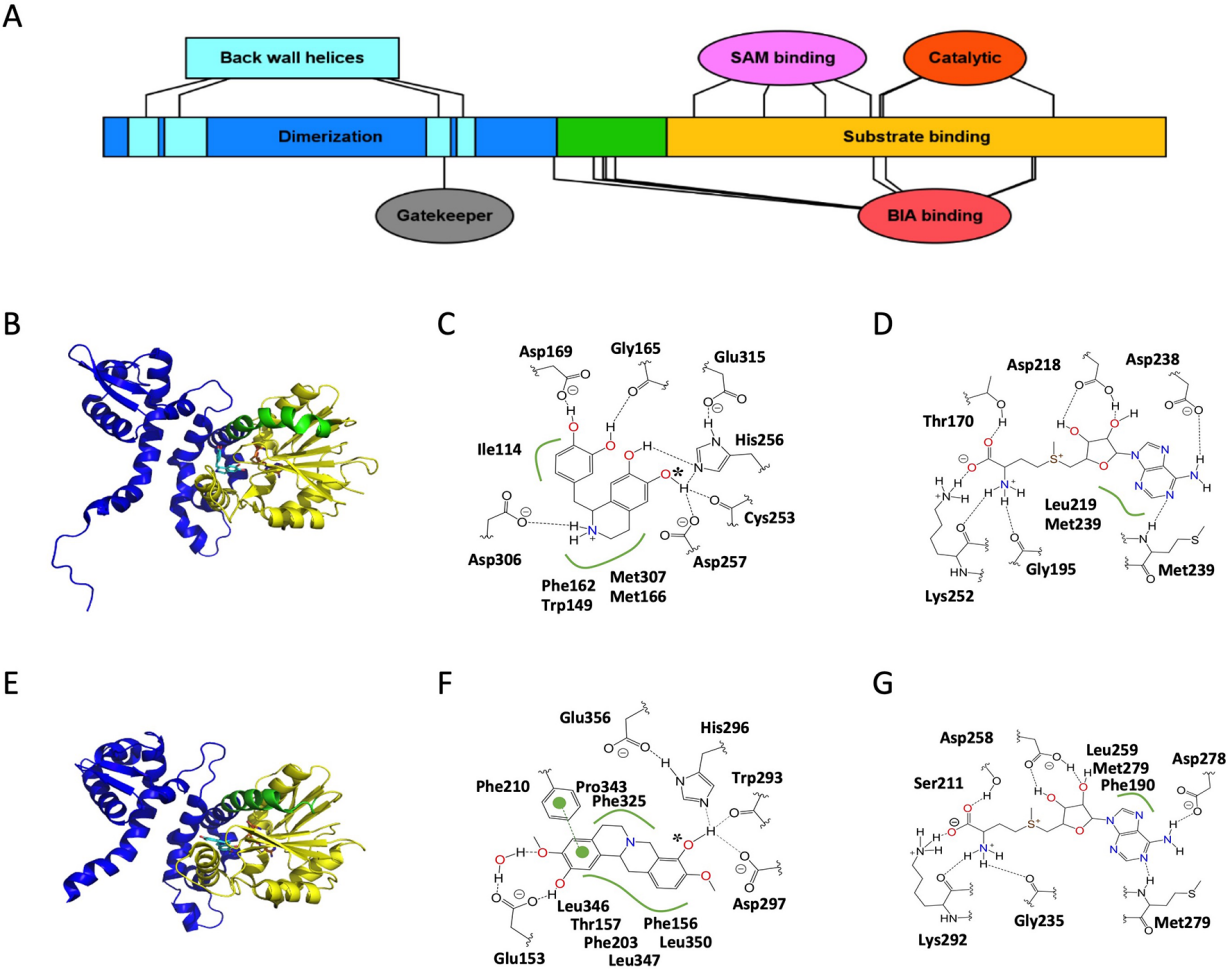
Structure and major active site interactions of representative BIA *O*-methyltransferases. **(A)** Key features of BIA OMTs are labeled according to their relative location along the *T. flavum* norcoclaurine-6-*O*-methyltransferase (Tf6OMT) polypeptide with residues denoted by ovals and larger features by rectangles. Crystal structures of **(B)** Tf6OMT with bound SAH and (*S*)-norlaudanosoline by Robin et al. (2016) (PDB 5ICE), and **(E)** *P. somniferum* scoulerine OMT1 (PsSOMT1) with bound SAH and (*S*)-scoulerine reported by Cabry et al. (2019) (PDB 6I6K). Domains correspond to those shown in **(A)** and **Supplementary Figure 3**. The alkaloid substrate is shown in *cyan*, and the cosubstrate SAH is shown in *orange*. Major active site interactions in **(C, D)** Tf6OMT and **(F, G)** PsSOMT1 were visualized using PoseView and modified where necessary to reflect literature reports. Dashed lines indicate hydrogen bonds, and green lines indicate hydrophobic or aromatic interactions. Dashed lines drawn in *gray* indicate multiple alternative potential interactions. The oxygen atom accepting the methyl group is identified with an asterisk. **(A)** Drawn using Illustrator for Biological Sequences (Liu et al., 2015).

BIA OMTs form dimers in solution as well as in all obtained crystal structures. Dimerization occurs *via* a substantial (150 of 350 residues in Tf6OMT) domain composed primarily of intertwined helices (**Figure 3**). Most of these helices interact with those of the other monomer, resulting in burial of approximately 22% of total protein surface area. Across BIA OMTs, the entire dimerization domain shows relatively modest conservation. Nevertheless, two leucine residues (Leu28, Leu73) show perfect identity, and an additional four hydrophobic residues (Leu34, Ile40, Leu52, Leu66) show strong conservation (**Supplementary Figure 3**). However, their contributions to the dimer interface do not appear to be substantial, suggesting that they may be more important in maintaining secondary and tertiary structures of each monomer (PDBePISA) (Krissinel and Henrick, 2007). Surprisingly, gel filtration analysis of CjSOMT leads authors to report the existence of a trimer in solution (Morishige et al., 2000). Examination of the CjSOMT sequence does reveal a 20-amino acid extension of the N-terminus relative to Tf6OMT. However, it is unclear how this feature could so drastically alter how the monomers associate.

Although heterologously expressed BIA OMTs have generally been studied under the assumption that they form homodimers *in planta*, several studies suggest that dimerization of genetically distinct monomers (i.e., heterodimerization) may play a crucial role in alkaloid biosynthesis (Frick and Kutchan, 1999; Li and Smolke, 2016; Park et al., 2018). Recently, the missing methylation step in *P. somniferum* noscapine biosynthesis was shown to involve a heterodimer of PsSOMT2 and either PsSOMT3 or Ps6OMT (Park et al., 2018). The discovery of an OMT heterodimer that catalyzes a reaction not performed by either homodimer reveals an unexpected diversification strategy by which additional OMT activities can exist without the genomic and metabolic burden of maintaining and expressing dedicated OMT genes. Although the extent of heterodimerization in plant specialized metabolism remains to be established, it is intriguing to consider that many more OMT activities might be linked to heterodimers in the future.

Several helices of the dimerization domain also contribute to the active site, which is found at the interface between the first monomer’s C-terminal domain, two helices of its N-terminal domain, and two helices belonging to the N-terminal domain of the second monomer (**Figure 3**). Together, these four helices form a hydrophobic “back wall” at the active site, but no direct interactions with the substrate have been reported. The lack of clear interactions is surprising given several pieces of evidence showing that the N-terminal domains of both monomers make contributions to substrate selectivity. For example, a chimeric enzyme fusing the Cj6OMT dimerization domain to the C-terminal domain of Cj4′OMT displayed substrate- and regio-specificity most similar to Cj6OMT (Morishige et al., 2010). Further shuffling of the two polypeptide sequences suggested that the determinants of function exist between the 34th and 125th amino acids in Cj6OMT, which is a region that includes the two helices mentioned above, which contribute to a monomer’s own active site, but excludes the two helices, which contribute to the second monomer’s active site. Other lines of evidence suggest that the identity of the second monomer, potentially mediated by the aforementioned two helices, alters substrate specificity in the first monomer’s active site. It was shown that although the PsSOMT2 monomer contains the catalytic machinery necessary for turnover in the PsSOMT2:PsSOMT3 heterodimer, substitution of PsSOMT3 with either PsSOMT2 or PsN7OMT abolished activity (Park et al., 2018). Structural and functional mutagenesis studies should help clarify the origin of these indirect effects.

#### SAM Binding

Most of the BIA OMT polypeptide (200 of 350 residues in Tf6OMT) forms a *C*-terminal domain composed of alternating alpha helices and beta sheets which together create a Rossmann fold classically associated with nucleotide binding (**Figure 3**). SAM binding occurs *via* a series of motifs, which are highly conserved with other plant SAM-dependent MTs (Kozbial and Mushegian, 2005; Gana et al., 2013). The residues within these motifs, which directly interact with SAM, are almost perfectly conserved in known BIA OMTs. However, three SOMTs display conservative (e.g., Asp to Glu) or semi-conservative (e.g., Asp to Gln) substitutions at two positions (**Supplementary Figure 3**). Unfortunately, neither of these substitutions occurs in the recently crystallized PsSOMT1, leaving the question of how these might alter OMT–SAM interactions unresolved. Comparison of apo-enzyme and enzyme–substrate complexes further reveals that the residue equivalent to Thr170 in Tf6OMT (Ser211 in PsSOMT1) interacts with the co-substrate upon binding and contributes to a substantial conformational change (16° hinge movement) of the enzyme likely to be crucial for catalysis (Robin et al., 2016). Intriguingly, binding of SAH was sufficient to induce the closing movement in Tf6OMT, whereas the equivalent movement was only observed after binding of both SAH and the BIA substrate in PsSOMT1 (Cabry et al., 2019). Despite this minor difference, the available evidence suggests that SAM binding occurs in a very similar manner for all BIA OMTs.

#### BIA Binding

Binding of the alkaloid substrate occurs at a location proximal to the SAM binding site and *via* residues overlapping the aforementioned conserved SAM-binding motifs (**Figure 3**; **Supplementary Figure 3**). Notably, the relative orientation of the bound BIA molecule is flipped between Tf6OMT and PsSOMT1, such that isoquinoline and benzyl moieties or their derivatives in protoberberines (**Supplementary Figure 4**; **Supplementary Figure 5**) generally make reciprocal interactions in either enzyme (Robin et al., 2016; Cabry et al., 2019). A histidine residue (His256 in Tf6OMT), in which hydrogen bonds with the target hydroxyl group, is perfectly conserved across BIA OMTs. In Tf6OMT, two closely adjacent residues (Asp257 and Cys253) also interact with the target hydroxyl group and likely fine tune its position and reactivity. On the other hand, the equivalent residues in PsSOMT1 (Asp297 and Trp293) were interpreted as forming a channel through which the SAM methyl group is directed toward the acceptor hydroxyl. Although Asp297 is not proposed to be catalytic *per se*, alanine substitution at this position sharply reduced the activity of PsSOMT1 (Cabry et al., 2019). Precise substrate positioning within the active site is also affected by hydrogen bonds formed between non-targeted moieties and residues equivalent to Gly165 (main chain carbonyl) and Asp169 in Tf6OMT. Whereas the former shows strong semi-conservation (i.e., Gly or Ala), the latter is much more variable while maintaining conservation within certain BIA OMT subtypes (e.g., Glu in 4′OMTs, Asp in 6/7OMTs, His/Phe/Gly in SOMTs). In PsSOMT1, the equivalent residue (Phe210) interacts with a substrate aromatic ring rather than a hydroxyl group.

As seen in the Tf6OMT structure with bound (*S*)-norlaudanosoline, only a small adjustment in the angle of the substrate is required to place the 7-hydroxyl in an alignment productive for methyl transfer and, in fact, 7-*O*-methylation activity has not been ruled out for Tf6OMT (**Figure 3**) (Robin et al., 2016). Conversely, PsSOMT1 has been shown to catalyze both 9- and 2-*O*-methylation of (*S*)-scoulerine despite this requiring the substrate to bind in two completely different orientations (Dang and Facchini, 2012). In the absence of crystal structures revealing the details of such alternative binding modes, biophysical modeling supported by mutagenesis studies may be the only practical method by which to understand the determinants of regio-specificity in BIA OMTs.

In Tf6OMT, Asp306 makes a particularly important contribution to alkaloid binding *via* a hydrogen bond with the nitrogen atom (Robin et al., 2016). A close examination of sequence variation among known BIA OMTs at this position reveals a possible explanation for selectivity concerning *N*-methylation status (i.e., un-methylated secondary or mono-methylated tertiary). Whereas PsN7OMT has an aspartic acid residue capable of hydrogen bonding with the secondary nitrogen atom of (*S*)-norreticuline, Ps7OMT and Ec7OMT have leucine and glycine residues instead (**Supplementary Figure 3**). These smaller and uncharged residues might be expected to alleviate steric hindrance, which would occur with the additional methyl group present on tertiary BIAs like (*S*)-reticuline, thus facilitating binding and catalysis. Although the residue equivalent to Asp306 shows strong conservation among BIA OMTs targeting substrates with the simple 1-BIA scaffold (i.e., 6OMT, N7OMT, 4′OMT, etc.), the equivalent is typically hydrophobic (e.g., Leu or Ala) in SOMT-like enzymes. Examination of the PsSOMT1 structures reveals that this substitution pattern likely relates to the “flipped” substrate-binding mode described above. Rather than being positioned near the nitrogen atom, the equivalent residue (Leu346) simply forms a hydrophobic interaction with carbon atoms in one of the adjacent rings.

A number of more generic interactions are also involved in OMT-BIA binding. Two almost perfectly conserved methionine residues (Met166 and Met307 in Tf6OMT) support and position the rings of the isoquinoline moiety *via* sulfur–aromatic interactions (Reid et al., 1985; Robin et al., 2016). The equivalent residues in PsSOMT1 (Met207 and Leu347) instead sandwich the aromatic ring, which derives from the benzyl moiety in 1-BIAs (**Supplementary Figure 3**; **Supplementary Figure 4**) (Cabry et al., 2019). Similarly, two well-conserved aromatic residues (Phe162 and Trp149 in Tf6OMT, Phe190 and Phe203 in PsSOMT1) provide aromatic interactions with the isoquinoline moiety in Tf6OMT but with the benzyl moiety derivative in PsSOMT1. An additional hydrophobic residue (Ile114 in Tf6OMT, Thr157 in PsSOMT1), which helps position the benzyl or isoquinoline moieties, shows much less conservation. Intriguingly, the enzymes, which catalyze *O*-methylation of the benzyl moiety (e.g., Cj4′OMT, Tf4′OMT, Ps4′OMT2) are substituted with a methionine at this position. Given the observation that two methionine residues help position the isoquinoline moiety in Tf6OMT, it is tempting to speculate that an equivalent interaction takes place in 4′OMTs albeit with the BIA molecule positioned such that the isoquinoline and benzyl moieties swap places. This hypothesis is supported by the “flipped” BIA binding pose of PsSOMT1, which reportedly *O*-methylates the benzyl moiety of certain 1-BIAs (Dang and Facchini, 2012). Crystal structures of 4′OMTs will be helpful in testing this hypothesis. More generally, the size and shape of the substrate pocket is thought to control selectivity at a coarse level (e.g., between BIAs, chalcones, or isoflavones). In particular, the bulky Phe156 in PsSOMT1 was recently proposed to act as a “gatekeeper” residue preventing, *via* steric hindrance, binding of substrates with large groups opposite the target hydroxyl (Cabry et al., 2019). In Tf6OMT, a much smaller residue (Thr113) shapes the binding pocket such that the bulkier isoquinoline moiety can be accommodated. This model is an important first step in establishing a unified framework by which to understand and predict substrate specificity of plant OMTs *a priori*.

#### Catalysis

The catalytic mechanism of BIA OMTs is thought be conserved with other plant SAM-dependent OMTs (Robin et al., 2016; Cabry et al., 2019). Briefly, a histidine residue (His256 in Tf6OMT, His296 in PsSOMT1) acts as a general base and deprotonates the target hydroxyl group. Subsequently, the newly generated oxyanion carries out a nucleophilic attack on the labile methyl group of SAM, which results in its transfer. The significance of the histidine residue in BIA OMTs was confirmed by targeted mutagenesis experiments, which almost entirely abolished catalytic activity in PsSOMT1 and other *P. somniferum* OMTs (Park et al., 2018; Cabry et al., 2019). An adjacent residue (Asp257 or Asp297), discussed above in the context of substrate binding, can also be thought of as participating in catalysis. In PsSOMT1, substitution of this residue with an alanine yielded an enzyme with roughly 2% activity, leading the workers to conclude that it has an important but non-essential role (Cabry et al., 2019). Structural analysis further implicates a glutamic acid residue (Glu356 in PsSOMT1, Glu315 in Tf6OMT) in hydrogen bonding with the catalytic histidine to promote its necessary basicity. Although the equivalent residue was not discussed with respect to Tf6OMT, examination of their published structures suggests that such an interaction is also present. In fact, perfect conservation of this residue suggests that this aspect of catalysis is maintained in all known BIA OMTs (**Supplementary Figure 3**).

Feedback inhibition is known to be a significant feature affecting BIA OMT activity (Sato et al., 1994; Robin et al., 2016). As shown for Tf6OMT, pathway end products can compete for the active site and thus slow or prevent catalysis. In the case of inhibition by sanguinarine, binding occurs in a position that partially overlaps with that of a productive substrate as described above. Several of the generic interactions (e.g., those with Met166 and Ile114) are preserved, while one of the hydrogen bonding residues (Asp169) interacts with a different O atom. As a result, the planar sanguinarine molecule binds in a position rotated roughly 90° on two axes relative to the productive BIA substrate and forms several new aromatic and hydrogen bonding interactions. Comparison of enzyme–inhibitor interactions to those with a productive substrate can provide rational targets for mutagenesis (i.e., residues interacting with the inhibitor but not the productive substrate), thus potentially allowing for the engineering of feedback-insensitive OMT enzymes, which would be highly useful in biotechnological applications. Unfortunately, the specifics of these interactions are likely to vary significantly from one enzyme–inhibitor pair to the next, thus limiting our ability to generalize from structures already reported in the literature. Although additional crystal structures with bound inhibitors are the gold standard, biophysical modeling approaches (e.g., docking) could also provide some insight on shorter timescales.

### *N*-Methyltransferases

Compared to the OMTs, BIA NMTs have received substantial attention in terms of structure–function investigations despite the much smaller number of functionally characterized representatives. An initial investigation was reported for *T. flavum* pavine NMT (PDB 5KOK) and, recently, for *C. japonica* coclaurine NMT (PDB 6GKV) (Torres et al., 2016; Bennett et al., 2018). A third report concerning the tetrahydroprotoberberine NMT from *G. flavum* was accepted for publication during the preparation of this manuscript (PDB 6P3O) (Lang et al., 2019). Taken together, these three studies cover much of the functional range reported to date for BIA NMTs and reveal many of the molecular determinants of function.

**Figure 4:**
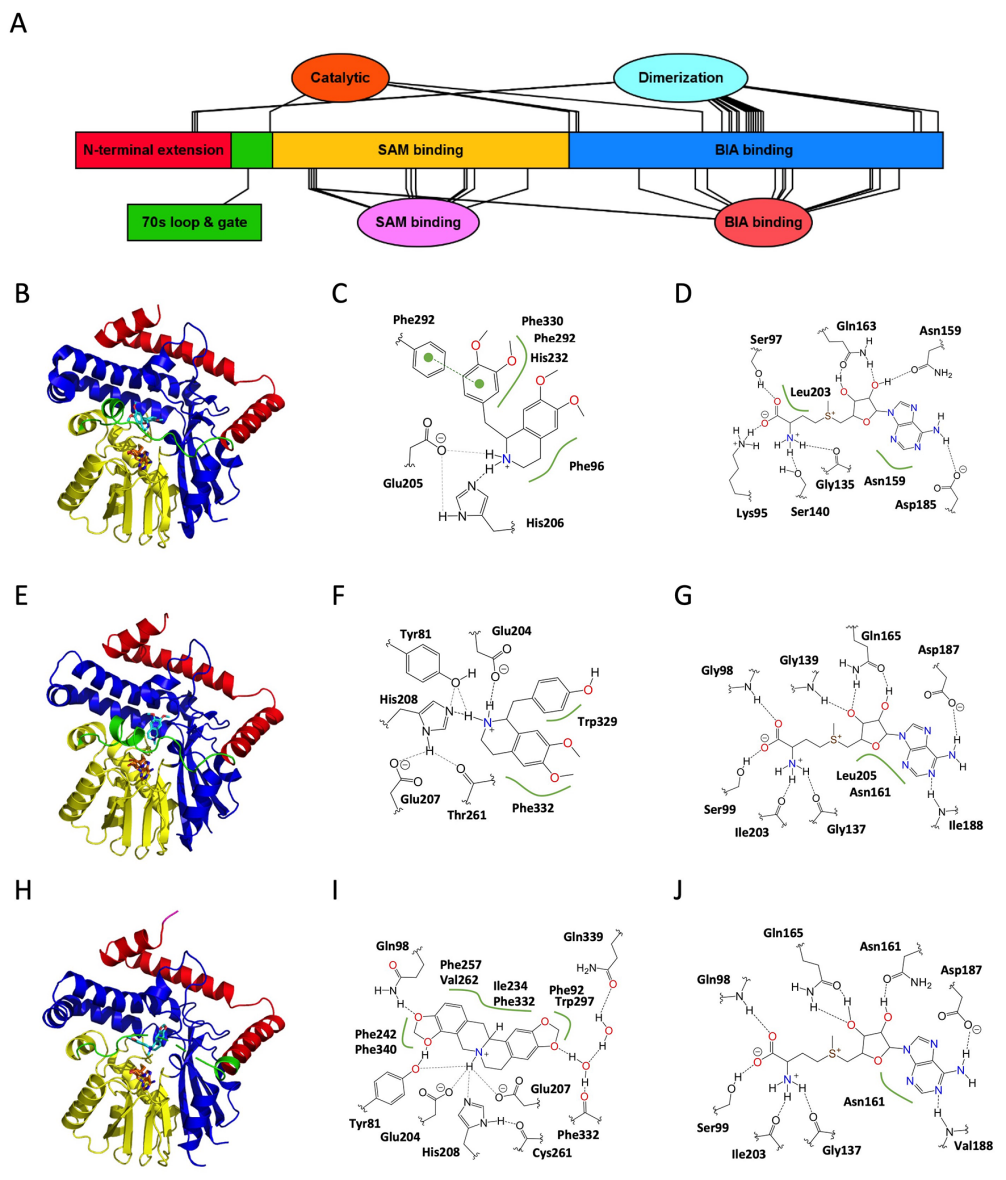
Structure and major active site interactions of representative BIA *N*-methyltransferases. **(A)** Key features of BIA NMTs are labeled according to their relative location along the *T. flavum* pavine *N*-methyltransferase (TfPavNMT) polypeptide with residues denoted by ovals and larger features by rectangles. **(B)** Crystal structures of TfPavNMT with bound SAH and (*R,S*)-tetrahydropapaverine are reported by Torres et al. (2016) (PDB 5KOK), **(E)** *C. japonica* coclaurine NMT with bound SAH and *N*-methylheliamine is reported by Bennett et al. (2018) (PDB 6GKV), and **(H)** *G. flavum* tetrahydroprotoberberine NMT with bound SAH and (*S*)-*cis*-*N*-methylstylopine is reported by Lang et al. (2019) (PDB 6P3O). Domains correspond to those shown in **(A)** and **Supplementary Figure 6**. The alkaloid substrate is shown in *cyan*, and the cosubstrate SAH is shown in *orange*. Major active site interactions in **(C, D)** TfPavNMT, **(F, G)** CjCNMT, and **(I, J)** GfTNMT were visualized using PoseView and modified where necessary to reflect literature reports. Dashed lines indicate hydrogen bonds, and green lines indicate hydrophobic or aromatic interactions. Dashed lines drawn in *gray* indicate multiple alternative potential interactions. **(A)** Drawn using Illustrator for Biological Sequences (Liu et al., 2015).

The overall structures (**Figure 4**; **Supplementary Figure 6**), which include a canonical Rossmann SAM-binding domain as well as a C-terminal substrate-binding domain, are consistent with those reported for other SAM-dependent NMTs including *Plasmodium *phosphoethanolamine NMT (PfPMT; PDB 3UJA) and *Mycobacterium tuberculosis* cyclopropane synthase (MtPcaA; PDB 1KPH) (Huang et al., 2002; Lee et al., 2012). Although we annotate the BIA NMT polypeptide sequences with distinct SAM and BIA substrate-binding regions for simplicity, interacting residues are not strictly found within these regions, and domains become more evident in the tertiary structure. In addition to the typical NMT domains, the BIA NMTs also contain an N-terminal extension composed of three helices, which wrap around the substrate-binding domain and contribute to a homodimerization interface. This N-terminal extension also contributes to positioning a loop and helix proposed to gate the active site.

#### Dimerization

As with the OMTs, BIA NMTs form dimers both in solution and under crystallization conditions (Torres et al., 2016; Bennett et al., 2018). However, the extent of the dimerization interface is substantially less and corresponds to only 6.5% and 6.9% of surface area in TfPavNMT and CjCNMT, respectively. Although buried surface area is similar in GfTNMT, the occurrence of several salt bridges renders the dimerization substantially more favorable, suggesting that the importance of dimerization may vary across BIA NMTs. In all cases, dimerization occurs at the “rear” of the monomer with respect to the substrate-binding pocket and *via* somewhat conserved residues located primarily in two helices and two beta sheets distributed between the N-terminal extension and C-terminal BIA-binding domain. Notably, reciprocal interactions between tri-lysine motifs present in the C-terminus of all BIA NMTs also contribute to the dimerization interface. In Arabidopsis and other eukaryotes, such a motif has been shown to result in retrograde trafficking (ER retention) of membrane-bound proteins *via* interaction with COPI proteins (Wang et al., 2011). However, given that BIA NMTs have been experimentally shown to localize to the cytosol, it appears that this motif may have a more general utility in enabling protein–protein interaction (Hagel and Facchini, 2012). Neither the *in planta* occurrence or functional significance of homo- or hetero-dimerization has been verified for BIA NMTs. However, it has been speculated that small hinge movements in one monomer might be transferred *via* the dimerization domain to the other monomer, resulting in a cooperativity effect. Nevertheless, in the absence of close interactions between the dimerization domain and catalytic site, it would be surprising to discover consequences as substantial as those discussed for the OMTs.

#### SAM Binding

The Rossmann fold SAM-binding domains of various BIA NMTs are structurally quite similar, yielding RMSD values of ∼0.4–0.5 Å when aligned to each other (Torres et al., 2016; Lang et al., 2019). Binding of the cosubstrate occurs *via* sequence motifs largely conserved with other plant SAM-dependent MTs (Kozbial and Mushegian, 2005; Gana et al., 2013). In the available crystal structures, up to 13 direct or water-mediated hydrogen bonds appear to position the cosubstrate (**Figure 4**). The implicated residues are almost perfectly conserved in all cloned BIA NMTs, with the exception of one non-conservative substitution (Gln to His) in PsRNMT (**Supplementary Figure 6**) (Torres et al., 2016; Bennett et al., 2018; Lang et al., 2019). Comparison of the apoenzyme and binary complexes (e.g., TfPavNMT *versus* TfPavNMT + SAH) revealed only a minor hinge movement of domains relative to that seen in the OMTs. Instead, active site closure seems to depend on the gate-like 70s loop, which becomes more ordered upon cosubstrate binding. Sequence identity in these features strongly suggests that the mechanisms of SAM binding are conserved for all known BIA NMTs.

#### BIA Binding

As expected, given the diversity of substrates turned over by various BIA NMTs, the largely helical BIA binding domain shows substantially less conservation than the SAM-binding domain. Although crystal forms binding the enzymes’ preferred or physiological substrates (e.g., reticuline or pavine for TfPavNMT, coclaurine for CjCNMT, stylopine for GfTNMT) have been elusive, structures with bound analogs (e.g., *N*-methylheliamine, tetrahydropapaverine), nevertheless, provide some insight into substrate recognition and catalysis. Fortuitously, a GfTNMT crystal was obtained in complex with the endogenous product (*S*)-*cis*-*N*-methylstylopine (SMS), providing the most reliable view to date of biologically meaningful interactions. In all BIA NMTs, the binding pocket is lined with many hydrophobic residues and apparently gated by a loop, which becomes ordered and partially helical upon substrate binding. While a limited number of hydrogen bonds are recognizable in the GfTNMT crystal structure, as a general rule, it appears that substrate recognition and binding depends primarily on steric effects, Van der Waals interactions, and aromatic interactions (**Figure 4**) (Torres et al., 2016; Lang et al., 2019).

The availability of structures for three BIA NMTs reveals that the key substrate-binding interactions differ somewhat across functional subtypes. In TfPavNMT, 13 residues line the binding pocket, and the most significant substrate interactions involve Phe96, His232, Phe292, and Phe330. In CjCNMT, three residues lining one side of the binding pocket (Tyr328, Trp329, Phe332) were probed by site-directed mutagenesis and shown to significantly impact function (Bennett et al., 2018). Characterization of the mutant enzymes’ kinetics with respect to isoquinoline and benzylisoquinoline substrates revealed that Trp329 primarily interacts with the benzyl moiety, whereas Phe332 primarily interacts with the isoquinoline moiety. In GfTNMT, comparable results were obtained in which substitution of the equivalent residues (Ile329 and Phe332) with alanine resulted in mutant enzymes with roughly 10% activity (Lang et al., 2019). While GfTNMT’s lack of activity with isoquinoline substrates precluded a comparative kinetic analysis such as was carried out for CjCNMT, examination of the GfTNMT crystal structure allowed for the authors to suggest that Phe332 is better positioned to interact with the benzyl moiety and associated C9/C10 methylenedioxy bridge of SMS.

In contrast to the other structurally characterized BIA NMTs, GfTNMT does appear to form a limited set of hydrogen bonds with the BIA molecule’s functional groups. Whereas the side chains of residues Gln98 and Tyr81 interact directly with the oxygen atoms of the C9/C10 methylenedioxy bridge in SMS, residues Phe332 (main chain carbonyl) and Gln339 (side chain carboxyamide) interact with the C2/C3 oxygen atoms *via* a network of water-mediated hydrogen bonds. Substitution of Tyr81with either phenylalanine or alanine resulted in comparable mutant enzymes displaying 10–20% activity, indicating that the hydroxyl group of the tyrosine side chain is crucial to the residue’s function (Lang et al., 2019). While the involvement of water molecules in substrate binding was only explicitly reported for GfTNMT, careful examination of the TfPavNMT and CjCNMT crystal structures reveals the existence of several well-ordered water molecules within potential hydrogen bonding distance of the BIA molecule.

Interestingly, the size of the substrate-binding pocket in TfPavNMT is somewhat larger than that reported for the other BIA NMTs (Torres et al., 2016). Enlargement of the cavity apparently results from many small contributions, including individual amino acid substitutions (e.g., A204), rotation of side chains and slight repositioning of secondary structural elements (e.g., helix α4, 240s loop). Although two separate molecules of a 1-BIA substrate were bound in the reported structure, it was suggested that BIA dimers (bisbenzylisoquinolines) common in *Thalictrum* spp. might bind to the active site *in planta*. While bis-BIAs might conceivably be substrates for *N*-methylation, their status as pathway end products makes it tempting to speculate that they could instead act as feedback inhibitors in a manner analogous to that described for sanguinarine and Tf6OMT. Alternatively, binding of a second BIA monomer at the adjacent (non-productive) location in the active site might cause either substrate or product inhibition and thus regulate NMT activity and adjust pathway flux. Given that binding of a second 1-BIA molecule was proposed to be inhibitory in TfPavNMT, it is reasonable to suspect that the active site might be structured differently when a single substrate molecule is bound. One likely possibility is a tighter interaction with the 70s loop “gate”, which is notably displaced in the available TfPavNMT structure relative to CjCNMT.

In sum, the substrate-binding interactions presently described in the literature vary quite a bit despite substantial conservation of active site residues and domain structure. Given that crystal structures are only available for one representative of each BIA NMT subtype (e.g., TNMT, CNMT, RNMT/PavNMT), it is not presently clear whether the details of substrate binding are conserved across functionally analogous enzymes from different species (e.g., TfCNMT, CjCNMT, PsCNMT, GfNMT1). However, comparably low levels of sequence conservation (i.e., 45–80% identity) make it reasonable to suspect that the specific interactions might be variable. Accordingly, attempts to generalize from available structural information may be misleading, and additional studies are warranted.

#### 70s Loop “Gate”

The 70s loop or active site “gate” mentioned above is a particularly intriguing feature that is not well understood. This region of the polypeptide undergoes a transition from disordered to ordered form (including structuration of helix α4) upon SAM and BIA binding, yet no direct interactions are made with the substrate (Torres et al., 2016, Bennett et al., 2018). Nevertheless, patterns of sequence conservation within BIA NMT subtypes (e.g., CNMTs *versus* TNMTs) strongly suggest that the gate contributes to functional differences (**Supplementary Figure 6**). To date, no mutagenesis studies have examined the functional consequences of these variable residues.

On the other hand, two residues adjacent to the 70s loop, which show perfect conservation among the BIA NMTs have been mutagenized with dramatic outcomes. Unexpectedly, replacement of Glu80 in TfPavNMT with alanine resulted in a substantial increase in activity, which was especially notable for non-endogenous substrate (*R,S*)-tetrahydropapaverine (Torres et al., 2016). In GfTNMT, mutagenesis of the equivalent Glu82 leads to a decrease in all activities. However, the effect was comparable in the sense that the mutant enzyme’s substrate preference was shifted in favor of scoulerine, which is not considered to be an endogenous substrate for TNMT *in planta*. Examination of crystal structures shows that Glu80/Glu82 hydrogen bonds with an adjacent helix and suggests that the residue might contribute to substrate selectivity by anchoring the 70s loop. The second adjacent residue, Tyr79/Tyr81, is less consistently positioned across BIA NMTs, and mutagenesis also had variable effects depending on the enzyme–substrate pair. In CjCNMT and GfTNMT, the tyrosyl side chain points into the active site and might directly interact with the BIA amino group or benzyl moiety functional groups (**Figure 4**). GfTNMT mutants in which Tyr81 was replaced with alanine, phenylalanine, or arginine all showed substantially reduced activities, indicating that a rather specific interaction (likely involving a hydrogen bond) takes place in the wildtype enzyme. In TfPavNMT, the Tyr79 side chain is rotated approximately 45° with reference to the other BIA NMT structures and does not directly interact with the substrate. Interestingly, replacement of this residue with alanine almost entirely abolished activity with a potential endogenous substrate ((*S*)-reticuline) but activity with the non-endogeous (*R,S*)-tetrahydropapaverine was greatly enhanced. Together, these pieces of evidence suggest that Tyr79/Tyr81 also contributes to selectivity for particular substrates. Thus, it is clear that the 70s loop “gate” and adjacent residues have important consequences regarding BIA NMT activity. While it appears that the outcome of these contributions is substrate selectivity, the precise mechanisms by which this occurs remain to be elucidated.

#### Catalysis

The mechanisms of catalysis in BIA NMTs are not yet entirely resolved, but appear similar in some ways to that described above for BIA OMTs (Torres et al., 2016; Bennett et al., 2018; Lang et al., 2019). Perfectly conserved glutamic acid and histidine residues are implicated in the methyl transfer reaction, although their contributions may vary from one enzyme–substrate pair to the next. The histidine residue is proposed to act as a general base, which deprotonates the substrate nitrogen and thus activates it to carry out nucleophilic attack on the labile methyl group of SAM (Bennett et al., 2018). In support of an important role for the histidine residue, substitution of His206 in TfPavNMT and His208 in CjCNMT with alanine resulted in mutant enzymes with sharply reduced activities. Nevertheless, activity was not entirely abolished, and so, it appears that, unlike in BIA OMTs, deprotonation by a general base mechanism is not strictly necessary for catalysis.

Several proposals have been put forth concerning the function of the adjacent glutamic acid residue. In analogy to the catalytic dyad of BIA OMTs (i.e., histidine and aspartic acid), the glutamic acid carboxyl group might hydrogen bond with the histidine imidazole moiety and thus promote the basicity required for substrate deprotonation. However, given that mutagenesis of Glu207 in CjCNMT had only a minor effect on activity (∼35% decrease), it was proposed that the backbone carbonyl of another highly conserved residue (Thr261 in CjCNMT) might be the catalytic partner. Another possible mechanism for the glutamic acid is hydrogen bonding with the BIA nitrogen atom to improve its position and orientation relative to the incoming methyl group. Similarly, the glutamic acid might hydrogen bond with the methyl group, itself, in a manner that stabilizes the reaction intermediate, as has been proposed for rat glycine NMT on the basis of computational modeling (Świderek et al., 2018). Aside from hydrogen bond-mediated interactions, it has also been proposed that the reaction intermediate could be stabilized by electrostatic interactions with the negatively charged glutamic acid side chain. Experimental results, in which double mutants (e.g., TfPavNMT-Glu205Ala-His206Ala) show an additive effect, are consistent with any of these alternative models in which the key active site residues make independent contributions to catalysis.

Notably, a general base mechanism may not be applicable to catalysis of all substrates accepted by BIA NMTs. For example, the tertiary amine in GfTNMT substrate stylopine has a calculated pKa of approximately 5.3, which indicates that it would be almost entirely deprotonated under physiological pH conditions, and thus, proton abstraction would be unnecessary to allow methyl transfer (www.chemicalize.org). Mutagenesis of His208 and Glu207 in GfTNMT had effects comparable to those seen in other BIA NMTs (for which substrates almost certainly require deprotonation), indicating that these residues are important for catalysis even when a general base mechanism cannot be invoked.

A fourth residue showing intriguing patterns of conservation between BIA NMT subtypes has also been shown to interact with the substrate (Glu204 in CjCNMT) (Bennett et al., 2018). Due to the side chain’s proximity to the alkaloid nitrogen atom and the substantial detrimental effect of alanine substitution on catalysis, Micklefield and colleagues proposed that Glu204 hydrogen bonds with, and helps fine-tune the position and reactivity of, the target nitrogen atom. Interestingly, alanine substitution of Glu204 in GfTNMT had a comparable effect on activity with stylopine despite this molecule’s nitrogen atom being unable to form an equivalent hydrogen bond concurrent with methyl transfer. Accordingly, in GfTNMT, it appears more likely that Glu204 interacts with the transition state methyl group or contributes an electrostatic effect. Given that the equivalent residue in TfPavNMT is an alanine, it is clear that the details of this interaction are not conserved between BIA NMT functional subtypes. Intriguingly, RNMT-like enzymes, which efficiently catalyze *N*-methylation of tertiary BIAs (e.g., reticuline) have a glycine at this position (Morris and Facchini, 2016). The absence of a side chain likely alleviates steric hindrance, which would be expected to occur between the *N*-methyl group and the glutamic acid present in CNMTs. Mutational studies, as well as investigation of natural variation of this position in diverse BIA NMTs, should allow for this hypothesis to be tested.

Although the above proposals likely explain a significant portion of BIA NMT catalytic power, experimental results clearly show that no residue identified to date (Glu204, Glu207, His208, and equivalents) is strictly necessary for methyl transfer to occur. This is in notable contrast to mutagenesis studies on BIA OMTs in which substitution of the general base histidine precludes all activity (Park et al., 2018; Cabry et al., 2019). In fact, even the simultaneous replacement of up to three putative catalytic residues with alanine produced mutant TfPavNMT and GfTNMT enzymes with detectable activities (Torres et al., 2016; Lang et al., 2019). While it remains possible that a general base mechanism is at play in certain reactions, the aforementioned work strongly suggests that rate enhancement in BIA NMTs results from multiple modest contributions. Mechanisms consistent with this interpretation include electrostatic or hydrogen bond-mediated stabilization of the reaction intermediate, and those which invoke “compression, proximity, orientation and desolvation” effects (Zubieta et al., 2003). While the identification and mutagenesis of additional catalytic residues may assist in resolving this question, biophysical modeling may be more likely to provide a conclusive answer.

## Evolution of Diversity in BIA Methyltransferases

Compared to our detailed understanding of the evolutionary trajectories of MT activities implicated in caffeine biosynthesis, relatively little equivalent knowledge is presently available concerning the BIA MTs. Nevertheless, the recent rapid increase in availability of sequence, structure, and function information for these important enzymes foreshadows a commensurate leap forward in our understanding. Given the substantially more complex pathways involved in BIA biosynthesis, understanding the evolution of even a subset of the enzymes will greatly enhance our conceptual grasp of how the tremendous chemodiversity in plants originates.

### *O*-Methyltransferases

**Figure 5:**
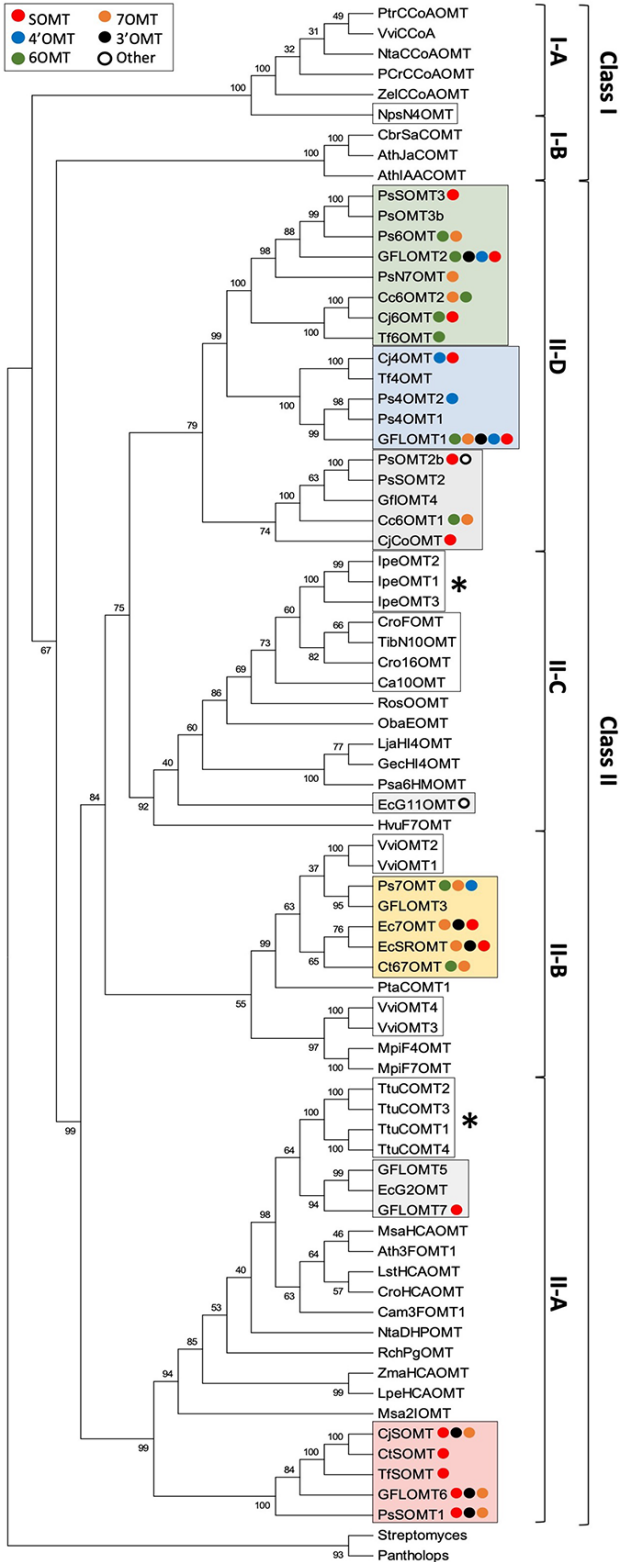
Rooted neighbor-joining phylogenetic tree of BIA *O*-methyltransferases and representative plant *O*-methyltransferases labeled with reported *in vitro* substrate range. Frequencies shown at each node represent the percentage of 500 bootstrapped replicate trees in which the associated taxa clustered together. The consensus tree is shown without branch lengths. The analysis involved 77 sequences, and positions with less than 50% coverage were discarded, resulting in a final dataset with 353 positions. The tree was rooted with sequences from bacteria and animals. The analysis was conducted in MEGA7 (Kumar et al., 2016). Representative plan OMT sequences and clade assignments were adapted from Lam et al. (2007). Plant OMTs implicated in the biosynthesis of alkaloids other than BIAs are indicated by white boxes, whereas those that nevertheless accept BIA substrates are indicated with *asterisks*. BIA OMTs are labeled with colored circles representing their reported *in vitro *activities, in order from strongest to weakest (red, scoulerine-*O*-methylation; blue, 4′-*O*-methylation; green, 6-*O*-methylation; yellow, 7-*O*-methylation; black, 3′-*O*-methylation; white, other). Clades with recognizable majority activities are shaded with rectangles in the corresponding color. Genbank accession numbers for BIA OMTs are provided in **Supplementary Table 1**. Other alkaloid OMT accession numbers are: NpsN4′OMT (KJ584561), IpeOMT1 (AB527082), IpeOMT2 (AB527083), IpeOMT3 (AB527084), Ca10OMT (MG996006), Cro16OMT (EF444544), TibN10OMT (MH454075), VviOMT1 (KC533529), VviOMT2 (KC533535), VviOMT3 (KC517470), and VviOMT4 (KC517475).

The importance of *O*-methylation in various aspects of plant metabolism (e.g., lignin, phenylpropanoid, phytoalexin, and phytohormone biosynthesis) makes it unsurprising that their classification and evolutionary relatedness has received substantial attention. Setting aside the highly sequence-divergent SABATH MTs, the remainder of plant OMTs are generally understood to fall within two major groups (Lam et al., 2007). The first (class I) contains mostly enzymes, which target hydroxycinnamoyl-CoA esters and participate in lignin biosynthesis as well as carboxylic acid OMTs involved in plant hormone and scent metabolism, and the second (class II) contains a much more variable set of enzymes including those involved in phenylpropanoid and alkaloid biosynthesis. The first group is thought to have evolved as a result of pressures relating to the colonization of terrestrial habitats, whereas the second group likely arose later in response to a more diverse set of evolutionary forces. Although a focused and up-to-date phylogenetic study of plant OMTs is overdue, basic analyses reported in conjunction with the isolation of new alkaloid OMTs have consistently placed the BIA OMTs within class II (Morishige et al., 2002; Morishige et al., 2010; Nomura and Kutchan, 2010; Dang and Facchini, 2012; Salim et al., 2018). While a complete examination of known plant OMTs is outside the scope of this review, we performed a limited analysis of representative plant OMTs, BIA OMTs, and other recently reported alkaloid OMTs to place them in the context of the phylogeny reported by Dananyandan and colleagues (**Figure 5**) (Lam et al., 2007). Consistent with the previous work, our results place all cloned BIA OMTs within a well-supported clade corresponding to class II. We recovered four subclades with topology and bootstrap support similar to those reported previously. A large majority of BIA OMTs fell within subclade II-D, which was sister to subclade II-C in which most other alkaloid OMTs and flavonoid OMTs clustered. The functionally validated BIA OMTs in subclade II-D appear to be monophyletic, and most prefer substrates of the simple 1-BIA structure (**Supplementary Table 1**). In addition, the heterodimer-forming OMTs participating in noscapine biosynthesis are within this clade, with the two functionally interchangeable monomers (PsOMT3 and Ps6OMT) clustering together. A single putative BIA OMT, for which preliminary reports indicate a unique ability to methylate the free hydroxyl group of 10-hydroxydihydrosanguinarine, falls within clade II-C in a position distant from the other BIA OMTs and apparently more closely related to other alkaloid OMTs (Purwanto, 2017). Many of the remaining BIA OMTs belong to the very well-supported subclade II-A, which also included hydroxycinnamic acid OMTs involved in phenylpropanoid biosynthesis. All of the BIA OMTs in this subclade are functionally characterized as preferring or accepting protoberberine substrates. The smallest and least well-supported subclade (II-B) contained a few remaining BIA OMTs that mostly target the 7-hydroxyl of simple 1-BIAs, along with OMTs involved in flavonoid and methoxypyrazine biosynthesis.

As suggested in previous publications, the presence of several well-separated clades makes it reasonable to suspect that BIA OMTs have evolved repeatedly in plants. However, it should be noted that the various clades of BIA OMTs show distinct substrate- and regio-specificity and thus cannot be said to have converged on precisely the same function (**Figure 5**). Although the most commonly accepted explanation at present is repeated evolution, it remains possible that an ancestral OMT was broadly promiscuous and had activity with BIAs, which was lost in most non-BIA OMT lineages. While this may be less likely given the multitude of non-BIA plant OMTs in existence, certain pieces of evidence suggest that it is a question worth exploring. Phenylpropanoid OMTs cloned from *T. tuberosum* were shown to also accept benzylisoquinoline substrates and, similarly, enzymes implicated in the biosynthesis of ipecac alkaloids in *Psychotria ipecacuanha* catalyze methylation of BIAs with surprising efficiency (Frick and Kutchan, 1999; Nomura and Kutchan, 2010). In fact, the highly divergent and distantly related rat liver catechol OMT is also known to accept BIA substrates (Meyerson et al., 1979). Conversely, none of the BIA OMTs assayed with phenylpropanoid substrates have been shown to accept them (Morishige et al., 2002; Ounaroon et al., 2003; Pienkny et al., 2009), perhaps indicating that such a function is the more derived character. It is intriguing to consider that our present understanding of most plant OMTs as relatively specialized for one class of substrate or another might be a result in part from the practical challenges of assaying for many diverse activities when a new enzyme is discovered. Resolving this important question will require that, going forward, workers begin to routinely examine plant OMTs with respect to a wider range of potential and even physiologically unlikely substrates.

Superimposition of reported *in vitro* activities over apparent phylogeny reveals conservation of function in some BIA NMT clades but not others (**Figure 5**). The existence of one well-separated subclade containing only enzymes, which preferentially methylate protoberberines such as (*S*)-scoulerine suggests that most SOMTs share a monophyletic origin. In all but one case, members of this subclade have also been shown to methylate simple 1-BIA substrates to a lesser degree. While this might be interpreted as maintenance of an ancestral enzyme feature, structural comparison of the flexible 1-BIAs to the rather rigid protoberberines suggests that the former can readily adopt a conformation mimicking the latter, thus potentially explaining the functional overlap from a strictly structural point of view. It is interesting to note that the hydroxyl groups methylated by SOMT-like enzymes in simple 1-BIAs (i.e., 7, 3′) correspond to those methylated in protoberberines (i.e., 2, 9) (**Supplementary Figure 4**). Enzymes with SOMT-like activity are also present in other clades. In particular, the *C. japonica* enzyme shown to prefer columbamine over scoulerine may have evolved independently. Clades with majority preferences for 6-, 7-, or 4′-*O*-methylation of simple 1-BIAs show substantially more functional diversity between members. Although clades showing a preference for 6- or 7-*O*-methylation are recognizable, most enzymes catalyze both reactions. As mentioned above with reference to the Tf6OMT structure, only a minor adjustment of binding angle is necessary to position either the 6- or 7-hydroxyl in a productive alignment for methyl transfer (**Figure 3**). Despite their present functional overlap, the distinct clades suggest that 6/7-*O*-methylation evolved in two independent lineages. Like the SOMTs, enzymes catalyzing 4′-*O*-methylation primarily fall within a single clade indicative of monophyletic origin. Weak 4′ OMT activities also reported for one 6OMT and one 7OMT enzyme may reflect the ability of a simple 1-BIA to bind in a “flipped” orientation as hypothesized in the structural section above. Notably, a clade corresponding primarily to 3′-*O*-methylation is not evident and such enzymes are present in most clades. Although the above evidence suggests that specialization for various BIA OMT activities occurred in several independent lineages, the sporadic occurrence of corresponding but weaker activities in other clades suggests the possibility that an ancestral BIA OMT was highly promiscuous and perhaps able to catalyze the full range of methylations with lower efficiency. Resurrection and functional characterization of ancestral BIA OMTs should help test this hypothesis in the near future.

### *N*-Methyltransferases

*N*-methyltransferases in plants have received less attention overall, and evolutionary relationships are still unclear. It is presently thought that they are polyphyletic in origin and, in fact, that several distinct NMTs may have existed in the last universal common ancestor of all extant life (Anantharaman et al., 2002). Other than the BIA NMTs, major small molecule NMT families in plants include the putrescine NMTs, phosphoethanolamine NMTs, xanthine (SABATH) NMTs, and tocopherol C-methyltransferase-like NMTs (Hibi, 1994; Kato et al., 2000; Nuccio et al., 2000; Liscombe et al., 2010). Given that a comprehensive classification and phylogenetic analysis of plant NMTs is far outside the scope of this review, only the BIA NMT-like enzymes will be considered below.

**Figure 6:**
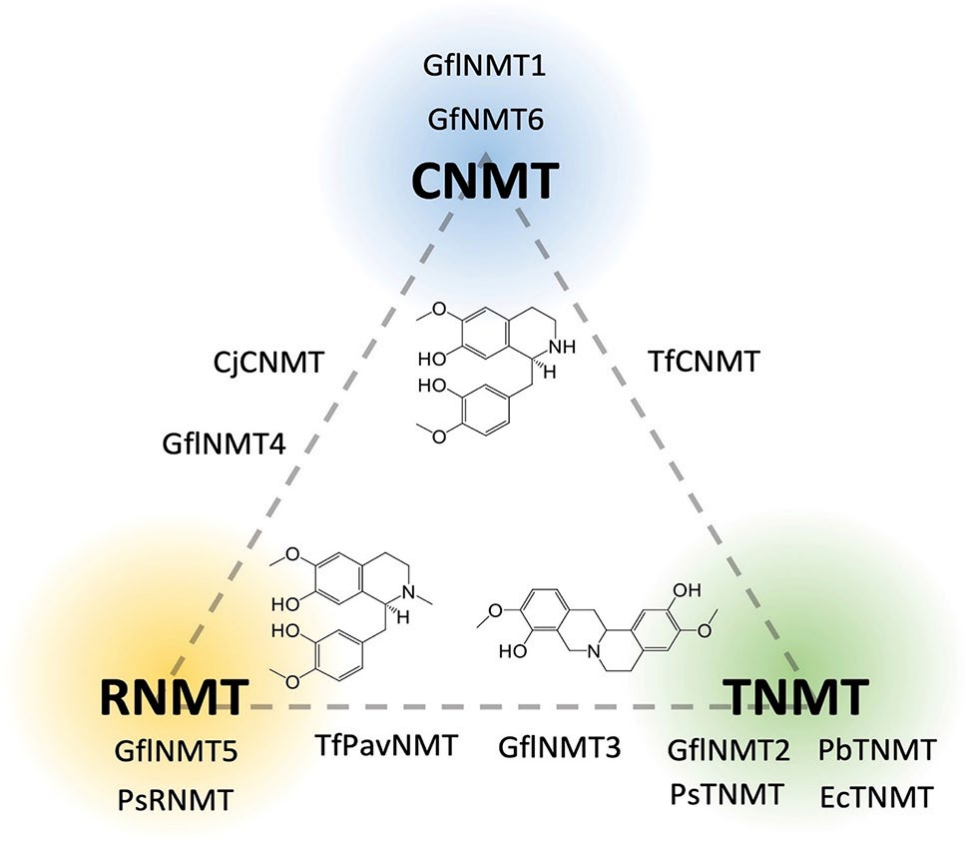
Relative *in vitro* activities of BIA *N*-methyltransferases on a CNMT-RNMT-TNMT gradient. Enzymes are placed on the diagram in positions reflecting their* in* *vitro* activities’ correspondence to one or more of the three major canonical BIA NMT activities as reported in the literature (CNMT, coclaurine *N*-methyltransferase; RNMT, reticuline *N*-methyltransferase; TNMT, tetrahydroprotoberberine *N*-methyltransferase). A representative substrate is shown for each group [CNMT, (*S*)-Coclaurine; TNMT, (*S*)-Scoulerine; RNMT, (*S*)-Reticuline]. Genbank accession numbers and activity details are provided in **Supplementary Table 1**.

**Figure 7:**
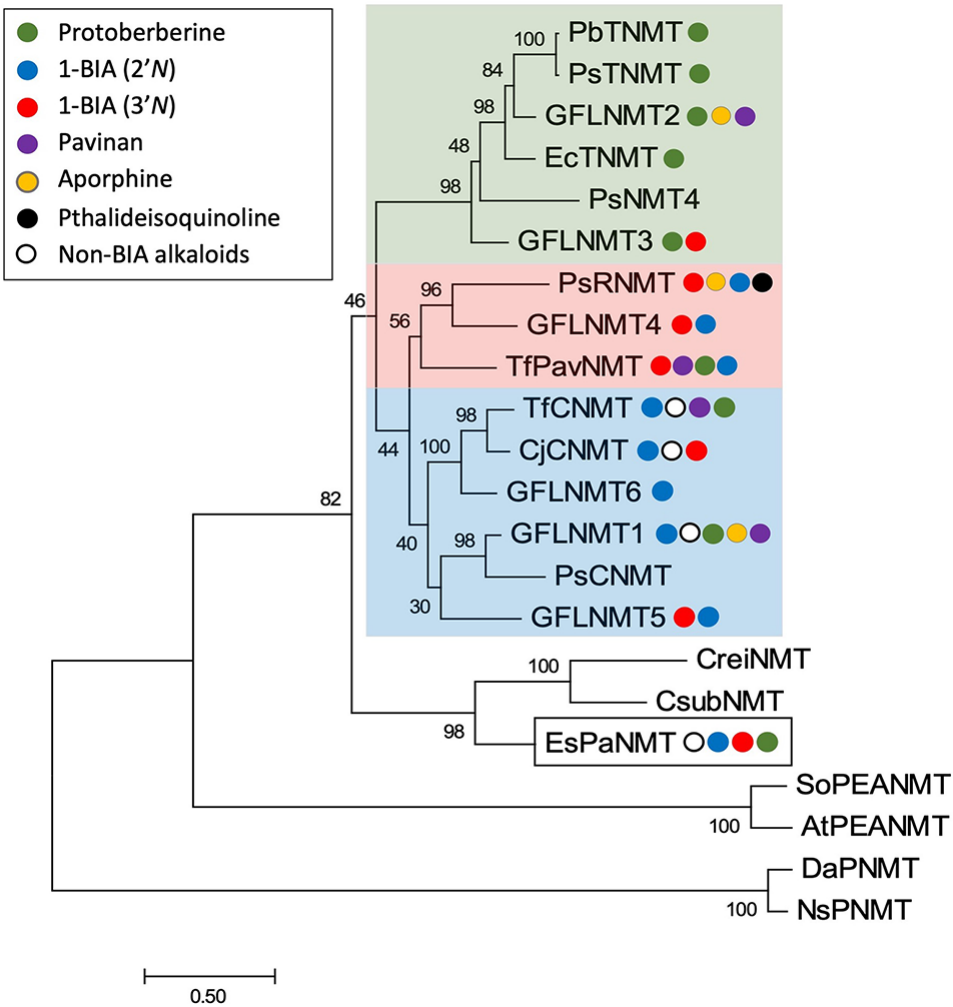
Rooted neighbor-joining phylogenetic tree of BIA *N*-methyltransferases labeled with reported *in vitro* substrate range. The optimal tree is drawn to scale with branch length in units of substitutions per site. Frequencies shown at each node represent the percentage of 500 bootstrapped replicate trees in which the associated taxa clustered together. The analysis involved 22 sequences and positions with less than 50% coverage were discarded, resulting in a final dataset with 358 positions. The tree was rooted with distantly related plant putrescine and phosphoethanolamine *N*-methyltransferase sequences. The analysis was conducted in MEGA7 (Kumar et al., 2016). BIA NMTs are labeled with colored circles representing their reported *in vitro* activities, in order from strongest to weakest (green, protoberberine *N*-methylation; blue, 2′ 1-BIA *N*-methylation; red, 3′ 1-BIA *N*-methylation; purple, pavinan *N*-methylation; yellow, aporphine *N*-methylation; black, pthalideisoquinoline *N*-methylation; white, *N*-methylation of other alkaloids including isoquinolines). Clades with recognizable majority activities are shaded with rectangles in the corresponding color. The BIA NMT-like *E. sinica* phenylalkylamine NMT (EsPaNMT) implicated in ephedrine biosynthesis is indicated with a white box. Genbank accession numbers for BIA NMTs are provided in **Supplementary Table 1**. *Datura stramonium* putrescine NMT (DaPNMT; CAE47481); *Nicotiana sylvestris* putrescine NMT (NsPNMT; BAA74544); *Arabidopsis thaliana* phosphoethanolamine NMT (AtPEANMT; NP_188427); *Spinacea oleracea* phosphoethanolamine NMT (SoPEANMT; Q9M571). *Coccomyxa subellipsoidea* NMT (CsubNMT; XP_005645141); *Chlamydomonas reinhardtii* NMT (CreiNMT; XP_001695187).

Phylogenetic analyses carried out in conjunction with the isolation of new BIA NMTs have generally agreed upon the existence of several clades roughly corresponding to subtypes (i.e., CNMT-, RNMT-, or TNMT-like; **Figure 6**) (Liscombe and Facchini, 2007; Liscombe et al., 2009; Morris and Facchini, 2016). However, the detailed topology of BIA NMT gene trees has varied substantially from one report to the next and all should be interpreted with caution. Recently, an analysis of more than 90 putative BIA NMTs from Ranunculales recovered four well-supported clades, three of which correspond to the known BIA NMTs and were experimentally validated as loosely predicting function (Hagel et al., 2015). Superimposition of reported *in vitro* activities over a BIA NMT phylogeny clarifies this idea (**Figure 7**). In particular, a well-supported monophyletic group of enzymes shown to almost exclusively catalyze *N*-methylation of protoberberine substrates is evident. However, enzymes with weak TNMT-like activities are present in other clades and may have evolved this function independently. Enzymes primarily accepting BIAs with tertiary nitrogen atoms other than protoberberines (i.e., RNMT-like) also form a single clade. Notably, members of this clade accept BIAs with a broad range of carbon skeletons, which includes aporphines, pavinans, and pthalideisoquinolines. On the other hand, the cluster of CNMT-like enzymes that preferentially target 1-BIA substrates with secondary nitrogen atoms are reported to have a more restricted substrate range. Although not evident in the phylogenetic analysis presented here, other reports have consistently indicated that CNMT enzymes are more ancestral. Later evolution of the RNMTs and TNMTs is consistent with the cumulative hypothesis, in which enzymes operating further downstream in biosynthetic pathways are recruited later (Granick, 1957). Conclusive statements regarding the evolutionary history of this enzyme family await careful phylogenetic study, ideally including sequences obtained from many species beyond those typically used as model systems for BIA biosynthesis and supported by functional characterization of resurrected ancestral enzymes.

Intriguingly, BLAST searches of publicly available nucleotide sequence databases (NCBI NR, OneKP) reveal that transcripts encoding BIA NMT-like proteins (40–70% amino acid identity; **Supplementary Data Sheet1**) are present in a wide range of flowering plants as well as algae, mosses, gymnosperms, and gnetophytes (Matasci et al., 2014). To the best of our knowledge, all but one of the cloned and functionally characterized members of this large NMT family belong to the Ranunculales order and are implicated in BIA biosynthesis. Given their apparently ancient origin and widespread occurrence, including in species not known to produce alkaloids of any sort, the functional significance and maintenance of BIA NMT-like genes through many millions of years of plant evolution is a fascinating mystery.

Several pieces of evidence point to the possibility of a relatively ancient origin for BIA biosynthesis, which likely included the activity of NMTs. The sporadic but widespread distribution of BIA biosynthesis in eudicots, along with detection of the “gateway” norcoclaurine synthase (NCS) activity in a broad range of plants, supports a proposal that the evolution of BIA biosynthesis may have a monophyletic history in angiosperms (Liscombe et al., 2005). In fact, the occurrence of several BIAs in *Gnetum* species (e.g., 8-benzylberbine), and of NCS activity in *Ephedra distachya*, further suggests that the evolutionary origin of BIA biosynthesis may have been at least as ancient as the divergence of the Gnetophytes (Xu and Lin, 1999; Rochfort et al., 2005; Martin et al., 2011). Although no BIA biosynthetic studies have been completed for these plants, in particular, the production of similar BIAs in other species is known to require a CNMT (Hagel and Facchini, 2013). Interestingly, a recent investigation of *Ephedra sinica* identified a BIA NMT-like enzyme (Phenylalkylamine NMT; EsPaNMT) implicated in the biosynthesis of ephedrine, which also has promiscuous activity on several other alkaloids including 1-BIAs (Morris et al., 2018). This observation supports the notion that the ancestor to all extant BIA NMT-like enzymes may have had a very broad range of activities and which was refined and subfunctionalized in certain lineages, where BIA biosynthesis provided a selective advantage. Of course, an alternative hypothesis is that EsPaNMT and the BIA NMTs were recruited independently from a functionally distinct ancestral lineage and simply converged on BIA NMT function. In any case, maintenance of BIA NMT-like genes in plants over evolutionary timescales implies that they must function in some useful role. It will be interesting to discover in the coming years whether the annotation of this family as BIA NMTs is simply a historical accident or an accurate representation of their broader roles in plants.

## Forces and Mechanisms Shaping BIA Methyltransferase Evolution

As for most specialized metabolites, the forces driving the evolution and maintenance of BIA biosynthesis are not yet fully understood (Weng, 2014). Generally, BIAs are assumed to provide defensive advantages *via* antiherbivore and antimicrobial properties (Hagel and Facchini, 2013). In the case of cultivated BIA-producing varieties, such as *P. somniferum*, artificial selection for the presence of psychoactive morphinans may also have played a minor role in recent times. However, potent biological activities are only firmly demonstrated for a small fraction of BIAs (e.g., berberine, sanguinarine, magnoflorine, morphine), and thus, straightforward adaptive evolution does not comfortably explain the tremendous chemical diversity observed across BIA-producing species and individuals. When attempting to justify the presence of apparently useless, yet metabolically costly, BIAs in a given plant, it is important to appreciate that the biochemical snapshot we obtain in the present day results from a complex evolutionary history spanning innumerable shifts in herbivore and pathogen challenges. Accordingly, biosynthesis of some functionless BIAs may have resulted from pressures no longer present in the environment. Alternatively, it has also been proposed that BIA metabolic diversity may be a useful trait in its own right (Facchini et al., 2004). That is, the production of a large and dynamic repertoire of potential defense molecules, which varies from individual to individual, may represent a form of “diversified bet hedging”, which can ensure the survival of at least some members when a lineage is suddenly faced with novel challenges. The canonical example of this type of coping strategy is seed germination timing, but variation in BIA profile could also conceivably fit the theoretical criteria (i.e., improved long-term evolutionary success despite reduced mean fitness, *via* a reduction in detrimental temporal fitness variance) (Childs et al., 2010). As reviewed in the preceding sections, the BIA MTs show substantial promiscuity and, perhaps more than any other class of enzyme, greatly expand and diversify the pool of BIAs that are produced.

Duplication followed by sub- or neo-functionalization is thought to be a crucial mechanism underlying the diversification of most eukaryotic gene families, and this also applies to the BIA MTs (Taylor and Raes, 2004). In *P. somniferum*, Graham and colleagues identified a genomic region on which many genes required for noscapine biosynthesis were clustered, including three BIA OMT genes corresponding to PsSOMT2, PsSOMT3, and PsSOMT1, various other BIA pathway genes and many transposable elements (Winzer et al., 2012). Based on sequence homology and intron–exon structure, these OMT genes were suggested to have arisen *via* tandem gene duplication, potentially followed by transposon-mediated cluster rearrangement. More recently, a *P. somniferum* whole genome was reported, which provides further support for a history of MT gene duplication (Guo et al., 2018). The authors described a relatively recent whole genome duplication as well as more ancient segmental duplications likely to have resulted in new BIA MT gene copies. Aside from MTs in the noscapine cluster reported previously, at least seven additional MT genes are present in their assembly (NCBI BioProject PRJNA435796). Notably, two copies of genes encoding Ps6OMT tightly linked to PsCYP80B1 (*N*-methylcoclaurine 3′-hydroxylase) exist on two separate contigs, suggesting the occurrence of either dispersed duplication or tandem duplication followed by genomic rearrangement. In addition, two copies of genes encoding PsN7OMT are identifiable. Given that both 6OMT and N7OMT are necessary for the biosynthesis of papaverine (**Figure 2**), which is particularly abundant in *P. somniferum*, it appears that increasing gene dosage is one important mechanism enhancing the contribution of key MTs to BIA biosynthesis. In contrast to the OMTs, only single functional copies of genes encoding PsCNMT, PsTNMT, and PsRNMT are evident in the genome. Although not tightly linked, *CNMT* and *TNMT* are located in the same region (roughly 10 MBp apart) of one chromosome. Aside from sub- and neo-functionalization, duplicated genes may often become inactive or pseudogenized. For example, three pseudogene copies of *TNMT*, within ∼30 kb of each other, are reportedly linked to the noscapine cluster (Winzer et al., 2012). In addition, examination of the *P. somniferum* genome suggests that such a fate is quite common for duplicated BIA MTs. BLAST searches of the published assembly reveal many putative pseudogenes, corresponding in particular to *4′OMT2*, *TNMT*, and *RNMT*. These are generally found in tight clusters indicative of tandem duplication. Although outside the scope of this review, it is clear that linkage of MT genes with those encoding upstream and downstream enzymes is an important contributor to the biosynthesis of BIAs. In addition to making co-inheritance of a useful group of alleles more likely, clustering probably facilitates coordinated gene expression *via* chromatin remodeling. Given the substantial amount of clustering evident in the *P. somniferum* genome, it will be interesting to discover whether similar structures exist in other BIA-producing species and, if so, whether clustering is an ancestral feature or yet another example of convergence.

## Future Directions

In the preceding sections, we reviewed the wealth of information presently available concerning BIA MT structure, function, and relationship with host plant chemodiversity. Although many important insights have been obtained in recent years, much remains to be done if the sea of information is to yield more widely applicable knowledge and conceptual understanding useful to the field of plant biochemistry as a whole.

In spite of the fact that the role of MTs in the central BIA pathway (i.e., leading to core intermediate (*S*)-reticuline) is firmly established in model species such as *P. somniferum* and other members of Ranunculales, it would be worthwhile to verify that this knowledge is applicable in more distantly related BIA producing plants such as the Piperales, Cornales, Laurales, Sapindales, and Proteales (Shulgin and Perry, 2002; Liscombe et al., 2005). Similarly, the role of MTs in the many “unusual” BIA branch pathways has not been investigated. This includes pathways biosynthesizing the rhoeadine alkaloids present in *P. rheas* (Rönsch, 1986), benzylprotoberberines (e.g., Latifolian A) occurring in *Gnetum latifolium* (Rochfort et al., 2005), hexahydrobenzophenanthridines (e.g., Corygaline A) occurring in *Corydalis bungeana* (Gao et al., 2018), as well as the dimeric and trimeric BIAs reported in many species (Schiff, 1991). Biosynthesis of aporphine alkaloids in *Nelumbo nucifera* (Proteales) has recently received some attention; however, most analyses assumed that central BIA biosynthesis is the same as in Ranunculales and, furthermore, that *N. nucifera* OMT and NMT homologs catalyze the same reactions as reported in other species (Menéndez-Perdomo and Facchini, 2018). Going forward, it would be valuable to carry out in these species the same types of studies as were used to firmly establish the routes of biosynthesis in Ranunculales (e.g., labeled tracer feeding, detection of intermediates, and activities). Furthermore, it is crucial to experimentally validate the function of putative BIA MTs when they are discovered. Whereas heterologous expression and* in vitro* assays can readily be applied to proteins originating from these other species, *in planta* approaches (e.g., virus-induced gene silencing, CRISPR-mediated knockout) have been used primarily in *P. somniferum*, and substantial method development may be required before these tools can be brought to bear in a wider context. Taken together, the above experiments would shed light on the question of whether BIA biosynthesis truly is monophyletic and as widely conserved as generally assumed or whether different enzymes and pathways have evolved to converge on BIA biosynthesis by different means.

Despite recent publications that have improved the situation, structures of BIA OMTs accepting a full range of BIA scaffolds (e.g., pthalideisoquinolines) or displaying alternate regio-specificity (e.g., 4′OMT) are missing from the literature. Comparison of these with existing structures would suggest how OMTs discriminate between highly similar molecules, and targeted mutagenesis would then allow for experimental validation of these hypotheses. Alternatively, a focused analysis of multiple functionally analogous BIA OMTs from distinct phylogenetic lineages (e.g., Tf6OMT, Ct6/7OMT, Cc6OMT1) would reveal to what extent the mechanisms of substrate binding and catalysis are conserved. Although these types of comparative studies have been limited by the recalcitrance of plant enzymes to crystallization, modern protein engineering methods such as Surface Entropy Reduction can help overcome these challenges (Cooper et al., 2007; Cabry et al., 2019). From a biotechnological standpoint, crystal structures or docking studies with a wider range of inhibitor molecules (e.g., pathway intermediates or end products) would be valuable in pointing the way to engineering feedback-insensitive variants desirable for industrial applications. Although BIA NMTs are relatively well covered in terms of available structures, certain features are still mysterious. Catalysis is still not fully understood, and this issue is compounded by difficulties in obtaining crystal structures with the enzyme’s “true” substrate bound. One promising approach to this problem involves the use of a reactive SAM analog (*S*-adensoyl-vinthionine) to form a bisubstrate adduct *in situ*, which remains trapped in the active site of the crystallized NMT (Qu et al., 2016). Another open question, potentially explored *via* biophysical modeling, is how these enzymes’ binding pockets successfully discriminate between rather similar BIAs despite seemingly forming very few specific interactions. Similarly, the unique *N*-terminal extension and active site “gate”, which might contribute to substrate selectivity in BIA NMTs, await careful study. In these cases, domain swap or deletion experiments should yield useful information on their function.

Of particular interest is the biochemical and physiological significance of MT dimerization. Structural elucidation of both functional and non-functional heterodimers (e.g., PsSOMT2:PsSOMT3 vs PsSOMT2:PsN7OMT) might reveal the subtly different interactions, which prevent or allow catalysis on certain substrates. However, targeted mutagenesis will undoubtedly be required to verify such hypotheses. Although only one heterodimer is known to be physiologically relevant at present, this is likely to change with more study. Combinatorial expression of BIA MTs in heterologous systems containing reconstituted BIA pathways is a powerful system with which to search for such interactions. However, it will also be crucial to validate that these heterodimers form in plants and make meaningful contributions to biosynthetic capacity. Ideally, this will be done with a combination of *ex vivo* (e.g., pull down, enzyme assay) and *in vivo* (e.g., FRET, gene knockout) methods. Of course, while considering the occurrence of heterodimerization, it will be crucial to also consider higher-order interactions with other proteins and enzymes that might form BIA metabolons.

Recent interest in understanding BIA biosynthesis in a wider range of plants should soon provide a more diverse set of BIA MTs to study. Modern computational resources and algorithms should allow for robust analysis of all these sequences, resulting in reliable phylogenies clarifying their interrelationships. As DNA synthesis costs continue to decrease, resurrection of ancestral enzymes should become routine and will allow us to answer long-standing questions about MT evolution. For example, it will be fascinating to discover what functions the ancestral class II OMT may have had and what trajectories lead to the extant functional diversity. Similarly, it should be possible to test the long-standing hypothesis that extant BIA NMTs diverged from a CNMT-like ancestor, and whether neo-functionalization or, rather, sub-functionalization then came into play. Along with the analysis of transcripts and encoded enzymes, genome structure will undoubtedly contribute to understanding the mechanics of MT evolution. Although the recently published *P. somniferum* genome has begun to shed such light, the significance of certain features (e.g., clustering) would be more evident if the genomes of additional *Papaver* species, more distantly related BIA producers, and closely related non-producers were available. Comparative genomics should reveal the timing of gene duplications and suggest how selection and drift contributed to the present complement of BIA MTs.

Ultimately, a complete understanding of the determinants of BIA MT function and the evolutionary trajectories that led to the formation of specific enzymes will reveal an important part of how the exquisite BIA biosynthetic pathways came to be. In combination with existing knowledge regarding caffeine biosynthesis and, eventually, with knowledge concerning the many other alkaloid pathways, these discoveries will allow us to reach satisfactory answers to the long-standing questions of how and why plant-specialized metabolism achieves such tremendous diversity.

## Author Contributions

JM wrote the manuscript and created the figures. PF edited the final draft of the manuscript.

## Funding

JM is the recipient of a Natural Sciences and Engineering Research Council of Canada Postgraduate Scholarship. This work was funded by a Natural Sciences and Engineering Research Council of Canada Discovery Grant to PF.

## Conflict of Interest Statement

The authors declare that the research was conducted in the absence of any commercial or financial relationships that could be construed as a potential conflict of interest.

## Supplementary Material

Supplementary Datasheet 1 A Selection of BIA NMT-like proteins sharing 40-70% amino acid sequence idenetity.
