# Corrigendum: Molecular Origins of Functional Diversity in Benzylisoquinoline Alkaloid Methyltransferases

**DOI:** 10.3389/fpls.2019.01436

**Published:** 2019-11-08

**Authors:** Jeremy S. Morris, Peter J. Facchini

**Affiliations:** Department of Biological Sciences, University of Calgary, Calgary, AB, Canada

**Keywords:** Benzylisoquinoline, Alkaloid, Methyltransferase, specialized metabolism, molecular evolution

In the original article, the author names were ordered incorrectly as “Peter J. Facchini and Jeremy S. Morris”. The correct order is “Jeremy S. Morris and Peter J. Facchini.”

The authors apologize for this error and state that this does not change the scientific conclusions of the article in any way. The original article has been updated.

