# Supplementary material for: Molecular Origins of Functional Diversity in Benzylisoquinoline Alkaloid Methyltransferases
Source: Front Plant Sci. 2019 Aug 30;10:1058. doi: 10.3389/fpls.2019.01058 (PMC6730481; doi:10.3389/fpls.2019.01058)
Supplement: Supplementary Datasheet 1 — A Selection of BIA NMT-like proteins sharing 40-70% amino acid sequence idenetity. [file DataSheet_1.pdf]

>CsubNMT_XM_005645084.1_-_ORF_1_(frame_1)
MQAVERDLVPDFLIRRGIRYLLSQRVQNAREGGVEEQERRKQAFVDELKSLPIAVQTSIANEQHYEAKYFLLVLGKHLKY
SSCLYPSSSSTLDEAEEAMLGLCCERAQLADGQTILELGCGWGSMCLYMAGKFPNSTITAVSNSSTQKALIDERAKERGL
SNLTVITADVVEFDTKQKFDRVVSIEMFEHMKNYQALLKNISKWLKPGGHLFVHIFTHKTLAYHFEVKGEDDWMAKYFFT
GGTMPSADLLHHFQDDLALQQQWAVNGTHYSRTLEDWLRRQDRQRAQILPIMKKTYGDDQGLRWWVYWRLFYLACSELFN
YNGGEEWFVSHYLFKKH
>CreiNMT_XM_001695135.1_-_ORF_1_(frame_1)
MPIAVNTSEANEQHYEIPTPYYLLVLGSHLKYSSCLYRSPRESLEQAEYNMLDLYCERAQLRPGQRVLELGCGWGSFSLF
AAARYPASTFFAVSNSATQKAFIDGEAAKRGITNLTVLTANMVDFEAPTANGLFDRVVSIEMFEHMKNYQRLLRKVSGWL
AAEGKLFVHIFLHRTTPYHFEVQSEEDWMSKYFFTGGTMPSSDLLLYFQDDLVIRNHWYVNGRHYSRTLEDWLVRHDRNS
REVRKILTTAYTGSKDSANVWFHRWRIFYIACRRLFNYKGGEEWGVGHFLFEQRG
>PpatNMT_XM_024544070.1_-_ORF_1_(frame_3)
MASLIKAGRDATLKVSMSALQMNVIPDMVLRFGVRKLLEGRLSEITKSTLEAQMQELLSFVQSLKQMPIAVHMEDANTQH
YEVPTEFYHLVLGKRLKYSSAYFPKKDSTLDEAEESMFALYAERAQLQDGQSVLDVGCGWGSLSMYIAEKFPNSNVTGVS
NSETQRAFITEECRKRGLSNVTIITCDMNVFNIDKSFDRILSIEMFEHMKNYEKLLKKISSWLKPDGLLFIHIFVHKSIP
YHFEDKGEDDWMSRNFFTGGTMPSSSLLLYFQDDVSIVNQWFVNGTHYARTSEAWLLKMDAQVKVIRPIFEKTYGEGEAT
KWIANWRTFFIAVAEMFAFRNGEEWGVCHYLFKKK
>SlesNMT_onekp_GOWD_scaffold_2083338
MDFLKSSSYDAVLRLGIRALEANVLPDFVLRRATRALLKSRLNSLNKSTGEEQLQALMSFVQSLKEMPIAVNTESANSQH
YEVPTEFYQLVLGKRLKYSSSYFPNTSSSLDEAEEAMMALYSERSQLQDGQEVLDLGCGWGSLSIYLAEHYPNSKITSVS
NSETQRAFITEECRKRGLSNVTVITCDINTFNIDKTFDRILSIEMFEHMKNYQKLLRNISSWLKTDGLLFIHIFAHRSVP
YHFEDEGEEDWMSRTFFTGGTMASASLLLYFQDDVSILNQWYVNGKHYAQTSEEWLKKLDRELRTIRPIFAKAYGEADVN
KWIANWRTFFIAVAELFGYNNGEEWGVSHYLFKKK
>SmoeNMT_XM_024689855.1_-_ORF_1_(frame_3)
MAPNARPSKGFLYGSVLRAGLSAMEANLVPDFVLRRATRMLLASRLRELRRHSVEEELNELMKFAKSLKQMPIAIHTDAA
NSQHYQVPTEFYKLVLGKHLKYSCAYFCKDSYTLDQAEEAMLELYCERAQLKDGQVVLDLGCGWGSLSLYIAEKYPASRI
TGVSNSATQREFIEGECSKRGLSNVKVIVSDINDFQAPEKFDRILSIEMFEHMKNYQKLLKNIASWMKPDCLLFVHIFCH
KNHAYHFEDTGDDDWMARSFFTGGTMPADPLLLYFQEDVSILHHWRLSGTHYARTSEAWLKRMDQNLSSIRPIFAKAYGE
SDASTKWLAFWRTFFIAVAELFAFDNGQQWIVSHYLYKLKASDMDANAT
>EgigNMT_Locus_13525_Transcript_1
MEFVKKKSYEATLKIGMGALEANLLPDFMIRRGTRLLLASRLQSLAIPSAEDQLRNLLDFVHSLKKLPIAIHTSAANSQH
YEVPTEFYKIVLGKHLKYSCALFTGKSCTLEEAEVAMLELYCERAQLKDGHHVLDVGCGWGSLSLYIAEKFPNSTITSVS
NSATQKKFIDDECKRRGISNITVVTCDINSFDAERTYDRIFSIEMFEHMKNYHKLLSKISSWMNPEGLLFIHIFCHKQYA
YHFEDSGEDDWMARYFFTGGTMPADRLLLYFQDDVSIVNHWRVNGKHYSQTSEEWLKRMDANEKFLRPILQKTYGNESEK
WWAYWRTFFIAVAELFSYNDGEEWMVSHYLFKRKL
>GbilNMT_Gb_29392
MEGIMQVSYDMAIRGTMRSLEANLLPDFVIRKLTRMLLASRLRLGYKPNAESQLSDLIAFANSLKQMPIALNTETPKAQH
YELPTSFFQFVLGKHLKYSAAYFSRNTSTLDEAEEAMLELYCKRAHIKNGQTILDLGCGWGSLTLYIARKYADNHVTGIC
NSTTQKAFIEEQCREQGLSNVLVIVADISNFNMERTFDRIISIEMFEHMKNYQSLLKKISLWMNQESLLFIHYFCHRIFA
YHFEDVHEDDWITRYFFTGGTMPSKSLLLYFQDNITVVDHWVVNGKHYARTSEEWLKNMTKNLPSIMPILQDTYGEESKK
WMVYWRTFFIAVTELFGYNDGEEWMVSHFLFKRK
>EsinPaNMT_MH029305.1
MEEAKMATLGGASYAMIVKTMMRSLEANLIPDFVLRRLTRILLASRLKLGYKQTAELQLADLMSFVASLKTMPIALCTEE
AKGQHYELPTSFFKLVLGKHLKYSSAYFSEHTRTLDEAEEAMLALYCERAKIEDGQKILDIGCGWGSFSLYVAERYPKCE
ITGLCNSSTQKAFIEQQCSERRLCNVTIYADDISTFDTESTYDRIISIEMFEHMKNYSTLLKKISKWMNQECLLFVHYFC
HKTFAYHFEDVDEDDWMARYFFTGGTMPASSLLLYFQDDVSVVDHWLINGKHYAQTSEEWLKRMDHNLSSILPIFNETYG
ENAAKKWLAYWRTFFIAVAELFKYNDGEEWMVSHFLFKKK
>GmonNMT_TnS000946403t08
MENHATRGWLDASYRVTARTVMRFLDCNLLPDVVTRRLTRILLASRLKSVSNPSAHLQLSELMAFVDYLKQMPIAICTEE
AKGQHYELPTSFFELVLGKHLKYSCAYFSSPTTTLDEAEVAMLALYCERAKIEDGQKILDVGCGWGSLALYIAERYPKCH
VTGLCNSATQKKSIKEKCSERGFANVDIYADDISNFKLEETFDRIISIEMFEHMKNYEVLLKKISLWMHSESLLFVHHFC
HKTYAYNYEDVDEDDWMARYFFSGGTMLSSNLLLYFQDDVSVINHWLVNGMHYAQTSEEWLKKMDKNIVSLVSIFKETYG
ENDAKKWLAYWRTFFIAVAELFGYNNGEEWMVSHLLFKKKK
>WmirNMT_m.9074_doi_10.1093/gbe/evv171

MESVGLARRVMNASYGLAAKAVLRLLEGNVIPDFLIRRLTRLLLAARLKECLQPTADLQLSQLMTFVQALKQMPIAVSTE
AAKVQHYELPTSFFKLVLGQRLKYSSAYFSSHVTTLEEAEEAMLALYCERAEVKDGLTILDLGCGWGSLILYIAEKYPKC
EVTGVCNSVTQMKIIEEQCSERGLSNIKIYANDVCNIELKTTFDRVLSIEMFEHMKNYGNLLEKISQWMNNESLLFVHHF
CHKSFAYHFEDTGEDDWITRYFFTGGTMPSSNLLLYFQDNVSIVNHWLINGKHYAQTSEEWLKRMDKNLSSILPIFNDTY
GENECKKWLGYWRTFFISVAELFSYNDGEEWMITHLLFRKRS
>CjapNMT_AK407524.1_-_ORF_1_(frame_2)_1
MEVQVQRGIMQHPYDVLVKGVMRVLEANLLPDFLLRRITRTLLARSLHLGYKPNTQLQLQDLMAFINSLKRMPIALSTEK
PKAQHYELPTSFFKLVLGKNLKYSSCYFASDTNTLDEAEESMLSLYCERAQIKDGQTILDIGCGWGSLTLYMAKKYPNSH
VTGICNSTTQKEYIIEKSSELGMSNTVIYAGDITSFKMESTFDRIISIEMFEHMKNYGKLLKKISLWMSQESLLFIHHFC
HRVFAYHFEDINEDDWMARYFFTGGTMPAASLLLFFQEHVSVIEHWLVNGKHYARTSEEWLKNMDKNMKFIYPILIDTYS
VDSAKKWMVYWRTFFIAVAELFGYNDGDEWMVSHFLFKEK
>PglaNMT_BT112259.1_-_ORF_1_(frame_2)
MMETQQPGGIMNAAYNVSVRVFLRWLEANLVPDYIIRKLTRTLLAKRLRLGYNLSACLQLADLMAFINALKQMPIAMNSE
AAKAQHYELPTSFFKLVLGKHLKYSCAYFTRSTSTLNEAEEAMLELYCERAKVEDGQTVLDIGCGWGSLTLYIARKYPNN
QVTGICNSTTQKVFIEEQCSEMGLCNVIIFADDISKFEMENTFDRIISIEMFEHMKNYGSLLKKISHWMKPESLLFVHQF
CHKVFAFHFEDVHEDDWITRYFFSGGTMPSSNLLLYFQDHVAVVDHWLVNGKQYAQTSEEWLKNMDKNLTSIIPIFNDTY
GESSTKTWIAYWRTFFIAVEEFFGYNDGEEWMVSHFLFKKK
>PparNMT_onekp_IIOL_scaffold_2076448 Pinus_parviflora
MMETQQPRGMMNVAYNVSVRVFMRWLEANLVPDYIIRKLTRAFLARRLRLGYKLNASLQVADLMAFINALKQLPIAMNTE
AAKTQHYELPTSFFKLVLGKHLKYSCAYFTSSTSTLDEAEEAMLELYCERAQVKDGQTILDIGCGWGSLILYIARKYPNN
QVTGICNSKTQKLFIEEQCSEMGLCNVVIFADDISKFEMENTFDRVISIEMFEHMKNYGSLLRKISQWMKLESLLFVHHF
CHKVFAFHFEDIDEDDWITRYFFAGGTMPSSNLLLYFQDHVSVVDHWLVNGKHYAQTSEEWLKNMDKNLTSIIPIFNDTY
GENSAQKLMAYWRTFFIAVVELFGYNDGEEWMVSHLLFKKK
>AtriNMT_XM_006843096.3_-_ORF_1_(frame_2)
MIDVLVQVPYEATVRTVLGALERNIVPDLLVRKLTRLLLASRLRSGYKPTSEAQLSQLLQFIHSLKEMPIAVEMGKAKDQ
HYELPTSFFKLVLGKNLKYSCCYFKNKYITLEEAETAMLELYCERAQIKDGHSVLDVGCGWGSLSLYIAQKYSNCHVTGI
CNSTTQKEFIEEQCRERKLSNVEIIATDISNFEMDATFDRIMSIEMFEHMKNYQALLRKISKWMKPDGLLFIHYFCHKTF
AYHFEDKNDDDWITRYFFTGGTMPSANLLLYFQDDVSIVDHWLVNGKHYAQTSEEWLKRMDNTSASIRPIMEATYGKDAA
TKWTAYWRTFFISVAELFGYNDGEEWMVSLFLFKRK
>CwinNMT_onekp_IFCJ_scaffold_2021702
MTGEAEQAKKEDLLKRWEEGQVPDEELRRLMRIQISRRLQWGYKPTYEQQLAENIHLAKALRQMNVATETDTLDSQMYEI
PISFLKLTFGKTLKESCCYFKDESMTLDEAEIAMLDLYCERAQVKDGERILDLGCGQGALTLHVAQKYENCHVTAITSSI
LQKDFIEEQCKKLKLENVEVILADISKFEMDAIYDRIFVVGLFEHMQNYELLLKKISKWMTENGSLFVDHLCQKTFAYQY
KPLDEDDWYTNFIFSSGTLILPSASFLLYFQGDVSVVDHWTLSGKHFSRTNEEWLKKLDANKDAAKEIAESFSGSKEAAV
KWINHWRGLCMSAAELFGYRNGEEWMSSHILLKKK
>DwinNMT_onekp_WKSU_scaffold_2021598
MGALTQVPYEATVRVMLASLERNLLPDVVIRKLTRLLLAYRLRSGYNPSSHLQLSNLLHFAHSLEHMPIAVETDKPKSQH
YELPTSFFKIVLGKNLKYSCCYFPDKSNTLDDAENAMLELYCERAQIKDGQTVFDVGCGWGSLSLYIAKKYSNCSITGIC
NSITQKEFIEEQCREFKLSNVEIIVADISNFEMQASFDRILSIEMFEHMKNYRDLLKKISKWMKPDSLLFIHHFCHKAFA
YHFEDTNEDDWITRYFFTGGTMPSANLLLYFQDDVSVVNHWLVNGMHYAQTSEEWLKRMDQNMASIKPIMESTYGKDSAT
KWVAYWRTFFISVAELFGYNGGEEWMVTHFLFKKK
>PbolNMT_onekp_KRJP_scaffold_2097675
MAVDSKQPKKASVVALQRRLDEGSVPDVELKQLMRSEIARRLQWGYKPTYDQQIAQVVNFAKSLRHMSIATEVETLDDQM
YEIPVSFLKIMFGSKIKGSCCYFKDESMTLDDAETAMLDLYCERAQIEDGQSVLDLGCGQGALTLHVAQKYKKCRVTGIT
NSVSQKEFIEEESKKSGLSNVEIILADITKLETDATYDRILVIELFEHMKNYELLLRKISKWMTADGLLFVEHICHKTFA
YHYEPLDEDDWYTEYVFPAGTMIIPSASFLLYFQDDVSVVDHWTLSGKHYSRTNEEWLKRVDANIDAVKEIMESFSGSKE
EAAKWLKYWRGFCISGGELFGYNNGEEWMASHVLFRKK
>CfilNMT_onekp_VYLQ_scaffold_2009685
MAIDSTKQQQPAQRKASVLELQRRLDEGTVPDEELRPLMRSEIERRIRWATKPTHEQQVEQILSFAKSLRQMNIAVEVDA
LDDQMYEVPISFLKIMFGSKIKGSCCYFEDESTTLDQAEIAMLDLYCERAQIKDGQRVLDLGCGQGALTLHVAEKYKKCH
VTAITNSLSQKEYIEEESKKLHLSNVEVILADITKLNTIVTYDRILVIELIEHMKNYELLLRKISTWMAPDGLLFIEHIC
HKTFAYHYEPIDEDDWYTEYVFPAGTMIIPSASFLLYFQDDISVVDHWTLSGKHYSRTNEEWLKRVDLNIDKVREIMESF
SGSKEAASKWIKYWRGFCISGGELFGYNNGEEWMASHILFKKK
>GameNMT_onekp_BSVG_scaffold_2077304

```
MAQVSSKPVQSQGVMELMKRIEEGSVSDEEIRSLMKTQIEKRLQWGYKPTFQEQLAYNLNFAHSLRKMDIAKEIGTLDEQ
LYEVPTSFMKLMLGKSLKQSCCYFKDEYTTLDEAEIAMLDLCCERAHITDGQSVLDLGCGQGCVTLHIAQKYKNCHVTAV
TNSRAQKDYVEDQCKILQLSNVKVVLADLTKLEMKDTFDRIIIISVFEHMKNYELLLKKISKWMGSDGLLYLEHLCHKLI
AYSYEPIDEEDWYTESTFPAGTLNMPSASFFLYFQDDVSVVDHWILSGKHFARTMEEFLKKLDKNKEAAKEILVASTGSE
EVAMKLINFWRGFCIREAEVCKYNDGEEWMLSQLLFKKK
>EbenNMT_onekp_DHPO_scaffold_2076898
MDALIQVPYAATVRLMLSLLERNLLPDALIRKLTKLLLAGRLRSEYQPSSHLQLSRLVFFAHSLQHMPIAVETDKPKYQH
YELPTSFFKLVLGKNLKYSCCYFADKENTLEDGEKLMLDLYCERAEIKDGQTVLDVGCGWGSLSLYIAQKYTHCSVTGIC
NSMTQKEYIEERSRGLQLSNVQILVADISKFEMQGSFDRILSIEMFEHMKNYKELLKKISTWMKEDGLLFIHYFCHKSFA
YHFEDTNEDDWITRYFFTGGTMPSANLLLYFQDDVSIVNHWLVNGKHYARTSEEWLKRMDKNLLSITPIMESTYGKESAT
KWTVYWRTFFISVAELFGFNDGEEWMVAHFLFKKK
>Mmau_onekp_XQWC_scaffold_2013411
MGEETKQPKKAAVEELLKRLDEGRVPDEELRRLMRFEIGRRLQWGYKPTHEEQVAQVLELTHSLRKMSIATEVDTLDSQM
YEIPISFLQIMFGSLIKGSCCYFKDDSTTLDEAEIAMLDLYCERAQIKDGQSVLDLGCGQGALTLHVAQKYQNCRVTAIT
NSASQKDFIEDQSKKLKLSNVEIILADITKYVTETTYDRILVIELFEHMKNYELLLKKIAKWMKQDGLLFIEHICHKTFA
YHYEPLDEDDWYTEYVFPAGTMIIPSSSFLLYFQDDVSVVNHWTXXEEWLKRVDANVDAVREIMESFAGSKEAAEKWIRY
WRGFCISGIELFGYNNGEEWMASHILFRKP
>Afim_NMTHQ343195.1_-_ORF_1_(frame_2)
MASEKLNKTEMLRRLEEGSVPDEEFRRLIRIELGRRLRWYCQKKPTYEEQTAEIVALVKALRQMGITGDQSDQLNSDLYD
MPMSFLKITFGKLLKESGSYFKDDSMTLDEAEEAMLDLYCERAQLKDGQKILDLGCGQGAFTLHAAQKYKKSHVTAVTNS
ATQKKYIEDQCQILELSNVEVLLEDITQLTMEATFDRIIVIGLLEHMKNYGLLLQNISQWMAPADSLLFIDHVCHKSYFY
QCEPLDEDDWFAEYFFPPGSFAMPSASFLLYFQDDVSIVDHWILSGKHFHRTAEEWVKQLDTNLEKGKEILESKYGSKEA
ALKAFNHWRGLCIFSSEIFGYNGGEEWMTSHLLFKKK
>AeleNMT_onekp_PAWA_scaffold_2003669
METEKVNKSEMLKRQXGSVPDDELRRLMRFELGRRFQWCQKPTYEEQTAEILNLVKSLQKQGICTESDSLNSEIYETPIS
FLKTIHGKLLKMSACYFKDDSMTLDEAEEAMLDLYCERAQLKDGQRILDLGCGQGAFTLHVAQKYKKSHVTAVTNSASQK
MYIEEQCQILGLSNVQIILEDIAKLTMDTTFDRIIVIGLFEHMKNYGLLLRHISQWMSTQDSLLFVDHVCHKSFAYQYEP
LDKDDWFTEYIFPPGSVIVPSASFLLYFQDDVCLVDHWVLSGKHFSRTAAEWLKRLDGNVEKGKEILGSTYGSREAVLKE
INYRRGFCIFANEFFGYNGGEEHMTSQLLLKKK
>ShenNMT_onekp_QDVW_scaffold_2001457
MGLDEKGSDEMRRAKKMELVKRLEEGMVGDEELKVLMRTEIERRLQWGYKPTNHQQLSHLLNFVKSLRQMNIAIETDTLD
SQMYEVPISFLKIMFGKTIKGSCCYFKDDSMTLDEAEIAMLDLYCERAQIRDGQRVLDLGCGQGALTFHVAQKYENCHVT
AVTNSQSQKDFIEEQSKIRKLPNVEVLLGDISTLEMDLTYDRILVIGLFEHMKNYELLLRKISRWMTHDGLLFVEHVCHK
TFAYHYEPLDEDDWYTEYIFPVGTLIMPSTSFLLYFQDDVSAVDHWTLSGKHFLRTKEEWLKRLDANVEAGRGIXNNGVL
LW
>PausNMT_onekp_BYQM_scaffold_2012734
MMRYPYDVAVRGGLAALERNIMPDAVTRRLTRLLIGGRLRLGYRSPSAGDLLSDMMEFVKGLEKMPIAVETELAKMQHYE
VPTSFFRLVLGSRLKYSCCYFHENTRTLEDAETAMLELYCKRAQVEDGQTVLDVGCGWGTLCLYIAQKYKNCEVTGICNS
TTQRDFIEEQCQALQLSNVNIIVADISKFEMEALFDRIISIEMFEHMKNYKVLLKKLSGWMKEDSLLFVHHFCHKSFAYH
FEDTNEDDWITRYFFTGGTMPSANMLLYFQDDVAVFDHWLVNGTHYARTSEEWLKRMDKNMSSIKPIVESTYGKDSATKW
TAYWRTFFISVAELFGYNNGEEWMVAHLLFKKK
>PstrNMT_onekp_MFIN_scaffold_2013023
MDTVMRVPYDAAVRAALALLEKNLLPDPVARRLTRLLLASRLRSGYEPSAEIQLSHLVHFARSLEEMPIAIETDKAKSQH
YELPTSFFKLVLGKNLKYSSCYFQDTSDTLEDAEDAMLELYCVKAQVKDGQSILDLGCGWGSLSLYIAKKFSSCQVTGIC
NSTTQKAHIDEQCRDLHLSNVNIIVADIGQLEMEGSFDRIISIEMFEHMKNYKELLKKISKWMKEDGLLFVHYFCHKTFA
YHYEDAGEDDWITRYFFTGGTMPSANLLLYFQDDVTVVNHWLINGKHYAQTSEEWLKRMDNHSASIRPILESTYGKDAAV
KWTAYWRTFFIAVAELFGFNGGEEWMVAHFLFRKK
>AoffNMT_XM_020394977.1_-_ORF_1_(frame_1)
MPIAIETDKAKTQHYELPTSFFKLVLGKQLKYSCCYFPDKSTTLDDAENAMLELYCERAQVKDGQSILDVGCGWGSLSVY
MAQKYKNCSITGICNSTTQKAFIEEQCRDLQLSNVKIIVADISKFEMEASFDRIISIEMFEHMKNYKVLLKKISMWMKQD
SLLFVHYFCHKVFAYHFEDINDDDWITRYFFSGGTMPSANLLLYFQNDVSVVNHWLVNGMHYSRTSEEWLKRMDQNAASI
RPIMESTYGKDSATKWTAYWRTFFISVAELFGYSNGEEWMVAHFLFKKN
>PequNMT_XM_020729417.1_-_ORF_1_(frame_1)
MATVVQIPYNAAVRAVLASLERNLLPDAVVRRLIRALLAARLRRGYLPSAHLQLSQLLHFKNALEEMPIAVEIDKAKSQH
YELPTVFFKLVLGKNLKYSCCYFEDSASTLEDAEVAMFDLYCERAQIEDGQSILDLGCGWGSLSLYLAKKYKNCKITGIC
```

NSKTQKAYIDEQCREHQLSNVEIIIADISKHEMDASFDRIISIEMFEHMKNYKALLNKISKWMRPESLLFVHHFCHKTFA
YHFEDINDDDWITRYFFSGGTMPSSNLLLYFQFGVFIIILKLGLSCSEEWLRRMDKEADAIRPIMEATYGKDSARKWSAY
WRTFFISVAELFAYNNGDEWMVANFLFKKK
>NvirNMT_onekp_XEUV_scaffold_2130103_Narcissus_viridiflorus__-_ORF_1_(frame_3)
MGKLVEVPYNATVSVMLASLERNLLPDLVIRRLTRVLLASRLRHGYLPSAELQLSNLIQFKQSLEDMPIAVETDKAKIQH
YELPTSFFELVLGKNLKYSCCYFNVKSNTLEDAENAMLELYCERAQVKDGQSILDVGCGWGSLSLYIARKYKSCNITGIC
NSTTQRAYIEEQCRDLHLSNVKIIVADISKFEMEASFDRVISIEMFEHMKNYKALLKKISTWMKQDSLLFVHYFCHKVFA
YHFEDINEDDWITRYFFSGGTMPSANLLLYFQDDVAVVDHWLVNGMHYSSTSEEWLKRMDQNRAAIRPIMESTYGKDSAT
KWTVYWRTFFIAVAELFAYNNGEEWMVAHYLFKKK
>PdacNMT_XM_008798273.2_-_ORF_1_(frame_3)
MEALVRMPYEAAVRMAVASLERNLLPDAVVRRLTRLLLASRLRSSYLPSADLQLARLLDFKCSLQDMPIAVETDKANSQH
YELPTSFFKLVLGKNLKYSCCYFQDKSSTLEDAENAMLELYCENAQVKDGQSILDLGCGWGSLSIYIAKKYQNCSITGIC
NSTTQKAYIEEQCRDLQLFNVEVIVADISKFEMLATFDRVISIEMFEHMKNYKELLKKISKWMKQDSLLFVHHFCHKTFP
YHFEDKNEDDWITRYFFAGGTMPSANLLLYFQDDVTVVNHWLVNGTHYARTSEEWLKRMDRSLASVRPIFETTYGNDSAT
KWIAYWRTFFLAVAELFGYNNGEEWMITLYLFKKK
>EguiNMT_XM_010916904.1_-_ORF_1_(frame_1)
MEALLGMPYEAAVRVALASLERNLLPDAVVRRLTRLLLACRLRSGYLPSADLQLAHLLDFKRSLEDMPIAVEADKANSQH
YELPTSFFKLVLGKNLKYSCCYFQDKSCTLEDAENAMLELYCENAQVKDGQSILDLGCGWGSLSIYIAKKYRNCSITGIC
NSTTQKACIEERCRDLQLFNVEIIVADISKFEMEATFDRVLSIEMFEHMKNYKELLKKISKWMKQDSLLFIHHFCHRTFP
YHFEDKNEDDWITRYFFSGGTMPSANLLLYFQDDVAVVNHWLVNGTHYARTSEEWLKRMDQSLASIRPIFETTYGNDSAT
KWIAYWRTFFIAVAELFGYNNGEEWMIALYLFKKK
>MacuNMT_XM_009410568.2_-_ORF_1_(frame_1)
MEAIIRVPYEAAVRAALAALERNLLPDAVVRRLTRLLLAGRLRLCYLPSSDLQLAQLLRFKQSLEDMPIAVETDKAKSQH
YELPTSFFKLVLGENLKYSCCYFKNITSTLEDAENAMLELYSERAQLKDGQKILDVGCGWGSFVIYIAKKYKNCSITGIC
NSSTQKTHIEEQCRHLQLSNVEIIVADITKFEMEASFDRVVSIEMFEHMKNYKMLLKKISIWMKQDSLLFIHHFCHKTFA
YHFEDKNEDDWITRYFFTGGTMPSANLLLYFQDDVAVLNHWLLNGTHYARTSEEWLKRMDSNLTSIRPTFEATYGKDSAT
KWIAYWRTFFISVAELFGYNNGDEWMVALFLFKKK
>TaesNMT_AK450165.1_-_ORF_1_(frame_2)
MAAAAVAARAYEAAARSALAALERNLLPDAVTRRLTRFLLAQRLRLGYLPSAPLQLQDLLLFAHSLEGMPIAIEMDTAKT
QHYELPTTFFKLVLGKNLKYSSCYFPDDSSTLEDAEVAMLELYCERAQLQDGQSILDVGCGWGSLSLYIAKKYRNCNITG
ICNSTTQKAFIEEQCRENELSNVEIIVADISMFEMERSFDRIISIEMFEHMKNYKALFKKISRWMKEDSLLFVHYFCHKT
FAYHFEDKNDDDWITRYFFTGGTMPSANLLLYFQEDVSVVNHWLVSGTHYARTSEEWLKRMDKNIATIRPIFEKTYGRES
ATKWIAYWRTFFISVAELFGYNNGDEWMVAHHLFRKK
>ZmayNMT_BT041143.1_-_ORF_1_(frame_2)
MVAASVAERAYEAATRSALVALERNLIPDAVTRRLTRLLLAQRLRQGYLPSAPLQLQQLLQFVHSLEEMPIAIETDKAKA
QHYELPTTFFKLVLGKNLKYSSCYFPGDSSTLEDAEVAMMDLYCERSKLQDGQSILDVGCGWGSLSLYIAKKYRNCSVTG
ICNSTTQKAFIEEQCRDNELSNIEIIVADISKFEMERSFDRIVSIEMFEHMKNYKSLLKKISRWMKEDGLLFVHLFCHKA
FPYHFEDKNDDDWITRYFFTGGTMPSANLLLYFQEDVSVVDHWLVSGTHYARTSEEWLKRMDKSITSIRLIFEETYGKES
TTKWIAYWRTFFISVAELFGYNNGDEWMVAHYLFRKK
>AcomNMT_XM_020233317.1_-_ORF_1_(frame_3)
MVVEGLLRPPYEAAVRLSLAALERRLLPDAVVRRLTRLLLAARLRLGYLPTADLQLAHXLLFKQSLEEMPIAVETDKAKS
QHYELPTSFFKLVLGKNLKYSCCYFQNNSSTLEDAEDAMLELYCERAQLRDGQTILDVGCGWGSLSLYIAKKYRNCTITG
ICNSITQKAFIEERCREHQLSNVEIIVADISKFQMEATFDRILSIEMFEHMKNYKELLKKISMWMKEDSLLFVHYFCHKT
FAYHFEDKNEDDWITRYFFTGGTMLSASLLLYFQDDVTVVNHWLVNGTHYARTSEEWLKRMDQNITSVRPIFESTYGKES
ATKWIAYWRTFFISVAELFRYNNGDEWMVALYLFKKK
>HvulNMT_AK357827.1_-_ORF_1_(frame_2)
MAAAAVAARAYEAAARSALAALERNLLPDAVTRRLTRLLLAQRLRLGYLPSAPLQLQDLLLFAHSLEGMPIAIETDTAKT
QHYELPTTFFKLVLGKNLKYSSCYFPDDSSTLEDAEVAMMELYCQRAQLQDGQSILDVGCGWGSLSVYIAKKYRNCNITG
ICNSTTQKAFIEEHCRENELSNVEIIVADISKFEMERSFDRIISIEMFEHMKNYKALLKKISRWMKEDSLLFVHYFCHKT
FAYHFEDKNDDDWITRYFFTGGTMPSANLLLYFQEDVSVVNHWLVSGTHYARTSEEWLKRMDRNIATIRPIFENTYGRES
ATKWIAYWRTFFISVAELFGYNNGDEWMVAHHLFRKK
>OsatNMT_XM_015787859.1_-_ORF_1_(frame_3)
MAMAARAAYLAATRAALAALERNALPDAVTRRLTRLLLAQRLRLGYLPSSSSSAPLHLHHLLLFAHALEEMPIAIETEKA
KDQHYELPTTFFKLVLGRNLKYSSCYFPDESSTLEDAEVAMLELYCERAQLQDGQTILDVGCGWGSLSLYIAKKYSKCSI
TGICNSTTQKAFIEEQCRENELSNVEIIVADISKFEMERSFDRIISIEMFEHMKNYKALLKKLSRWMKEDSLLFVHYFCH

KTFAYHFEDNNEDDWITRYFFTGGTMPSANLLLYFQDDVSIANHWLVSGTHYARTSEEWLKRMDKNITSIRPIFEKTYGK
ESATKWIAYWRTFFISVAELFGYNNGDEWMVAHFLFRKK
>PpelNMT_KJ786957.1_-_ORF_1_(frame_2)
MARSLISLPYDLAIKAGLQMLEANLLPDFLLRRAARMLLSMRLRVSYKPTAAEQLLDLMEFAHSLKEFPISIHQDEANSQ
HYEVPAEFYKLVLGKHLKYSSSYFSKPTSRLDESEQAMLELYCERAQLVDGQKVLDLGCGWGSFSLYAAKKFPQSRITGL
SNSNTQKDFIEEECRKQGITNVNIITSDINVFEAGTTYDRIVSIEMFEHMKNYKELLKKISSWMKPDALLFVHIFVHKVF
AYHFQDSGDDDWMARSFFTGGTMPADGLLLYFQDDLSILNHWRVSGNHYSRTSEEWLKNMDRNLEPIKIIFAKTYGENEI
RKWIAYWRTFFLAVSELFGYNNGEEWMVSHYLFKRK
>NnucNMT_XM_010263690.2_-_ORF_1_(frame_2)
MDALIQVPYDATIRLMLSSLERNLLPDVVIRRLTRLLLASRLRWGYKPSSQLQLSDLLQFVHSLKEMPIAIKTDLPKSQH
YELPTSFFKLVLGKNLKYSCCYFLDKSSTLEDAEKAMLELYCERAQIKDGQSVLDVGCGWGSLSLYIAQKFSSCRITGIC
NSKTQKAYIEEQCRELKLQNVEIIVADISTFEMEASFDRILSIEMFEHMKNYKALLNKISKWMKEDSLLFVNYFCHKAFA
YHFEDKNEDDWITRYFFTGGTMPAANLLLYFQDDVSVVNHWLVNGNHYARTSEEWLKRMDQNMASIKPIMESTYGKDSAV
KWTAYWRTFFISVAELFGYNNGEEWMVALFLFKKKIN
>BsemNMT_onekp_IWMW_scaffold_2004728
MDAEQRKTVTDLMQKGLVPDDELRQICRNQCAKRLRSGYKPTHEQQQSELLKLVKSLRQMNVACDVDSLNEFTYDMPMSL
MQSVFGSTLKDSCCYFKDESMTLDEAEIAMMELYCERAQLKDGQRILDLGCGWGAVTIYIAQKYKNCHVTAITNSITQKE
FVEQRSKLLNLSNVKAILADISTHEMDDTFDRVFIVEVLEHMKNYELLLRRISKWMTNDGLLFVQHICHKTFANNGEIHG
GDWHLECMFPPGTLLVPSANLLLYFQDDLSVVNHWTLSGKNYQRTSIEWLKRLXXXALETFSGKKEDARKMTKYWRFFSI
MTAETFGYNNGEEWMSSHVLMKKK
>BvulNMT_XM_010671056.2_-_ORF_1_(frame_3)
MEKMKEMAYTATTKLMLGALERNLLPDAIIRKLTRLLLASRLRSSYKSSSQLQLSHLIHFVHSLKEMPIAIQTDTAKSQH
YELPTSFFKLVLGKHLKYSCCYFSDKQTNLDDAEKVMLDLYCERSGIEDGHTVLDVGCGWGSLSLYIAQKYSNCKVTGIC
NSTTQKAHIEDRCRELQLKNVEIIVADISTFEMEGSYDRILSIEMFEHMKNYRDLLKKISNWMKPDGLLFVHYFCHKTFA
YHFEDVNDDDWITRYFFTGGTMPAANLLLYFQDDVSIVNHWLVNGKHYAQTSEEWLKRMDRNIAYIRPIMEMTYGKDSAV
KWTVYWRTFFIAVAELFGYNNGEEWMVAHFLFKKK
>SoleNMT_XM_022000100.1_-_ORF_1_(frame_1)
MEEMKQMAYKATAKLMLGTHERNLLPDAVIRRLTRLLLAGRLRSSYKPSSQLQLSDLIQFVHSLTEMPIAIQTDTAKSQH
YELPTSFFKLVLGKHLKYSCCYFSDKQTNLDDAEKAMLDLYCERSGIQDGHTVLDVGCGWGSLSLYIAQKYSNCRVTGIC
NSVTQKAHIEDKCRDLQLKNIEIIVADISTFEMEGSYDRILSIEMFEHMKNYRDLLKKISNWMKPDGLLFVHYFCHKAFA
YHFEDVNEDDWITRYFFTGGTMPAANLLLYFQDDVSIVNHWLVNGKHYAQTSEEWLIRMDRNLAYIRPIMESTYGKDSAL
KWIVYWRTFFIAVAELFGYNNGEEWMVAHFLFKKK
>CquiNMT_XM_021888433.1_-_ORF_1_(frame_1)
MPITWPGASPHLSIQTPQFYIPIRQQKIRDTEKQSSENTGEKMEEMKQMAYKATTKLMLGAHERNLLPDALIRRLTRLLL
ASRIRSSYKSSSQLQLSDLLHFVHSLREMPIAIQTDKAKSQHYELPTSFFKLVLGKHLKYSCCYFSDKQTNLDDAEKAML
ELYCERSGIQDGHTVLDVGCGWGSLALYIAQKYSNCRVTGICNSVTQKAHIEDRCRDLQMKNVEIIVADISTFDMEGSYD
RILSIEMFEHMKNYRDLLKKISNWMKPDGLLFVHYFCHKAFAYHFEDINDDDWITRYFFTGGTMPAANLLLYFQDDVSIV
NHWLENGKHYAQTSEEWLKRMDRNLAYIRPIMESTYGKDSAVKWIVYWRTFFIAVAELFGYNNGEEWMVAHFLFKKK
>LwilNMT_UN02232_Lopophora_williamsi_CNMTlike
MPYEMSVKIMVGSLEKNLLPDAXIRRLTRLLLASRLRSGYKPTSQLQLSDLLHFVHSLREMPIAIQTDKPKSQHYELPTS
FFKLVLGKHMKYSCCYFSDEVTNLDAAEKAMLNLYCERAQLKDGHAVLDVGCGWGSLVLYVAQNYSNCRVTGICNSVTQK
AFIEERCRDLQLQNVEIKLADISTFEMEGSYDRVFSIEMFEHMKNYQDLLKKISNWMKPDGLLFIHYFCHKAFAYHFEDV
NDDDWITRYFFTGGTMPSANLLLYFQDDVSVVNHWLVDGKHYAQTSEEWLKRMDSNLGSI
RPIMESTYGKDCATKWTVYWRTFFIAVAELFGYNNGDEWMVAHFLFKKK
>HqueNMT_onekp_ZETY_scaffold_2014842
MDALVSVPYDATVRLMLASLERNILPDAVIRRLTRLLLASRLRSGYKPSSQLQLSDLLHFAHSLREMPIAIKTDDPKSQH
YELPTSFFKLVLGKHLKYSCCYFTDESNTLEDAEKAMLELYCERSQIKDGHTVLDIGCGWGSLSLYIAQKYSNCKVTGIC
NSATQKAYIEERAEDLHLQNVQIILADISTFEMEASYDRIFSIEMFEHMKNYKDLLEKISKWMKSDSLLFVHHFCHKAFA
YHYEDVNEDDWITRYFFSGGTMPSANLLLYFQDDVSVVDHWLVNGKHYAQTSEEWLKRMDKNSSTIKPIMESTYGKESAV
KWTVYWRTFFIAVAELFGYNNGEEWMVALFLFKKK
>DscaN      MT_onekp_OTAN_scaffold_2099017
MEALVQVPYEATVRVMMASLERNLLPDTVIRRLTRLLLAYRLRSCYRSSSEIQLADLLHFAISLREMPIAIKTDKPKAQH
YEVPTSFFKLVLGKNLKYSCCYFTDKSCTLEDAEKAMLEMYCERSQLKDGHTVLDVGCGWGSLSLYIAQKYSNCRITGIC
NSTTQKAFIEEQCRERQLHNVEIIVADIGTFEMDASYDRVFSIEMFEHMKNYKDLLKKISKWMKEDSLLFVHYFCHKAFA

YHFEDVNDDDWITRYFFEGGTMPSANLLLYFQDDVSIVNHWLVNGKHYAQTSEEWLKRMDKNLANVKPIMESTYGKESAV
KWTVYWRTFFIAVAELFGYNNGEEWMVAHFLFKNK
>CfloNMT_onekp_BFJL_scaffold_2193131
MDTLVQVPYDATVRLMLASLERNLLPDAVIRRLTRLLLASRLRSTYRPSSHLQLFDLLQFAHSLREMPIAIKTDEPKSQH
YELPTSFFKLVLGKNLKYSCCYFLDKSNTLEDAEKAMLELYCERSQLKDGHTVLDVGCGWGSLALYIAQKYSNCKVTGIC
NSTTQKAYIEEQARELQLQNVEIIVADISTFEMEASYDRIYSIEMFEHMKNYRDLLEKISKWMKPDGFLFVHHFCHKAFA
YHFEDVNEDDWITRYFFTGGTMPSANLLLYFQDDVSVANHWLVDGKHYAQTSEEWLKRMDKNMDSIKPIMESTYGKDLAV
KWAVYWRTFFIAVAELFGYNNGEEWMVALYLFKKK
>VodoNMT_onekp_HLJG_scaffold_2031058
MDAIVQVPYDATVRLMLASLERNLLPDSVIRRLTRLLLASRLRSGYKPSSELQLSDLLQFVHSLREMSIAIKTDEPKAQH
YELPTSFFKLVLGKNLKYSCCYFLDNLSTLEEAEKAMLESYCEKSQIKDGQTVLDVGCGWGSLSLYIAQKYSNCKITGIC
NSMTQKAYIEEQCRDLQLQNVEVIVADISTFNMEASFDRIYSIEMFEHMKNYKELLKKISNWMKPDSLLFVHHFCHKAFA
YHFEDVSEDDWITRYFFTGGTMPSSNLLLYFQDDVSVVNHWLVNGKHYAQTSEEWLKRMDKNASSIKPIMELTYGKDSAV
KWTVYWRTYFIAVAELFGYNNGDEWMVAFFLFKKK
>ScanNMT_onekp_QRNU_scaffold_2171003
MLTSLERNILPDAVIRRLTRVLLGSRLRSGYKSSSQLQLSELIHFIHALKEMSIAIKTDEPKVQHYELPTSFFKLVLGKN
LKYSCCYFPETSSTLEEAEKAMLESYCEKSQIKDGQTVLDVGCGWGSLSLYIAQKYSNCKITGICNSNTQKAFIEEQCRD
LQLQNVDIIVADISTFDMEALFDRVFSIEMFEHMKNYKELLKKISRWMKPDSLLFVHHFCHKAFAYHFEDVNEDDWITRY
FFTGGTMPSANLLLYFQDDVSVVDHWLVNGKHYAQTSEEWLKRMDKNMSSIKPIMESTYG
KDSAVKWTVYWRTFFIAVAELFGYNNGEEWMVAHFLFKKK
>VoffNMT_onekp_FFFY_scaffold_2013319
MQIPYDATVRLMLTSLERNFLPDAVIRRLTRLLLSGRLRSGYKSSADLQLSDLLQFVHSLREMPIAIKTDEPKVQHYELP
TSFFKFVLGKNLKYSCCYFRDESSTLEDAEKAMFESYCEKSQLKDGQTVLDVGCGWGSLSLYIAQNYSNCQITGICNSRT
QKAFIEDQCRDLKLENVEIIVADISTFEMEASYDRIFSIEMFEHMKNYKELLKKISGWMKSDGFLFVHHFCHKSFAYHFE
DLNDDDWITRYFFSGGTMPSANLLLYFQDDVSVVDHWLLNGKHYAKTSEEWLKRMDKHSSAIKPIMESTYGKDSAVKWTV
YWRTFFISVAELFAYNNGDEWMVASFLFKKK
>LsatNMT_XM_023880049.1_-_ORF_1_(frame_2)
MSNLLQVPYNATVKLMLTSLERNLLPDAVIRRLTRLLVAGRLRNCFKPTSQEQLQDLMAFVHSLEEMPIAVKTEDAKTQH
YELPTSFFKLVLGKNFKYSCCYFRDKLSTLEDAEVAMLELYCEKAQLKDGHTVLDVGCGWGSLIIYIAKKFSNCKVTGIC
NSVTQKAHIEDQCGKLSLQNVEIIVADISTFEMEGSYDRIFSIEMFEHMKNYKDLLKKISNWMKEDAFLFVHFFCHKTYA
YHFEDVSEDDWITRYFFSGGTMPSANLLLYFQDDVSVVDHWLVNGKHYAQTSEEWLKRMDKNMASIKPIMESTYGKDSAV
KWTVYWRTFFISVAELFGYNNGEDWMVTHYLFKKK
>HannNMT_XM_022172464.1_-_ORF_1_(frame_3)
MSSLLQAPYDATVKLMSASLERNLLPDAVMRRLTRLLLAGRLRSCFKPTSQQQLQQLLEFVHSLKEMPIAVKTEDAKSQH
YELPTSFFNLVLGKNFKYSCCYFRDKSSTLEDAEEAMLQIYCEKSELQDGHTVLDVGCGWGSLSLYIAQKYSNCKVTGIC
NSVTQKAHIEEQCEKLGLENVEIIVADISTYEMDGSYDRIFSIEMFEHMKNYKDLLKKISKWMKSDGFLFVHHFCHKTYA
YHFEDVNEDDWITRYFFSGGTMPSSNLLLYFQDDVSVVNHWLVNGKHYAQTSEEWLKRMDKNIKAIQPIMESTYGKDSAV
KWTVYWRTFFLSVAELFGYNDGEEWMVTHYLFKKK
>PresNMT_onekp_SALZ_scaffold_2069385
MNSIVQLPYDAIMRMMLGSLDRNLLPDAVIRRLTRLLLASRLRSGYKPSAELQLSELLQFAHSLREMSIAIKTEEPKYQH
YEVPTSFFKLVLGKNLKYSCCYFLDKSSTLEDAEKAMLELYCERSKIEDGHTVLDVGCGWGSLSIYIAQKYKKCKVTGIC
NSTTQKVFIEEHCRNLQLENVEVIVADISMFDMEASYDRIFSIEMFEHMKNYKELLKKISRWMNPDSLLFVHHFCHKAFA
YHFEDRSEDDWITRYFFTGGTMPSANLLLYFQDDVSVVNHWLVNGKHYAQTSEEWLKRMDKNSSSVRPIMESTYGEDSAV
RWTVYWRTFFIAVAELFAYNNGEEWMVTHFLFKKK
>DcarNMT_XM_017369326.1_-_ORF_1_(frame_2)
MTLKKMMEVPYAATVRVMLASLERNLLPDAVIRQLTRLLLSSRLRLGYAPSAHLQLAQLLHFAHSLRDLPIAIKTEDPKV
QHYELPTSFFKLVLGENLKYSCCYFNDTSSTLDDAEKAMLETYCERSGLEDGHTVLDVGCGWGSLSIFIAKKYSNCKITG
ICNSNTQKAFIEERCRDLDLHNVEIIVADISTYDMEASYDRIFSIEMFEHMKNYKELLKKISRWMKPDSLLFVHHFCHKT
FAYHFEDIDEDDWITRYFFSGGTMPSANLLLYFQDDVSVVNHWLVNGKHYAQTSEEWLKRMDKNMGSVRPIMESTYGKDS
AVKWTVYWRTFFIAVAELFGYSNGEEWMVAHFLFKRK
>CasiNMT_onekp_WEQK_scaffold_2009947
MGFEMNTLVQLPYEATVKMMLSSLERNLLPDAVTRRLTRLLLAGRLRSGYKPSAEXLLQFAHSLREMSIAIKTEEPKSQH
YELPTAFFKLVLGNNLKYSCCYFHDTLSTLDDAEKAMLELYCERSQIADGHTVLDVGCGWGSLSLYIAKKYSRCKITGIC
NSTTQKAFIDEQCRDLQLQNVEVIVADISTFDMEASYDRIFSIEMFEHMKNYKDLLKKISQWMKPDSLLFVHHFCHKTFA

YHFEDLNQDDWITRYFFTGGTMPSANLLLYFQDDVSVVNHWLVNGKHYAQTSEEWLKRMDSNLSSVRPIMESTYGKDSAV
KWTVYWRTFFIAVAELFGYNNGEEWMVTHFLFKKK
>HumbNMT_onekp_OINM_scaffold_2000376
MNTIAKLPYDATVRMMLASLERNLLPDALIRRFTRLLLASRLRSGYKSSSQLQLSDLLHFAHSLKEMSIAIKTEEPKYQH
YELPTSFFKLVLGDNLKYSCCYFSDKSSTLEDAEKAMLEMYCERSKLEDGHTVLDVGCGWGSLSLYIAKKYSKCKVTGIC
NSTTQKAFIEEQCRDLQLQNVEIIVADISTFDMEASYDRVFSIEMFEHMKNYKELLKKISRWMHPDSLLFVHHFCHKAFA
YHFEDISDDDWITRYFFTGGTMPSANLLLYFQDDVSVVNHWLVNGKHYAQTSEEWLKRMDKNLSSVRPIMESTYGKDSAV
KWTVYWRTFFIAVAELFGYNNGEEWMVAHFLFKKK
>AarcNMT_onekp_TQKZ_scaffold_2060644
MALKTMMEVPYAATVRMMLASLERNLLPDAVIRRLTRLLLSTRLRLGYAPSAHLQLAQLLHFAHSLRDMPIAIKTEDAKV
QHYELPTSFFKLVLGENFKYSCCYFNDTSSTLDDAEKAMLETYCERSGLEDGHSVLDVGCGWGSLSIYIAKKYSKCKITG
ICNSTTQKASIEERCRDLNLHNVEIIVADISTFDMEASYDRIFSIEMFEHMKNYKELLKKISQWMKPDSLLFVHHFCHKT
FAYHFEDINEDDWITRYFFSGGTMPSANLLLYFQDDISVVDHWLVNGKHYAQTSEEWLKRMDKNMSSVRPIMESTYGKDS
AVKWTVYWRTFFISVAELFGYNNGEEWMVAHFLFKRK
>CrosNMT_onekp_UOYN_scaffold_2041458
MPIAIKTEDAKSQHYELPTSFFKLVLGKHLKYSCCLFSDNSNNLEDAEKAMLELYCERAKLKDGHAVLDVGCGWGSLSLY
IAKKYSNCKVTGICNSATQKAHIEEQCGDHQLQNLEIIVADISTFEMEASYDRIFSIEMFEHMKNYRDLLKKISMWMKPD
SLLFVHHFCHKTFAYHYEDVNEDDWITRYFFSGGTMPSANLLLYFQDDVSVVDHWLVNGNHYAQTSEEWLKRMDQNMSSV
KPIMESTYGKDSAVKWIVYWRTFFIAVAELFGYNNGEEWMVVHFLFKKK
>SindNMT_XM_011083340.2_-_ORF_1_(frame_2)
TEKPKSQHYEVPTSYFKLVLGKHLKYSCCFFPNKSSTLEDAEKAMLELYCERSQIKDGQSVLDVGCGWGSLSIYIAQKYP
NCQVTGICNSITQKAHIEEQCRELQLQNVEIIVADISTFDMEASYDRIFSIEMFEHMKNYQDLLKKISKWMKPDSFLFVH
YFCHKTFAYHFEDVNEDDWITRYFFTGGTMPSANLLLYFQDDVSIVNHWLVNGKHYAQTSEEWLKRMDQNLNSIKPIMEC
TYGKDSAVKWTVYWRTFFIAVAELFGYNNGEEWMVAHFLFKKK
>OeurNMT_XM_023036948.1_-_ORF_1_(frame_2)
YEVPTSFFKLVLGKHLKYSCCFFSDKSSTLEDAEKAMLELYCERSQIKDGHAVLDVGCGWGSLALYIAKKYPNCQITGIC
NSVTQKAYIEEQCRDLQVQNIDIVVADISTFDMEASYDRVFSIEMFEHMKNYRDLLKKISRWMKPDGLLFVHYFCHKTFA
YHFEDVNEDDWITRYFFDGGTMPSANLLLYFQDDVSIADHWLINGKHYAQTSEEWLKKMDKNLNSVKPIMESTYGKDSAV
KWTVYWRTFFIAVAELFGYNDGEEWMVSHFLFRKK
>DpurNMT_onekp_GNRI_scaffold_2132022
MASANGIVDSIVQMPYDATVRLMLSSLERNLLPDAVVRRLTRLLLASRLRSGYRSSAEIQLSDLLQFVHSLKEMPIAIKT
ETPKSQHYEVPTSFFNLVLGKHLKYSCCYFPDKSSTLEDAEKAMLELYCERSQIEDGHSVLDVGCGWGSLSLYIAQKYPN
CKITGICNSATQKAFIEEQSRGLQLQNVEIVVADISTFEMEASYDRIFSIEMFEHMKNYKDLLKKISGWMKPDAHLFIHY
FCHKTFAYHFEDVNEDDWITRYFFTGGTMPSANLLLYFQDDVSIVNHWLVNGKHYAQTSEEWLKRMDQNLSSIKPIMEST
YGKDNAVKWTVYWRTFFISVAELFGYNNGEEWMVAHFLFKKK
>PdulNMT_onekp_MQIV_scaffold_2003605
MAAVKGGVMDSVVQVPYGATVRLMLASLERNLLPDAVVRRLTRLLLAGRLRSGYRSSADIQLSELLQFAQSLREMPIAIK
TEKAKSQHYELPTPFFKLVLGKHLKYSSCYFPDKSSSLEDAEKAMLELYCERSQIKDGHSVLDVGCGWGSLVLYTAQKYP
NCQITGICNSTTQKAHIEEQCRDLQLQNVEIIVADISTFDMEASYDRIFSIEMFEHMKNYHDLLKKISKWMKPDGLLFVH
HFCHKTFAYHFEDLNDDDWITRYFFEGGSMPSANLLLYFQDDVSIVNHWLVNGKHYAQTSEEWLKRMDQNVSSIKPIMES
TYGKDSAVKWTVYWRTFFITVAELFGYNNGDEWMVAHFLFRKK
>SdivNMT_onekp_EQDA_scaffold_2056100
MLASLERNLLPDAVVRRLTRLLLASRLRSGYRPSAEIQLSDLIHFAHSLREMPIAIKTEEPKYQHYEVPTSFFKLVLGKH
LKYSCCFFPDKSSTLEDAEKAMLELYCERSQMKDGHSVLDVGCGWGSLSLFLAQKYPNSQITGICNSTTQKSHIEEQCRD
LDLQNVVIIVADISSFEMEASYDRIFSIEMFEHMKNYHDLLKKISRWMKPDSLLFVHYFCHKAFAYHFEDVNDDDWITRY
FFTGGTMPSANLLLYFQDDVSIVNHWLVNGKHYAQTSEEWLTRMDRNSSLIKPIMEATYGKDSAVKWMVYWRTFFIAVAE
LFGYDNGEEWMVAHFLFKKK
>SlycNMT_AK321345.1
MDAIVQLPYDATVRLMLRSLERNLLPDTVIRRLTRLLLAARLRSGYKPSSELQLSDLLQFVQSLKEMPIAVMTEKPKSQH
YELSTSFFKIVLGKHLKYSCCYFKDKLSTLEDAEKAMMELYCERSQLKDGHTVLDVGCGWGSLSLYIAQKYSACKVTGIC
NSVTQKAHIEEQCRELQLQNLEIIVADISTFEMEGSYDRILSIEMFEHMKNYGDLLKKISRWMKADSLLFVHYFCHKAFA
YHFEDVNDDDWITRYFFSGGTMPSANLLLYFQDDVSVVDHWLVNGKHYAQTSEEWLKRMDENKSSIKPIMESTYGKDSAV
KWTVYWRTFFLSVAELFGYNNGEEWMVAHFLFKKN
>Ipur_onekp_VXKB_scaffold_2003363

```
MKSIVQVPYEATVRVMLATLERNLLPDAVIRRLTRLLLAARLRSAYKPSAELQLSDLLHFVHCLKEMPIAIMTEKPKSQH
YELPTSFFKLVLGKQFKYSCCFFPEQLSSLEDAEKAMLELYCERSQIKDGHTVLDIGCGWGSLSLYIANKYSNCKVTGIC
NSLTQKEHIQQQCRELELENVDIIVADISTFDMEASYDRIFSIEMFEHMKNYGQLLKKISKWMKPDSFLFVHYFCHKTFA
YHFEDMNDDDWITRYFFSGGTMPSANLLLYFQDDVCVIDHWLVNGKHYARTSEEWLKRMDENIKSVKPIMESTYGKDSAL
KWTVYWRTFFIAVAELFGYNNGEEWMVAHFLFKKK
>InilNMT_XM_019306366.1
MKSIVQVPYEATVRVMLATLERNLLPDAVIRRLTRLLLAARLRSAYKPSAELQLCDLLHFVHSLKEMPIAIMTEKPKSQH
YELPTSFFKLVLGKQFKYSCCFFPDQYSTLEDAEKAMLELYCERSQIKDGDTVLDVGCGWGSLSLYIANKYSNCKVTGIC
NSVTQKEHIEQQCRDLQLENVDIIVADISTFDMEASYDRIFSIEMFEHMKNYGQLLKKISKWMKPDSFLFVHYFCHKTFA
YHFEDINDDDWITRYFFSGGTMPSANLLLYFQDDVSVIDHWLVNGKHYARTSEEWLKRMD
>NtabNMT_XM_016588651.1
MDAIVQVPYNATVRLMLSSLERNLLPDAVIRRLTRLLLAARLRSSYKPSAELQLSDLLHFVHSLKEMPIAVMTEKAKSQH
YELPTSFFKFVLGKHFKYSCCYFRDKSSTLEDAEKAMMELYCERSQLKDGHTVLDVGCGWGSLSLYIAQKYCSCKVTGIC
NSVTQKAHVEEQCREFQLQNVEIIVADISTFEMEGSYDRILSIEMFEHMKNYGYLLKKISRWMKPDSLLFVHHFCHKAFA
YHFEDVNDDDWITRYFFSGGTMPSANLLLYFQDDVSVVNHWLVNGKHYAQTSEEWLKRMDQNKTSIKPIMESTYGKDSAV
KWTVYWRTFFISVAELFDYNNGEEWMVAHFLFKKK
>MpenNMT_onekp_DAYQ_scaffold_2055658
MDALVQVPYDATVRVMLASLERNMLPDALIRRLTRALLSNRLRTGYKPSSELQLSDLLHFAHSLREMPIAVKTDEPKAQH
YELPTSFFKLVLGKHFKYSCCYFNDKSTTLEDAEKAMLELYCEKSQIQDGHTVLDVGCGWGSLSLYIAQKYSNCKITGIC
NSTTQKAFIEEQCRDLQLQNVEIIVADISTFDMEASYDRIFSIEMFEHMKNYEDLLKKISKWMKQDSLLFVHHFCHKAFA
YHFEDLSDDDWITRYFFTGGTMPSANLLLYFQDDVSVVNHWLVNGKHYAQTSEEWLKRMDKNMASIKPIMETTYGKDSAV
KWTVYWRTFFIAVAELFGYNNGEEWMVTLFLFKKK
>PlacNMT_onekp_HTIP_scaffold_2010395
MAALVQAPYDATVRLMLASLERNLLPDVVIRRLTRLLLANRLRSGYKSSSELQLSDLLQFVHSLREMPIAITTDVPKSQH
YELPTSFFKLVLGKNLKYSCCYFHDKSKTLEDAEKAMLEIYCERSQIKDGQSVLDVGCGWGSLSLYIAQKYINCKITGIC
NSTTQKEYIMEQCRDLQLKNVEIIVADISTFDMEATYDRIISIEMFEHMKNYKDLLKKLSKWMKQDSLLFVHHFCHKTFA
YHFEDLNDDDWITRYFFTGGTMPSANLLLYFQDDVSVVNHWLVNGKHYAQTSEEWLKRMDQNVDSIKAIMESTYGKDSAV
KWTVYWRTFFIAVAELFAYNNGEEWMVANFLFKKK
>HvirNMT_onekp_YHXT_scaffold_2057326
MDALAQVPYDATVRLMMASLERNLLPDAVIRRLTRLLLASRLRSGYKSSSEPQLSDLLHFAHSLKEMPIAIKTDKPKSQH
YELPTSFFKLVLGKNLKYSCCYFHDKSSTLEDAEKAMLELYCERSQIKDGHTVLDVGCGWGSLSIYLAQKYSNCRITGIC
NSATQKAFIEEQCRDRQLQNVEIIVADISTFDMKGSYDRIFSIEMFEHMKNYKDLLKKISRWMKQDSLLFVHHFCHKAFA
YHFEDVNEDDWITRYFFTGGTMPSANLLLYFQDDVSVVNHWLVNGKHYAQTSEEWLKRMDQNLASIKPIMESTYGKELAV
KWTVYWRTFFIAVAELFGYNDGEEWMVALFLFKKK
>PeupNMT_XR_841009.1_-_ORF_1_(frame_1)
MERLMQVPYEATVKVMLTSLERNLLPDAVIRRLTRMLVAYRLRSCYRTSSELQLADLLQFVHSLKEMRIAIETDKPKTQH
YELPTSFFKLVLGKNLKYSCCYFSDKSNTLEDAEKAMLELYCERSQLKDGHTVLDVGCGWGSLSLYIAQKYSNCKITGIC
NSTTQKVHIDEQCRDLQLQNVEIIVADISTFEMQASYDRIYSIEMFEHMKNYGDLLNKISKWMKQDGLLFVHYFCHKAFA
YHFEDVNEDDWITRYFFTGGTMPSANLLLYFQDDVSIVDHWLVNGKHYSQTSEEWLKRMDRNLAAIKTIMESTYGKDQAV
KWTVYWRTFFIAVAELFGYNNGEEWMVAHFLFNKK
>PtriNMT_XM_002324706.3_-_ORF_1_(frame_2)
MERLMQVPYEATVKVMLASLERNLLPDAVIRRLTRMLLADRLRSCYKTSSELQLADLLQFVHSLKEMPIAIKTDKPKTQH
YELPTSFFKLVLGKNLKYSCCYFSDKSNTLEDAEKAMLELYCERSQLKDGHTVLDVGCGWGSLSLYIAQKYSNCKITGIC
NSTTQKVHIDEQCRDLQLQNVEIIVANISTFEMQASYDRIYSIEMFEHMKNYGDLLNKISKWMKQDGLHFVHYFCHKQFA
YHFEDVNEDDWITRYFFTGGTMPSANLLLYFQDDVSIVDHWLVNGKHYSQTSEEWLKRMDRNLAAIKPIMESTYGKDQAV
KWTVYWRTFFIAVAELFGYNNGEEWMVAHFLFNKK
>PcaeNMT_onekp_SIZE_scaffold_2058361
MEGLMKVPYDATVRVMLGSLERNLLPDAVVRRLTRLLLASRLRSGYKPSSELQLSDLLQFVLSLKEMPIAIQTDKPKTQH
YELPTSFFKLVLGENMKYSCCYFPKESSSLEDAEKAMLELYCERAQLKDGHTVLDVGCGWGSLSLFIAKKYANCRVTGIC
NSTTQKEYIEERCRDLKLQNLEIIVADISTFEMEASYDRVFSIEMFEHMKNYEQLLKKISKWMKQDSLLFVHYFCHKSFA
YHFEDINEDDWITRYFFTGGTMPSANLLLYFQDDVTIVNHWLVNGKHYSKTSEEWLKRMDMNLATIKPIMESTYGKDQSL
KWTVYWRTFFISVAELFGYNNGEEWMVANFLFRKK
>VcanNMT_onekp_NJLF_scaffold_2004583
MPIAVQTDKPKTQHYELPTSFFKLVLGRNMKYSCCYFSDNSSTLEDAEKAMLELYCERSDLKDGHTVLDVGCGWGSLTLY
IAQKYSNCKITGICNSNTQKEFIEEQCRDLHLQNLEIIVADISTFEMEASYDRIFSIEMFEHMKNYGKLLKKISKWMKED
```

GLLFVHYFCHKAFAYHFEDTNEDDWITRYFFTGGTMPSANLLLYFQDDVSIVNHWLVNGKHYAQTSEEWLKRMDQKLASI
KPIMESTYGKDQAVKWTVYWRTFFISVAELFGYNNGEEWMVAHFLFKKKCLFT
>SfarNMT_onekp_RZTJ_scaffold_2015852
MEGLMQVPYEATVKVMLASLEKNLLPDAVIRRLTRLLLAHRLRSCYKTSSELQLTDLLQFVHSLKEMPIAIATDKPKTQH
YELPTSFFKLVLGKNLKYSCCYFSDKSNTLEDAEKAMLELYCERSQLKDGHTVLDVGCGWGSLSLYIAQKYSNCKITGIC
NSTTQKVHIDEQCRDLQLQNVEIIVADISTFEMQSSYDRIYSIEMFEHMKNYGALLNKISKWMKQDGLLFVHYFCHKAFA
YHFEDVNEDDWISRYFFTGGTMPSANLLLYFQDDVSIVDHWLVNGKHYSQTSEEWLKRMDRNFASIKPIMESTYGKDQTI
KWTVYWRTFFIAVAELFGYNNGEEWMVAHFLFNKK
>SpurNMT_onekp_LFOG_scaffold_2018555
MEGLMQVPYEATVKVMLASLEKNLLPDAVIRRLTRLLLAHRLRSCYKTSSEXXDLLQFVHSLKEMPIAIVTDKPKTQHYE
LPTSFFKLVLGKNLKYSCCYFSDKSNTLEDAEKAMLELYCERSQLKDGHTVLDVGCGWGSLSLYIAQKYSNCKITGICNS
TTQKVHIDEQCRDLQLQNVEIIVADISTFEMQASYDRIYSIEMFEHMKNYGALLNKISKWMKQDGLLFVHYFCHKAFAYH
FEDVNEDDWISRYFFTGGTMPSANLLLYFQDDVSIVDHWLVNGKHYSQTSEEWLKRMDRNFASIKPIMESTYGKDQTIKW
TVYWRTFFIAVAELFGYNNGEEWMVAHFLFNKK
>LperNMT_onekp_LFII_scaffold_2015141
MLGSMERNILPDIVVRSLTRLLLGVRLRWGYKSCAELQLSQLLNFVHSLKDMPIAIQTEKPKEQHYEVPTSFFKLVLGKN
LKYSCCYFSDKTSSLENAEDAMLELYCERSELKDGQSVLDVGCGWGSLALYIAQKYSNCKVTGICNSTTQKAFIEDQCRD
RQLQNVEIIVADISTFEMDASFDRIYSIEMFEHMKNYRDLLNKISRWMNQDGRLFVHHFCHRVFPYHFEDKYEEDWLARY
FFSGGTMPSANILLYFQDDVSVVNQWHVNGNHYAQTSEEWLKRMDKNMGIIKPIMASTYGKDEALKWTVYWRTFFMAVAE
LFRYNNGEEWLVTHYLFKKK
>JregNMT_XM_018981024.1_-_ORF_1_(frame_1)
MEGGLLQGPYDATVQLMLASLERNLLPDAVIRRLTRLLLAGRLRSEYKPSSELQLSHLLQFVHSLREMPIAIKTDEPKAQ
HYELPTSFFELVLGKNLKYSCCYFSDKSKTLEDAEEAMLELYCERSQLKDGHAVLDVGCGWGSLSLYIANKYRNCKVTGI
CNSTTQKAFIEKQCGDLQLQNLEIIVADISTFEMETSYDRIFSIEMFEHMKNYKDLLKKISKWMKQDSLLFVHHFCHKAF
AYHFEDINEDDWITRYFFTGGTMPSANLLLYFQDDVSVVDHWLVNGNHYAQTSEEWLKRMDKNSTSIKPIMESTYGKDSA
VKWTVYWRTFFIAVAELFGYNNGEEWMVALYLFKKK
>QsubNMT_XM_024023446.1_-_ORF_1_(frame_2)
MDKIMQVPYEATVRLMLGSLERNLLPDAVIRKLTRLLLATRLRSGYKPSAQLQLSHLLQFVLSLREMPIAIKTGDPKTQH
YELPTSFFKLVLGNNLKYSCCYFSDNSRTLENAEETMLELYCERSQLKDGHTVLDVGCGWGSLSLYIARKYSNCRVTGIC
NSTTQKAHIEEQCRDLQLQNVEIIVADISIFEMEASYDRIFSIEMFEHMKNYEDLLKKISKWLKEDSLLFIHHFCHKAFA
YHFEDINEDDWITRYFFSGGTMPSANLLLYFQDDVSVVNHWLVNGKHYAQSSEEWLKRMDKNLSSIKPIMESTYGKDSAV
KWTVYWRTFFIAVAELFGYNDGEEWMVVHFLFKKK
>AserNMT_onekp_LWDA_scaffold_2084080
MVGLMQVPYDATVRLMLSTLERNLLPDAVIRRLTRLLLAGRLRSGYKPSSELQLSDLLEFVLSLREMPIAIKTNEPKAQH
YELPTSFFKLVLGKNLKYSCCYFSDKSKTLEDAEVAMLELYCERSQLKDGHAVLDVGCGWGSLSLYIAQKYSNCMVTGIC
NSTTQKAYIDQQCRDLQLQNVEIIVADISTFEMEASYDRIYSIEMFEHMKNYKDLLKKISNWMKQDGLLFVHHFCHKAFA
YHFEDINDDDWITRFFFTGGTMPSANLLLYFQDDVSVVNHWLVNGKHYAQTSEEWLKRMDKKSASIKPIMESTYGKDSAV
KWTVYWRTFFIAVAELFNYNDGEEWMVALFLFKKK
>CpumNMT_onekp_UZWG_scaffold_2002461
MQVPYETTVRLMLGSLERNLLPDAVIRKLTRLLLATRLRSGYKPSAQLQLSHLLQFVLSLREMPIAIKTDDPKTQHYELP
TSFFKLVLGNNLKYSCCYFSDNSKTLENAEETMLELYCERSQLKDGHTVLDVGCGWGSLSLYIARKYSNCRVTGICNSTT
QKAYIEEQCRDLRLQNVEIIVADISVFEMEASYDRIFSIEMFEHMKNYEDLLKKISKWLKEDSLLFIHHFCHKAFAYHFE
DINEDDWITRYFFSGGTMPSANLLLYFQDDVSVVNHWLVNGKHYAQTSEEWLKRMDKNLSSIKPIMESTYGKDSAVKWTV
YWRTFFIAVAELFGYNDGEEWMVVHFLFKKK
>PmumNMT_XM_008222301.2_-_ORF_1_(frame_1)
MNRIMQAPYDATVRFALSSLERNLLPDAVVRRLTRLLLASRLRSGYKPTSELQLSDLLQFVQSLKEMPIAINTDDPKAQH
YEVPTSFFKIVLGKNLKYSSCYFTDGSSTLEEAEKAMLELYCERSQIKDGHTVLDVGCGWGSLPLYIAQKYSNCKVTGIC
NSTTQQAFIEEQCRNLQLQNVEIIVADISTFEMEASYDRIFSIEMFEHMKNYKYLLKKISRWMKQDCLLFVHHFCHKAFA
YHFEDKSEDDWITRYFFSGGTMPSANLLLYFQDDVSIVNHWLVNGKHYAQTSEEWLKRMDQNMASIKPIMESTYGKDSAV
KWTVYWRTFFISVAELFGYNNGEEWMVAHFLFEKK
>RchiNMT_XM_024311715.1_-_ORF_1_(frame_1)
MDRVMQAPYDATVRFALASLERNLLPDAVIRRFTRLLLASRLRSGYQPSSELQLSQLLRFVKSLKEMPIAIKTDDAKAQH
YEVPTSFFKMVLGNNLKYSCCYFTDKSSSLEDAEKAMLEMYCERSQIKDGHTVLDVGCGWGSLSLYIAQKYKNCRVTGIC
NSATQKAFIEEQCRNRRLENVEIIVADISTFEMEASYDMIFSIEMFEHMKNYKDLLKKISAWMKQDSLLFVHHFCHKAFA

YHFEDKSDDDWITRYFFSGGTMPSANLLLYFQDDVSVVDHWLVNGKHYAQTSEEWLKRMDHNSASIKPIMESTYGKDSAV
KWTVYWRTFFIAVAELFGYNNGEEWMVALFLFKKK
>CsatNMT_onekp_DGNP_scaffold_2037631
MAALVQLPYDATVKLALASLERNLLPDAVIRRLTRLLLAARLRSGYKSSAHLQLSDLLRFIHSLKEMPIAIKTDEPKAQH
YELPTSFFNLVLGQNFKYSCCYFEKSSSTLEEAEKAMLEMYCEKSKLEDGQTVLDVGCGWGSLSLYIAQKYKNCKVTGIC
NSSTQKDHIDQRCRELEVENVEIIVADISTFEMERSFDRIYSIEMFEHMKNYKELLKKISGWMKEDALLFVHHFCHKTFA
YHFEDVNEDDWITRYFFTGGTMPSANLLLYFQDDVSVIDHWLVNGKHYAQTSEEWLKRMDKNVRLIKPIMESTYGKENAV
KWTVYWRTFFIAVAELFGYNNGEEWMVALFLFKKK
>CsatNMT_XM_004141284.2_-_ORF_1_(frame_2)
MAKLIQIPYDATVHLALASLERNLLPDAIIRTFTRLLLASRLRSGYKPSSQLQLSELLHFVHSLREMPIAIKTDKPKAQH
YEVPTSFFKLVLGKNLKYSCCYFNDKSSTLEDAEDAMLQMYCERSQLKDGHTVLDVGCGWGSLSLYIAQKYKNCTVTGIC
NSITQKAYIEDRCQDLQLHNVNIIVADISTYEMEAEYDRIFSIEMFEHMKNYKDLLKKISGWMKQDSLLFVHYFCHKVFA
YHFEDVNDDDWITRYFFEGGTMLSSNLLLYFQDDVSIVDHWLVNGKHYSQTSEEWLKRMDENIASIKPIMATTYGKDSAV
KWTVYWRTFFIAVAELFGYNNGEEWMVSHFLFKKK
>CmelNMT_XM_008454536.2_-_ORF_1_(frame_3)
MIYCCSCCYFNDKSSTLEDAEDAMLQMYCERSQLKDGHTVLDVGCGWGSLSLYIAQKYKNCTVTGICNSITQKAYIEDRC
QDLQLHNGNIIVADVRKYEMETEYYDRIFSIGMFEHMKNYKDLLKKISGWMKQDSLLFVHYFCHRVFAYHFEDVNEDDWI
TRYFFEGGTMPSSNLLLYFQEEVSIVDHWLVNGKHYSRTSEEWLKRMDKNIASIKPIMATTYGKDSAAKWTVYWRTFLIA
VAELFDYNNGEEWMISQFLFKKK
>BegoniaNMT_onekp_OCTM_scaffold_2026880
AHSLKEKPIAIQTDEPKVQHYELPTSFFKLVLGENLKYSCCYFDDNSKSLEEAEKAMLELYCERSQLEDGQTVLDVGCGW
GSLSLYIAQNYRNCRVTGICNSITQKAYIDGKSRDLRLDNVEIIVADISNFEMEAVFDRIFSIEMFEHMKNYKDLLKKIS
RWMKEDSLLFVHYFCHKAFAYHFEDNNDDDWITRYFFTGGTMPSANLLLYFQDDVSVVNHWLVNGKHYAQTSEEWLKRMD
KNMASIRPIMASTYGNAAAVKWTVYWRTFFIAVAELFGYNDGEEWMVALFLFKKK
>CariNMT_XM_004501390.2_-_ORF_1_(frame_1)
MEGMMQLPYKTTVKLMLGSLERNLLPDAIIRRFTRLLLATRLRSGYKSSSQLQLSHLLQFVHSLQELPIAVNTDKPKSQH
YELPTSFFRLVLGNNLKYSCCYFSSASKTLEDAEEEMLKLYCERSNLEDGHTVLDVGCGWGSLTLYIAKNYSNSRVTGIC
NSTTQKAYIEEKCLELQLQNVDIIVADISTFEMEASYDRVFSIEMFEHMKNYKDLLNKISKWMKEDSLLFVHHFCHKAFA
YHFEDNNEDDWITRYFFTGGTMPAANLLLYFQDDVSVVNHWLVNGKHYAQTSEEWLKRMDKNMSSIKPIMESTYGIDSAT
KWTVYWRTFFIAVAELFGYNNGEEWMVAHFLFKKK
>PvulNMT_XM_007136799.1_-_ORF_1_(frame_1)
MEGMMQLAYGATVKLMLSALERNLLPDAVTRRLTRVLLATRLRSSSNTSSDLQLSHLLHFAHSLQEMPIAINTEKAKSQH
YELPTAFFKLVLGSNLKYSCCYFSSASMTLEDAEEAMLKLYCERSNLKDGHTVLDVGCGWGSLALYIAKNYTNCRVTGIC
NSTTQKAYIEEKCRDLQLQNMNIIVADISTFEMEASYDRIFSIEMFEHMKNYKELLKKISKWMKEDSLLFVHYFCHKAFA
YHFEDKNEDDWITRYFFSGGTMPSANLLLYFQDDVTVINHWLVNGKHYSQTSEEWLKRMDQRMTYIKPIMQSTYGNDSAT
KWTAYWRTFFISVAELFGYNNGEEWMVAHFLFKKK
>GmaxNMT_XM_003522226.3_-_ORF_1_(frame_3)
MEGTMQLAYETVVKLMLAALERNVLPDVITRRLTRLLLATRLRSAYKPSSQLQLSDLLYFAHSLQEMPIAINTDKPKSQH
YELPTAFFKLVLGNNLKYSCCYFSSASMTLDDAEEAMLKLYCERSNLKDGHTVLDVGCGWGSLALYIAKNYTNCRVTGIC
NSTTQKAYIEEKCRDLQLQNLNIIVADISTFEMETSYDRIFSIEMFEHMKNYKDLLKKISKWMKEDSLLFVHYFCHKAFA
YHFEDKNEDDWITRYFFTGGTMPSANLLLYFQDDVTVTNHWLVNGKHYAQTSEEWLKRMDQRMTFIKPIMQSTYGKDSAT
KWTAYWRTFFIAVAELFGYNNGEEWMVAHFLFKKK
>MtruNMT_BT136714.1_-_ORF_1_(frame_2)
MEKMEEMMHLPYNTTVKLMLGSLERNLLPDTVIRRLTRLLLSTRLRSSYKPSSELQLSDLLQFAHSLQDMPIAVSTDVPK
SQHYELPTSFFKLVLGNNLKYSSCYFSSASKTLEDAEEEMLKLYCERSNLKDGHTVLDVGCGWGSLTLFIAKNYSSSRVT
GICNSTTQKAFIEEKCRELQLQNVDIIVADISTFEMEASYDRIFSIEMFEHMKNYKDLLKKISIWMKEDGLLFVHHFCHK
AFAYHFEDKNEDDWITRYFFTGGTMPAANLLLYFQDDVTVINHWLVNGKHYAQTSEEWLKRMDKNKTSIKPIMESTYGKD
SATKWTVYWRTFFIAVAELFGYNNGEEWMVAHFLFKKK
>CcanNMT_onekp_RKFX_scaffold_2004511
MEGIMQLPYGITVKLMLGSLERNLLPDAVIRRLTRLLLATRLRSGYKPSSQLQLSDLIQFVHSLKEMPIAINTDKPKSQH
YELPTSFFKLVLGNNLKYSCCYFSSASKTLEEAEEAMLELYCERSQMKDGHAVLDVGCGWGSLCLYIAKKYSSSRVTGIC
NSTTQKAHIEEKCRDLQLQNVEIIVADISAFEMETSYDRIFSIEMFEHMKNYKDLLEKISKWMKEDALLFVHHFCHKAFA
YHFEDINEDDWITRYFFSGGTMPAANLLLYFQDDVTIVNHWLVNGKHYAQTSEEWLKRMDKNLTSIKPIMETTYGKDSAV
KWTAYWRTFFIAVAELFGYNNGEEWMVAHFLFKKK
>EgraNMT_XM_010049066.2_-_ORF_1_(frame_2)

MEKMMQVPYEATVKVMMASLERNLLPDAVIRRLTRLLLAGRLRSCYRPSSDLQLSDLLHFAHSLKEMPIAITTEKPKEQH
YELPTSFFKLVLGKHLKYSCCYFSDKSMTLEDAEKAMLELYCEKSQLKDGHTVLDVGCGWGSLTLYIAQKYSNCKITGIC
NSTTQKAHIEEQCRNLQLQNVEIIVADISTYEMEASYDRIFSIEMFEHMKNYKDLLQKISKWMEEDGLLFVHHFCHKAFA
YHFEDINEDDWITRYFFTGGTMPSANLLLYFQDDVSVVNHWLVNGKHYAQTSEEWLKRMDQNLASIKPIMEGTYGKEQAV
KWTVYWRTFFIAVAELFGYNDGEEWMVALFLFKKK
>PgraNMT_onekp_QEBC_scaffold_2051261
MEGIMQLPYDVTVKMMMASLERNLLPDAVIRRLTRLLLAARLRSCYRPSSDLQLSDLLHFVHSLREMPIAIQTDKAKEQH
YELPTSFFKLVLGKHLKYSCCYFSDKSVSLDDAEKAMLELYCERAQLKDGHTVLDVGCGWGSLVLYIAQNYSNCKITGIC
NSTTQKAHIEEQCKNLQLSNVEITVGDISTFEMDASYDRIFSIEMFEHMKNYKDLLNKIAKWMKEDTLLFVHHFCHKAFA
YHFEDNNEDDWITRYFFTGGTMPAANLLLYFQDDVSIVDHWLVNGKHYAQTSEEWLKRMDRNMGSIKPIMEATYGKDSAV
KWTVYWRTFFISVAELFGYSNGDEWMVAHFLFKRKGNHSFPLHSNDPCE
>CsinNMT_XM_006474387.2_-_ORF_1_(frame_2)
MEGIMHAAYDATVRLMLSSLERNLLPDAVIRRLSRLLLGGRLRSGYKPSAELQLSDLLQFAHSLREMPIAIQTDKAKEQH
YELPTSFFKLVLGKYFKYSCCYFSDASKTLEDAEKAMLELYCERSRLEDGHTVLDVGCGWGSLSLYIAQKYSNCKITGIC
NSKTQKEFIEEQCRVLELQNVEIIVADISTFEMEASYDRIYSIEMFEHMKNYQNLLKKISKWMKEDTLLFVHHFCHKTFA
YHFEDTNDDDWITKYFFTGGTMPSANLLLYFQDDVSVVDHWLVNGKHYAQTSEEWLKRMDNNLASIKPIMESTYGKDQAV
KWTVYWRTFFIAVAELFGYNNGEEWMVTHFLFRKKME
>AnegNMT_onekp_VFFP_scaffold_2044829
MEGIMHAPYDATVRLMLSSLERNLLPDAVIRRLTRFLLSSRLRSCYKPSSQLQLSDLLHFVHSLKEMPIAIETEKPKSQH
YELPISFFKLVLGENLKYSCCYFSDNSSTLEDAEKAMFELYCERCQLKDGHSVLDIGCGWGSLALYIAQKYSNCKVTGIC
NSRTQKAHIEERCRVLELQNVEIIVADISTFEMEASYDRIFSIEMFEHMKNYQDLLKKVSNWMKQDSLLFVHHFCHKTFA
YHFEETNEDDWITRYFFSGGTMPSANLLLYFQDDVSVADHWLINGKHYAQTSEEWLKRMDQNVASIKPIMESTYGNDQAA
KWIVYWRTFFIAVAELFGYNDGEEWMVALFLFKKKI
>LchiNMT_onekp_WAXR_scaffold_2007445
MSQAHTDVHVAPDKCVSSHITQTHISNPTNSSLSSMETIMQAPYDATVRLMLASLERNLLPDVVIRRLTRLLLASRLRSC
YEPSSQLQLAHLLHFVHSLKEMPIAIETDKPKSQHYELPISFFKLVLGQNLKYSCCYFSDKSSTLEDAEKEMLELYCERC
QLKDGHTVLDVGCGWGSLSLYIAQKYSNCKITGICNSRTQKTYIEEQCRVLEIQNVEIIQADISTFEMEASYDRIFSIEM
FEHMKNYQDLLKKISSWMKQDSLLFVHHFCHKTFAYHYEVTNEDDWITKYFFSGGTMPSANLLLYFQDDVSVVDHWLVNG
KHYAQTSEEWLKRMDQNMASIKPIMESTYGKDQAVKWTVYWRTFFIAVAELFGYSEGEEWMVTHFLFKKKN
>PharNMT_onekp_OBTI_scaffold_2010126
MEGIIQASYGATVKVMLASLERNMLPDAVIRRLTRLLLASRLRSGYKPSSELQLSDLLHFVHSLKEMPIAIKTDEPKSQH
YELPTSFFKLVLGQNFKYSCCYFSDESSTLEDAEKQMFELYCDRSQLKDGHSVLDIGCGWGSLCLYIAQKYSSCRITGIC
NSKTQKAYIEEQCRVLQLQNVEIIVADISTFQMEGSYDRIYSIEMFEHMKNYQDLLKKISKWMKEDSLLFVHHFCHKVFA
YHFEDINEDDWITRYFFTGGTMPAANLLLYFQDDVSVVDHWLVNGKHYAQTSEEWLKRMDKNKSSIKPIMELTYGKDQAV
KWTVYWRTFFIAVAELFGYNNGEEWMVALFLFKRK
>HcanNMT_onekp_OLXF_scaffold_2091453
MESLTQAPYAATVRLMLTSLERNLLPDAVVRRLTRFLLASRLRSGYRPSAELQLSDLLHFAHSLREMPIAIKTDEPKTQH
YELPTSFFKLVLGNNFKYSCCYFSDGSTTLEAAEEAMLELYCERSQLKDGHTVLDVGCGWGSLSLYIARKYPNCRVTGIC
NSTTQKAFIEEQCRDGQLQNVEIIVADISTFEMEASFDRIYSIEMFEHMKNYQDLLKKISKWMNQDSLLFVHYFCHKAFA
YHFEDINEDDWITRYFFTGGTMPSANLLLYFQDDVSVVNHWLVNGKHYAQTSEEWLKRMDKNLAAIKPIMESTYGKDQAV
KWTVYWRTFFIAVAELFGYNDGEEWMVALFLFKKK
>GhirNMT_XM_016834230.1_-_ORF_1_(frame_3)
MEGLIQVPYDVTVRFMLTSLERNLLPDAIIRRLTRLLLASRLRSGYKPSTELQLSDLLQFAHSLKEMPIAIKTDKPKTQH
YELPTSFFKLVLGKNFKYSCCYFSDESKTLEDAEEAMLELYCERSQLKDGQTVLDVGCGWGSLSLYIARKYPNCRVTGIC
NSTTQKAFIEEQYRDRQLQNVEIIVADISTFEMEASYDRIYSIEMFEHMKNYHDLLKKISKWMKEDSLLFVHYFCHKAFA
YHFEDINEDDWITRYFFTGGTMPSANLLLYFQDDVSVVNHWFVNGKHYAKTSEEWLKRMDRSLASIKPIMESTYGKDQAV
KWTVYWRTFFIAVAELFGYNNGEEWMVALFLFKKK
>TcacNMT_XM_018127146.1_-_ORF_1_(frame_3)
MMQAPYDATVRLMLASLERNLLPDAVIRRLTRLLLASRLRSGYRPSIELQLSDLLQFAHSLKEMPIAIKTDKPKTQHYEL
PTSFFKLVLGKNFKYSCCYFSDGSRTLEDAEEAMLELYCERSQLKDGHTVLDVGCGWGSLSLHIARKYPNCRVTGICNST
TQKAFIEEQCRDHQLQNVEIIVADISSFEMEASYDRIYSIEMFEHMKNYQDLLKTLSKWMKQDSLLFVHYFCHKAFAYHF
EDINEDDWITRYFFTGGTMPSANLLLYFQDDVSVVNHWLVNGKHYAQTSEEWLKRMDQNLASIKPIMESTYGKDQAVKWT
VYWRTFFIAVAELFGYNNGEEWMVALFLFKKK
>DzibNMT_XM_022898959.1_-_ORF_1_(frame_2)

MSQAKRLLMEGLMQAPYDATVRLMLASLERNLLPDAVIRRLTRLLLASRLRSGYRPSTELQLSDLLHFAHSLRDMPIAIK
TDKPKAQHYELPASFFKLVLGKNFKYSCCYFSDGSKTLEDAEEAMLELYCERSQLKDGHTVLDVGCGWGSLSLYIARKYP
NCMVTGICNSITQKAFIEEQCRDQQLQNVDIIVADISTFEMEASFDRIYSIEMFEHMKNYQDLLKKLSKWMKQDSLLFVH
YFCHKAFAYHFEDINEDDWITRYFFTGGTMPSANLLLYFQDDVSIVNHWLVNGKHYAQTSEEWLKKMDQNLASIKPIMES
TYGKEQAVKWAVYWRTFFIAVAELFGYNNGEEWMVALFLFKKK
>BnapNMT_XM_013847818.2_-_ORF_1_(frame_1)
MERIVEVAYGASVKSVLTLLEKNLLPDVVIRLLTRLLLAGRLRSGYKPTAELQLSDLLRFVNSIKEMPIAINTEKPKTQH
YELPTAFFELVLGRNIKYSSCYFPKDSSSLEEAEEAILALYCERANVEDGQSVLDVGCGWGSLSLYIARKYSNCKLTGLC
NSKTQKAFIDDQCRLIGIQNVEIIVGDISTFEHEGTYDRVFSIEMFEHMKNYGELLKKIGSWMKEDSLLFVHYFCHKTFA
YHFEDVRDDDWITRYFFSGGTMPSANLLLYFQDDVSIVDHWLLNGKHYARTSEEWLKRMDKEIVAIKEIMKMTYGKEEAV
KWMVYWRTFFMAVAELFGYNNGEEWMISHFLFKKK
>BrapNMT_XM_009126777.2_-_ORF_1_(frame_1)
METIVKVAYDASVKTVLTLLEKNLLPDVVIRRLTRLLLAGRLRSGYKPTAELQLSDLLRFVNSIKEMPIAINTEKPKTQH
YELPTAFFELVLGRNMKYSSCYFSNDSSSLEDAEEAMLALYCQRAKVEDGQSVLDVGCGWGSLSLYIARKYSNCKITGLC
NSKTQKAFIDEKCRKRGIQNIEIIVGDISTFEHEGTYDRVLSIEMFEHMKNYGELLKKIGRWMKEDALLFVHHFCHKTFA
YHFEDVNDDDWITRHFFSGGTMPSANLLLYFQEDVTIVDHWLLNGNHYAKTSEEWLKRMDKEIVAIKEVMEMTYGKEEAV
KGMVYWRTFFIAVAELFGYSNGEEWMVSHFLFKKK
>RodoNMT_onekp_SWPE_scaffold_2016676
MITQLLYDPTVKVFLASLERNLLPDAVVRRLTRLLLDGRLRSGYKPTAELQHSDLLNFVQSLKEMPIAVKTDDAKIQHYE
LPTSFFKLVLGDNRKYSCCYFKNDSSTLEEAEKAMMELYCERVQLQDGQTVLDVGCGWGSLSLYIAQKYKNCKITGICNS
KTQKQFIDEQCRNLEMENIEIIVADITTFEMEGVYDRVFSIEMFEHMKNYGKLLKKISKWMKEDSLLFVHHFCHKTFSYH
FEDINEDDWITRYFFSGGTMPSANLLLYFQEDVSVVDHWLLNGKHYAQTSEEWLKRMDKNLTLIKPIMESTYGADQAVKW
TAYWRTFFISVAELFGYNNGEEW
>AthaNMT1_AY136408.1_-_ORF_1_(frame_2)
MEKIIDVAYGASVKVGLTLLEMNLLPDIVIRRLTRLLLAGRLRSGYKPTAEMQLSDLLRFVDSIKKMPIAIHTEKPKTQH
YELPTAFFELVLGRNMKYSSCYFSNDSSSLEDAEEAILALYCERAKVEDGQSVLDIGCGWGSLSLYIARKYSKCKLTGIC
NSKTQKAFIDEKCRKLGLQNIEIIVADISTFEHEGTYDRIFSIEMFEHMKNYGELLKKIGKWMKEDSLLFVHYFCHKTFA
YHFEDVNDDDWITRHFFSGGTMPSADLLLYFQENVSIVDHWLVSGTHYAKTSEEWLKRMDKEIVAVKKIMEVTYGKEEAV
KWMVYWRTFFIAVAELFGYNNGEEWMVAHFLFKKK
>AthaNMT2_BT005878.1_-_ORF_1_(frame_1)
MEKIIDVAYGASVKAGLTLLEKNLLPDLVIRRLIRLLLASRLRSVYKPTAEMQLSDLLRFVDSLKKMPIAINTETAKTQH
YELPTAFFEHALGRNIKYSCCYYSNDSSSLEEAGEAMLALSCERAKVEDGQSVLDIGCGWGSLTLYIARKYSKCKLTGIC
NSKTQKAFIDEKCRKLGLQNVEIIAADISTFEHEGTYDRIFSIEMFEHMKNYGELLKKIGNWMKEDSLLFVHYLCHKTYA
YHFEDVNDDDWITRHFFSGGTMPSADLLLYFQENVSIMDHWLVNGTHYAKTSEEWLKGMDKEIVAVKEIMEVTYGKEEAV
KWTVYWRTFFIALAELFAYNNGDEWMIAHFLFKKK
