# Supplementary material for: Molecular Origins of Functional Diversity in Benzylisoquinoline Alkaloid Methyltransferases
Source: Front Plant Sci. 2019 Aug 30;10:1058. doi: 10.3389/fpls.2019.01058 (PMC6730481; doi:10.3389/fpls.2019.01058)
Supplement: Supplementary file 2 [file Table_1.pdf]

Supplementary Table 1. *O*- and *N*-methyltransferases show to accept BIA substrates in vitro, which have been characterized at the molecular level. Specificity refers to regiospecificity (2,6,7,9,3',4'), stereospecificity [(R) or (S)] and to the number of pre-existing bonds on the nitrogen atom (2'*N*, 3'*N*). † represents features not conclusively demonstrated, ‡ represents activities that have been demonstrated but are quite minor or weak, "*nd*" indicates that no activities have been detected and "not tested" indicates that no *in vitro* assays were performed. Positions denoted by numbering scheme are presented visually in Supplementary Figures 4 and 5. For enzymes with many reported substrates, minor activities are not listed.

Plant species	Short name	Enzyme full name	Genbank ID	*In vitro* substrate(s)	Specificity	Functional evidence *in planta*	Reference(s)
Glaucium flavum	GflOMT1	*O*-methyltransferase 1	KP176693	(*R,S*)-Norlaudnosoline<br>(*R,S*)-6-*O*-Methylnorlaudnosoline<br>(*R,S*)-6-*O*-Methylaudnosoline<br>(*R,S*)-Tetrahydrocolumbamine<br>(*S*)-Reticuline<br>(*R,S*)-Scoulerine	6,7,3',4'	High expression in stem & flower bud correlating with accumulation of glaucine	Chang *et al*., 2015
	GflOMT2	*O*-methyltransferase 2	KP176694	(R,S)-Norlaudnosoline<br>(R,S)-Scoulerine<br>(R,S)-Tetrahydrocolumbamine<br>(S)-Reticuline	6,3',4'	High expression in root tissue	Chang *et al*., 2015
	GflOMT3	*O*-methyltransferase 3	KP176695	*nd*		Very low expression in all tissues	Chang *et al*., 2015
	GflOMT4	*O*-methyltransferase 4	KP176696	*nd*		Moderate expression level in all tissues examined	Chang *et al*., 2015
	GflOMT5	*O*-methyltransferase 5	KP176697	*nd*		Moderate expression level in stem tissue, all others low	Chang *et al*., 2015
	GflOMT6	*O*-methyltransferase 6	KP176698	(*R,S*)-Scoulerine<br>(*S*)-Reticuline<br>(*R,S*)-Tetrahydrocolumbamine<br>(*R,S*)-Norreticuline	2,9<br><br>7,3'	High expression in root, leaf and flower bud tissues	Chang *et al*., 2015
	GflOMT7	*O*-methyltransferase 7	KP176699	(*R,S*)-Scoulerine<br>(*R,S*)-Tetrahydrocolumbamine	2,9	Moderate expression level in root, stem and flower bud tissues	Chang *et al*., 2015
	GflNMT1	Coclaurine *N*-methyltransferase		(*S*)-Coclaurine<br>Heliamine	2'N		Hagel *et al*., 2015b
	GflNMT2 (GfTNMT)	Tetrahydroprotoberberine *N*-methyltransferase		(*R,S*)-Stylopine<br>(*S*)-Canadine			Hagel *et al*., 2015b
	GflNMT3	*N*-methyltransferase 3		(*S*)-Reticuline<br>(*R,S*)-Stylopine	3'N		Hagel *et al*., 2015b
	GflNMT4	*N*-methyltransferase 4		(*S*)-Coclaurine<br>(*S*)-Reticuline	2'N, 3'*N*		Hagel *et al*., 2015b
	GflNMT5	*N*-methyltransferase 5		(*S*)-Reticuline<br>(*S*)-Coclaurine	3'N, 2'*N*‡		Hagel *et al*., 2015b
	GflNMT6	*N*-methyltransferase 6		(*S*)-Coclaurine	2'*N*		Hagel *et al*., 2015b
Papaver somniferum	Ps6OMT (PsOMT2)	Norcoclaurine 6-*O*-methyltransferase	AY217335, AY268894	(*R,S*)-Norcoclaurine<br>(*R,S*)-Norprotosinomenine<br>Isorientaline	6,7	Majority of BIAs content reduced by VIGS knockdown	Facchini & Park, 2003; Ounaroon *et al*., 2003; Desgagné-Penix & Facchini, 2012
	Ps7OMT (PsOMT1)	Reticuline 7-*O*-methyltransferase	AY268893	(*R,S*)-Reticuline<br>(*R,S*)-Orientaline<br>(*R,S*)-Protosinomenine<br>(*R,S*)-Isoorientaline	6,7,4'<br>*S* > *R*	*N*-methylated BIAs (eg. laudanine) content reduced by VIGS knockdown; Morphinans and noscapine content increased by VIGS knockdown	Ounaroon *et al*., 2003; Desgagné-Penix & Facchini, 2012
	PsSOMT1	Scoulerine-9-*O*-methyltransferase	JN185323	(*S*)-Scoulerine<br>(*S*)-Norreticuline<br>(*S*)-Reticuline	9,2<br><br>3',7	Noscapine, papaverine and pathway intermediates decreased by VIGS knockdown; Transcript abundance correlates with noscapine accumulation in cultivars	Dang & Facchini, 2012
	PsSOMT2	*O*-methyltransferase 2	JN185324	(S)-Scoulerine	9	Noscapine decreased by VIGS knockdown; Transcript abundance correlates with noscapine accumulation in cultivars	Dang & Facchini, 2012
	PsSOMT3	*O*-methyltransferase 3	JN185325	(S)-Scoulerine	9	Noscapine decreased by VIGS knockdown; Transcript abundance correlates with noscapine accumulation in cultivars	Dang & Facchini, 2012
	PsN7OMT	Norreticuline 7-*O*-methyltransferase	FJ156103, XM_02657 5469	(S)-Norreticuline	7	Transcript abundance correlates with papaverine accumulation in cultivars; Papaverine content reduced by VIGS knockdown	Pienkny *et al*., 2009; Desgagné-Penix & Facchini, 2012
	Ps4'OMT1	3'-hydroxy-*N*-methylcoclaurine 4'-*O*-methyltransferase 1	AY217333	*Not tested*			Facchini & Park, 2003

Supplementary Table 1 (*continued)*

Plant species	Short name	Enzyme full name	Genbank ID	*In vitro* Substrate(s)	Specificity	Functional evidence *in planta*	Reference(s)
Papaver somniferum (continued)	Ps4'OMT2	3'-hydroxy-*N*-methylcoclaurine 4'-O-methyltransferase 2	AY217334	(*R,S*)-3'-hydroxy-*N*-methylcanadine<br>(*R,S*)-laudanosoline<br>(*R,S*)-6-*O*-methylnorlaudnosoline<br>(*R,S*)-norlaudanosoline	4'[†]	Expression level correlates with known BIA biosynthesis genes 4'-*O*-methylcoclaurine and majority of BIAs content reduced by VIGS knockdown	Facchini & Park, 2003; Ziegler *et al.,* 2005; Desgagné-Penix & Facchini, 2012
	PsOMT2: PsOMT3 dimer	4'-O-desmethyl-3-O-acetylpapaveroxine 4'-*O*-methyltransferase	JN185324, JN185325	4'-*O*-desmethyl-3-*O*-acetylpapaveroxine<br>Narcotoline	4'	Narcotoline content increased by VIGS knockdown of OMT2; High narcotoline content in cultivar expressing inactive mutants OMT2b and OMT3b; Co-expression in yeast enables noscapine biosynthesis	Park *et al*., 2018; Li & Smolke, 2016
	PsOMT2: Ps6OMT dimer	4'-O-desmethyl-3-O-acetylpapaveroxine 4'-*O*-methyltransferase	JN185324, AY217335	4'-*O*-desmethyl-3-*O*-acetylpapaveroxine<br>Narcotoline	4'	Narcotoline content increased by VIGS knockdown of OMT2; High narcotoline content in cultivar expressing inactive mutants OMT2b and OMT3b; Co-expression in yeast enables noscapine biosynthesis	Park *et al*., 2018; Li & Smolke, 2016
	PsOMT2b: PsOMT3b dimer		MH029293, MH029295	*nd*			Park *et al*., 2018
	PsCNMT	Coclaurine *N*-methyltransferase	AY217336	*Not tested*		N-desmethylated BIA content increased by VIGS knockdown; Induction following elicitor treatment correlates with known BIA biosynthesis enzymes; Expression in yeast enables BIA biosynthesis (various)	Facchini & Park, 2003; Desgagné-Penix & Facchini, 2012; Fossati *et al*., 2014; Trenchard & Smolke, 2015; Li & Smolke, 2016
	PsTNMT	Tetrahydroprotoberberine *N*-methyltransferase	Q108P1	(*R,S*)-Canadine<br>(*R,S*)-Stylopine<br>(*R,S*)-Tetrahydropalmatine		Induction following elicitor treatment correlates with known BIA biosynthesis enzymes; Protein level correlates with protoberberine accumulation in *P. somniferum* tissues	Liscombe & Facchini, 2007
	PsRNMT	Reticuline *N*-methyltransferase	KX369612	(*R*)- and (*S*)-Reticuline<br>(*S*)-Bulbocapnine<br>(*S*)-Corytuberine<br>(*S*)-Glaucine<br>Tetrahydropapaverine<br>Noscapine	*R > S*	Magnoflorine content reduced and corytuberine content increased by VIGS knockdown	Morris & Facchini, 2016
	PsNMT4		KX369613	*nd*			Morris & Facchini, 2016
Coptis japonica	Cj6OMT	Norcoclaurine 6-*O*-methyltransferase	D29811	(*S*)- and (*R*)-Norcoclaurine<br>(*R,S*)-Norlaudanosoline<br>(*R,S*)-Laudanosoline<br>(*S*)-Scoulerine<br>(*S*)- and (*R*)-Coclaurine	6[†]	Overexpression in *E. californica* cells substantially increases BIA content	Morishige *et al*., 2000; Tamura *et al*., 2006
	Cj4'OMT	3'-hydroxy-N-methyl-coclaurine 4'-*O*-methyltransferase	D29812	(*R,S*)-Laudnosoline<br>(*R,S*)-Norlaudnosoline<br>(*R,S*)-6-*O*-Methylnorlaudnosoline<br>(*S*)- and (*R,S*)-Scoulerine<br>(*R,S*)-Reticuline	4[†]		Morishige *et al*., 2000
	CjSOMT (CjSMT)	Scoulerine 9-*O*-methyltransferase	D29809	(*S*)- and (*R,S*)-Scoulerine<br>(*R,S*)-Tetrahydroxyprotoberberine<br>(*R,S*)-Norreticuline<br>(*R,S*)-norlaudanosoline	9[†]<br>7 or 3' [†]	Overexpression in *E. californica* cells enhanced production of *O*-methylated protoberberines	Takeshita *et al*, 1995; Morishige *et al*, 2000; Takemura *et al*, 2010
	CjCoOMT	Columbamine *O*-methyltransferase	AB073908	Columbamine<br>(*S*)- and (*R,S*)-Scoulerine<br>(*R,S*)-Tetrahydroxyprotoberberine	2, other		Morishige *et al*., 2002
	CjCNMT	Coclaurine *N*-methyltransferase	AB061863	(*R*)- and (*S*)-Coclaurine<br>6,7-Dimethoxyl-1,2,3,4-tetrahydroisoquinoline<br>(*R,S*)-Noreticuline<br>(*R,S*)-6-*O*-Methylnorlaudanosoline<br>(*R,S*)-Norlaudanosoline	2'*N*, 3'*N*[‡]	Expression in *E. coli* enables reticuline biosynthesis; Expression in yeast enables magnoflorine biosynthesis	Choi *et al*., 2002; Minami *et al*.,2008
Coptis chinensis	Cc6OMT1	6-*O*-methyltransferase 1	MH165875	(*S*)-Norcoclaurine	6, 7	Transcript abundance correlates with alkaloid accumulation in tissues	He *et al*., 2018
	Cc6OMT2	6-*O*-methyltransferase 2	MH165876	(*S*)-Norcoclaurine	6, 7	Transcript abundance correlates with alkaloid accumulation in tissues	He *et al*., 2018

Supplementary Table 1 (*continued*)

Plant species	Short name	Enzyme full name	Genbank ID	*In vitro* Substrate(s)	Specificity	Functional evidence *in planta*	Reference(s)
Coptis teeta	Ct6/7OMT	Norcoclaurine 7-*O*-methyltransferase	MH165877	(*S*)-Norcoclaurine	6,7		He *et al*., 2018
	CtSOMT	Scoulerine 9-*O*-methyltransferase	MH165874	(*S*)-Scoulerine	9	Transcript abundance correlates with alkaloid accumulation in tissues	He *et al*., 2018
Eschscholzia californica	Ec7OMT	Reticuline 7-*O*-methyltransferase	AB232153	(*S*)-Reticuline<br>(*S*)-Norreticuline<br>(*S*)-Scoulerine<br>(*S*)-6-*O*-Methylnorlaudanosoline<br>(*S*)-Laudanosoline	7<br>7,3'<br>2		Fujii *et al*., 2007; Purwanto *et al*., 2017
	EcS/ROMT (G3OMT)	Scoulerine/reticuline *O*-methyltransferase	LC171865	(*S*)-Reticuline<br>(*S*)-Norreticuline<br>(*S*)-Scoulerine	7,3'<br>9,2	Expression in yeast enables biosynthesis of chelerythrine; Highly expressed in high macarpine *E. californica* cell line A5-1	Purwanto *et al.,* 2017
	EcG2OMT		LC171866	*not tested*			Purwanto *et al*., 2017
	EcG11OMT	10-hydroxy-dihydrosanguinarine 10-*O*-methyltranferase	LC171864	10-Hydroxydihydrosanguinarine[†]	10[†]		Purwanto *et al*., 2017; Purwanto, 2017
	EcTNMT	Tetrahydroprotoberberine *N*-methyltransferase	EU882977	(*R,S*)-Stylopine<br>(*R,S*)-Tetrahydropalmatine<br>(*R,S*)-Canadine<br>(*S*)-Scoulerine		Transcript expression induced by elicitor treatment of cell culture	Liscombe *et al*., 2009
Thalictrum flavum	Tf6OMT	Norcoclaurine 6-O-methyltransferase	AY610507	Norlaudnosoline		Cellular localization of transcripts correlates with known BIA genes in berberine accumulating organs	Samanani *et al*., 2005; Robin *et al*., 2016
	Tf4'OMT	3'-hydroxy-N-methylcoclaurine 4'-*O*-methyltransferase	AY610510	*Not tested*		Cellular localization of transcripts correlates with known BIA genes in berberine accumulating organs	Samanani *et al*., 2005
	TfSOMT	Scoulerine 9-*O*-methyltransferase	AY610512	*Not tested*		Cellular localization of transcripts correlates with known BIA genes in berberine accumulating organs	Samanani *et al.,* 2005
	TfCNMT	Coclaurine *N*-methyltransferase	AY610508	(*R,S*)-Norreticuline<br>Dimethoxytetrahydroisoquinoline<br>(±)-Pavine<br>(*S*)-Scoulerine	2'*N*	Cellular localization of transcripts correlates with known BIA genes in berberine accumulating organs	Samanani *et al*., 2005; Liscombe *et al*., 2009
	TfPavNMT	Pavine *N*-methyltransferase	EU883010	(*S*)-Reticuline<br>(±)-Pavine<br>(*R,S*)-Stylopine<br>(*S*)-Scoulerine	2'*N*, 3'*N*		Liscombe *et al*., 2009; Torres *et al.,* 2016
Papaver bracteatum	PbTNMT	Tetrahydroprotoberberine *N*-methyltransferase	EU882994	(*R,S*)-Tetrahydropalmatine<br>(*R,S*)-Stylopine		Transcript expression induced by elictor treatment of cell culture	Liscombe *et al*., 2009
Psychotria ipecacuanha	IpeOMT1		AB527082	(*R,S*)-Isococlaurine<br>(*R,S*)-Norcoclaurine<br>(*R,S*)-Norprotosinomenine<br>(*R,S*)-Nororientaline	6, 4'	Not involved in BIA biosynthesis	Nomura & Kutchan, 2010
	IpeOMT2		AB527083	Oripavine	3	Not involved in BIA biosynthesis	Nomura & Kutchan, 2010
	IpeOMT3		AB527084	(*S*)-Norreticuline<br>(*R,S*)-Nororientaline<br>(*S*)-Coclaurine	7<br>*S* only	Not involved in BIA biosynthesis	Nomura & Kutchan, 2010
Thalictrum tuberosom	TtOMTII;1		AF064693	(*R,S*)-Laudanosoline<br>(*R,S*)-3'-*O*-Methylnorlaudanosoline<br>(*R,S*)-4'-*O*-Methylnorlaudanosoline<br>(*R,S*)-3-*O*-Desmethylcheilanthifoline		Not involved in BIA biosynthesis	Frick & Kutchan, 1999
	TtOMTII;2		AF064694	(*S*)-Norcoclaurine<br>(*R,S*)-4'-*O*-Methylnorlaudanosoline		Not involved in BIA biosynthesis	Frick & Kutchan, 1999
	TtOMTII;3		AF064695	(*R,S*)-Norisoorientaline		Not involved in BIA biosynthesis	Frick & Kutchan, 1999
	TtOMTII;4		AF064696	(*R,S*)-Norisoorientaline		Not involved in BIA biosynthesis	Frick & Kutchan, 1999
	Heterolog-ous dimers (various)			Many			Frick & Kutchan, 1999
Ephedra sinica	EsPaNMT	Phenylalklylamine *N*-methyltransferase	MH029305	(*S*)-Coclaurine<br>(*S*)-Reticuline<br>(*R,S*)-Stylopine		Not involved in BIA biosynthesis	Morris *et al*., 2018
Dactylicapnos scandens	DsC7OMT	Corytuberine 7-*O*-methyltransferase		(*S*)-Corytuberine	7		He *et al*., 2017

Supplementary Table 1 (*continued)*

Fossati, E., Ekins, A., Narcross, L., Zhu, Y., Falgueyret, J.-P., Beaudoin, G. A. W., et al. (2014). Reconstitution of a 10-gene pathway for synthesis of the plant alkaloid dihydrosanguinarine in *Saccharomyces cerevisiae*. *Nat. Commun.* 5, 3283. doi:10.1038/ncomms4283.

Hagel, J. M., Mandal, R., Han, B., Han, J., Dinsmore, D. R., Borchers, C. H., et al. (2015). Metabolome analysis of 20 taxonomically related benzylisoquinoline alkaloid-producing plants. *BMC Plant Biol.* 15, 220. doi:10.1186/s12870-015-0594-2.
