# Supplementary material for: Molecular Origins of Functional Diversity in Benzylisoquinoline Alkaloid Methyltransferases
Source: Front Plant Sci. 2019 Aug 30;10:1058. doi: 10.3389/fpls.2019.01058 (PMC6730481; doi:10.3389/fpls.2019.01058)
Supplement: Supplementary file 3 [file Image_1.pdf]

	6OMTs								4'OMTs											7OMTs							Scoulerine OMTs					
	Tf6OMT	Cj6OMT	Cc6OMT2	Ps6OMT	GFLOMT2	PsSOMT3	PsOMT3b	PsN7OMT	Cj4'OMT	Tf4'OMT	GFLOMT1	Ps4'OMT1	Ps4'OMT2	CjCoOMT	Cc6OMT1	GflOMT4	PsSOMT2	PsOMT2b	EcG11OMT	Ct67OMT	GFLOMT3	Ps7OMT	Ec7OMT	EcSROMT	GFLOMT5	EcG2OMT	GFLOMT7	PsSOMT1	GFLOMT6	TfSOMT	CjSOMT	CtSOMT
Tf6OMT	100	85	85	66	68	62	62	57	52	51	52	48	49	37	43	44	39	39	37	41	41	40	39	40	35	36	35	31	33	31	31	31
Cj6OMT		100	98	69	72	63	63	59	52	51	52	49	50	39	45	44	42	42	38	43	42	41	38	40	35	36	35	33	33	32	31	32
Cc6OMT2			100	69	71	63	63	59	52	51	53	50	51	39	45	44	41	41	37	43	41	40	38	40	36	36	36	32	34	33	32	32
Ps6OMT				100	82	81	81	71	56	56	53	51	52	40	44	45	42	42	38	43	41	40	38	41	33	34	36	32	31	31	31	31
GFLOMT2					100	73	73	69	56	55	55	52	53	39	44	46	42	42	41	43	40	41	40	42	33	35	35	33	32	33	33	33
PsSOMT3						100	99	65	52	52	49	47	48	35	38	40	37	37	38	39	38	38	36	38	31	33	33	29	28	27	27	27
PsOMT3b							100	65	52	52	49	47	47	35	38	40	38	38	38	38	38	37	36	38	31	32	33	29	28	27	27	27
PsN7OMT								100	49	50	45	42	45	37	41	39	35	35	36	40	37	38	36	39	31	32	33	29	31	32	32	32
Cj4'OMT									100	89	72	63	68	36	41	40	36	36	37	40	37	37	38	37	35	36	35	32	31	33	32	33
Tf4'OMT										100	71	64	67	37	42	42	38	38	38	42	39	39	39	39	35	37	34	31	31	35	34	35
GFLOMT1											100	73	78	34	43	43	41	41	34	40	37	37	36	36	36	35	34	29	28	30	29	29
Ps4'OMT1												100	81	36	43	42	39	39	35	37	36	37	37	38	33	32	31	29	29	30	30	30
Ps4'OMT2													100	36	44	42	40	40	35	40	37	38	36	37	35	34	32	30	29	31	30	32
CjCoOMT														100	38	38	37	37	37	35	32	36	33	34	25	26	28	26	28	25	26	26
Cc6OMT1															100	61	53	53	36	39	37	39	39	38	32	31	33	31	31	30	30	31
GflOMT4																100	59	59	35	40	39	40	38	40	32	32	31	32	29	29	29	30
PsSOMT2																	100	99.7	31	37	37	36	35	37	29	30	29	29	28	27	26	27
PsOMT2b																		100	31	37	37	36	35	37	29	30	29	29	28	27	26	27
EcG11OMT																			100	38	36	39	38	41	32	30	30	28	29	29	28	28
Ct67OMT																				100	58	54	54	64	38	37	41	38	38	36	36	36
GFLOMT3																					100	64	54	59	36	36	36	33	34	35	34	35
Ps7OMT																						100	53	57	37	37	39	33	33	32	33	33
Ec7OMT																							100	63	37	36	36	33	33	33	32	33
EcSROMT																								100	40	38	39	38	37	36	36	37
GFLOMT5																									100	82	77	40	43	43	43	45
EcG2OMT																										100	75	40	42	44	43	45
GFLOMT7																											100	42	44	46	45	47
PsSOMT1																												100	63	61	60	64
GFLOMT6																													100	67	68	68
TfSOMT																														100	89	88
CjSOMT																															100	98
CtSOMT																																100

Supplementary Figure 1. Percent identity matrix for amino acid sequences of BIA *O*-methyltransferases characterized at the molecular level. Protein sequences were obtained by translation of nucleotide sequences deposited in Genbank (accession numbers provided in Supplementary Table 1) and aligned using Clustal Omega under default parameters (Chojnacki et al., 2017). Percentage identities are shaded with from green (maximum, 100%) to minimum (red, 25%).

Chojnacki, S., Cowley, A., Lee, J., Foix, A., and Lopez, R. (2017). Programmatic access to bioinformatics tools from EMBL-EBI update: 2017. *Nucleic Acids Res.* 45, W550–W553. doi:10.1093/nar/gkx273.
