# Supplementary material for: Molecular Origins of Functional Diversity in Benzylisoquinoline Alkaloid Methyltransferases
Source: Front Plant Sci. 2019 Aug 30;10:1058. doi: 10.3389/fpls.2019.01058 (PMC6730481; doi:10.3389/fpls.2019.01058)
Supplement: Supplementary file 4 [file Image_2.pdf]

	CNMT-like						RNMT-like			TNMT-like					Inactive
	CjCNMT	TfCNMT	GFLNMT6	GFLNMT5	GFLNMT1	PsCNMT	TfPavNMT	PsRNMT	GFLNMT4	GFLNMT2	PsTNMT	PbTNMT	EcTNMT	GFLNMT3	PsNMT4
CjCNMT	100	86	76	62	65	62	59	49	59	49	46	45	47	49	48
TfCNMT		100	74	60	65	61	58	49	56	50	47	46	48	49	49
GFLNMT6			100	62	66	60	60	51	59	53	47	46	49	50	47
GFLNMT5				100	65	61	59	51	53	49	46	45	49	52	46
GFLNMT1					100	78	61	56	59	51	49	49	48	53	53
PsCNMT						100	56	52	54	47	49	49	47	48	49
TfPavNMT							100	56	60	52	48	48	51	53	48
PsRNMT								100	59	48	44	44	46	50	46
GFLNMT4									100	49	47	46	45	51	46
GFLNMT2										100	81	81	83	72	64
PsTNMT											100	98	77	68	61
PbTNMT												100	77	68	60
EcTNMT													100	72	62
GFLNMT3														100	64
PsNMT4															100

Supplementary Figure 2. Percent identity matrix for amino acid sequences of BIA *N*-methyltransferases characterized at the molecular level. Protein sequences were obtained by translation of nucleotide sequences deposited in Genbank (accession numbers provided in Supplementary Table 1) and aligned using Clustal Omega under default parameters (Chojnacki et al., 2017). Percentage identities are shaded with from green (maximum, 100%) to minimum (red, 44%). GFLNMT3 shows both TNMT-like and RNMT-like properties. GFLNMT4 shows both RNMT-like and CNMT-like properties.

Chojnacki, S., Cowley, A., Lee, J., Foix, A., and Lopez, R. (2017). Programmatic access to bioinformatics tools from EMBL-EBI update: 2017. *Nucleic Acids Res.* 45, W550–W553. doi:10.1093/nar/gkx273.
