# Supplementary material for: Molecular Origins of Functional Diversity in Benzylisoquinoline Alkaloid Methyltransferases
Source: Front Plant Sci. 2019 Aug 30;10:1058. doi: 10.3389/fpls.2019.01058 (PMC6730481; doi:10.3389/fpls.2019.01058)
Supplement: Supplementary file 5 [file Image_3.pdf]

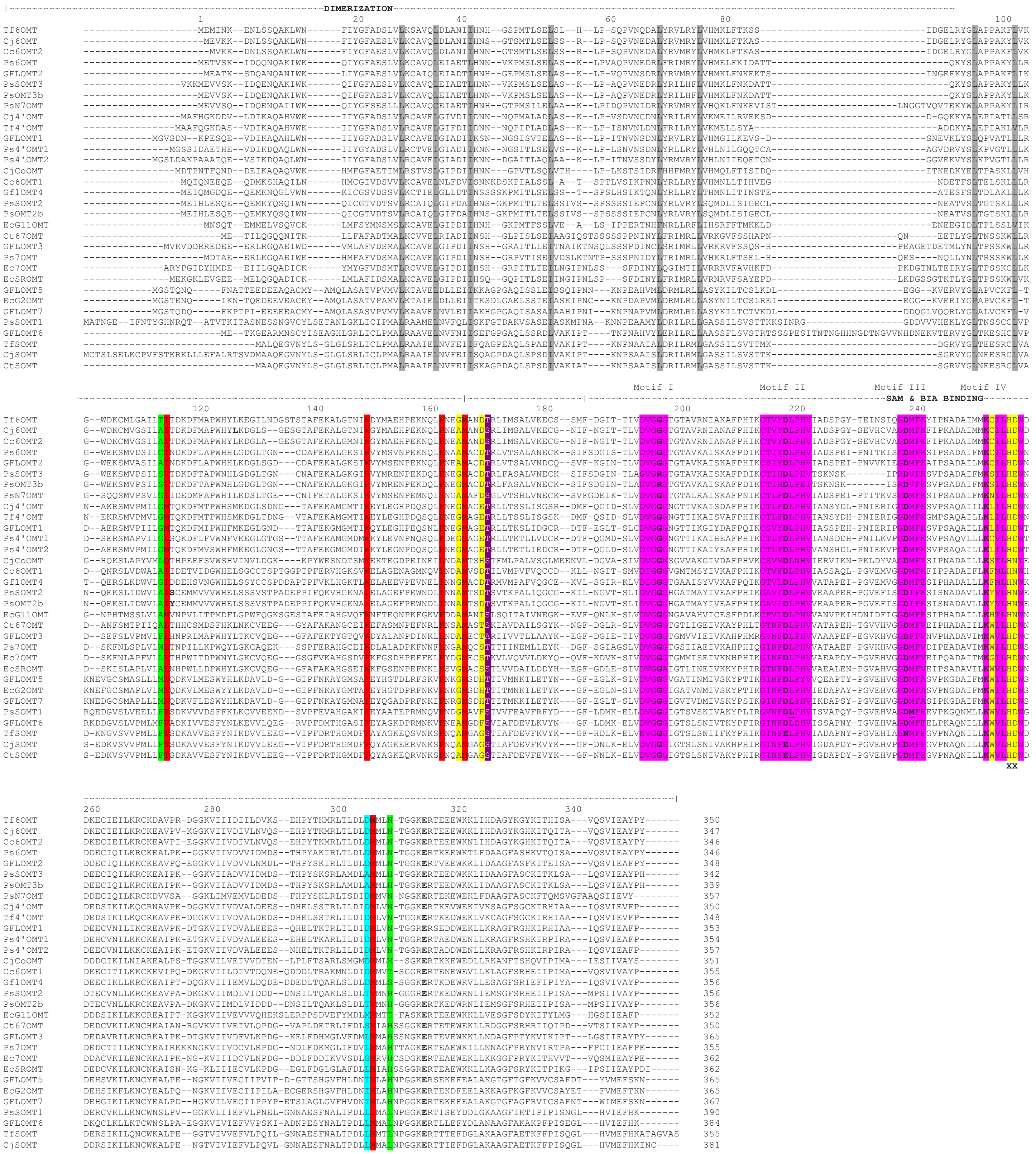

Supplementary Figure 3. Amino acid sequence alignment of BIA *O*-methyltransferases characterized *in vitro*. Protein sequences were obtained by translation of nucleotide sequences deposited in Genbank (Accession numbers listed in Supplementary Table 1) and aligned using Clustal Omega under default parameters (Chojnacki et al., 2017). Major domains and key residues are adapted from structural studies of PsSOMT1 and Tf6OMT (Cabry et al., 2019; Robin et al., 2016). Residues shaded in *grey* are highly conserved features of the dimerization domain. Residues shaded in *pink*

(*continued on next page*)

represent the canonical SAM-binding motifs, within which residues directly interacting with SAM are bolded and residues interacting with the alkaloid substrate are underlined. Residues shaded in *purple* contribute to a “closed” conformation upon SAM binding. Residues shaded in *red* form sulfur-aromatic, aromatic-aromatic or hydrophobic interactions the alkaloid substrate’s rings. Residues shaded in *yellow* form hydrogen bonds with the alkaloid substrate’s hydroxyl groups, and those interacting with the target hydroxyl group are underlined. Residues shaded in *green* are the “gatekeeper” residues proposed by Cabry et al (2019). The residue forming a hydrogen bond with the alkaloid substrate’s nitrogen atom is shaded in *cyan*. “X” indicates residues subjected to mutational analysis. Percentage identities are provided in Supplementary Figure 1.

Chojnacki, S., Cowley, A., Lee, J., Foix, A., and Lopez, R. (2017). Programmatic access to bioinformatics tools from EMBL-EBI update: 2017. *Nucleic Acids Res.* 45, W550–W553. doi:10.1093/nar/gkx273.

Cabry, M., Offen, W. A., Saleh, P., Li, Y., Winzer, T., Graham, I. A., et al. (2019). Structure of *Papaver somniferum O*-methyltransferase 1 reveals initiation of noscapine biosynthesis with implications for plant natural product methylation. *ACS Catal.* 9, 3840-3848. doi:10.1021/acscatal.9b01038.

Robin, A. Y., Giustini, C., Graindorge, M., Matringe, M., and Dumas, R. (2016). Crystal structure of norcoclaurine-6-*O*-methyltransferase a key rate-limiting step in the synthesis of benzylisoquinoline alkaloids. *Plant J.* 87, 641-653. doi:10.1111/tpj.13225.
