# Supplementary material for: Molecular Origins of Functional Diversity in Benzylisoquinoline Alkaloid Methyltransferases
Source: Front Plant Sci. 2019 Aug 30;10:1058. doi: 10.3389/fpls.2019.01058 (PMC6730481; doi:10.3389/fpls.2019.01058)
Supplement: Supplementary file 6 [file Image_4.pdf]

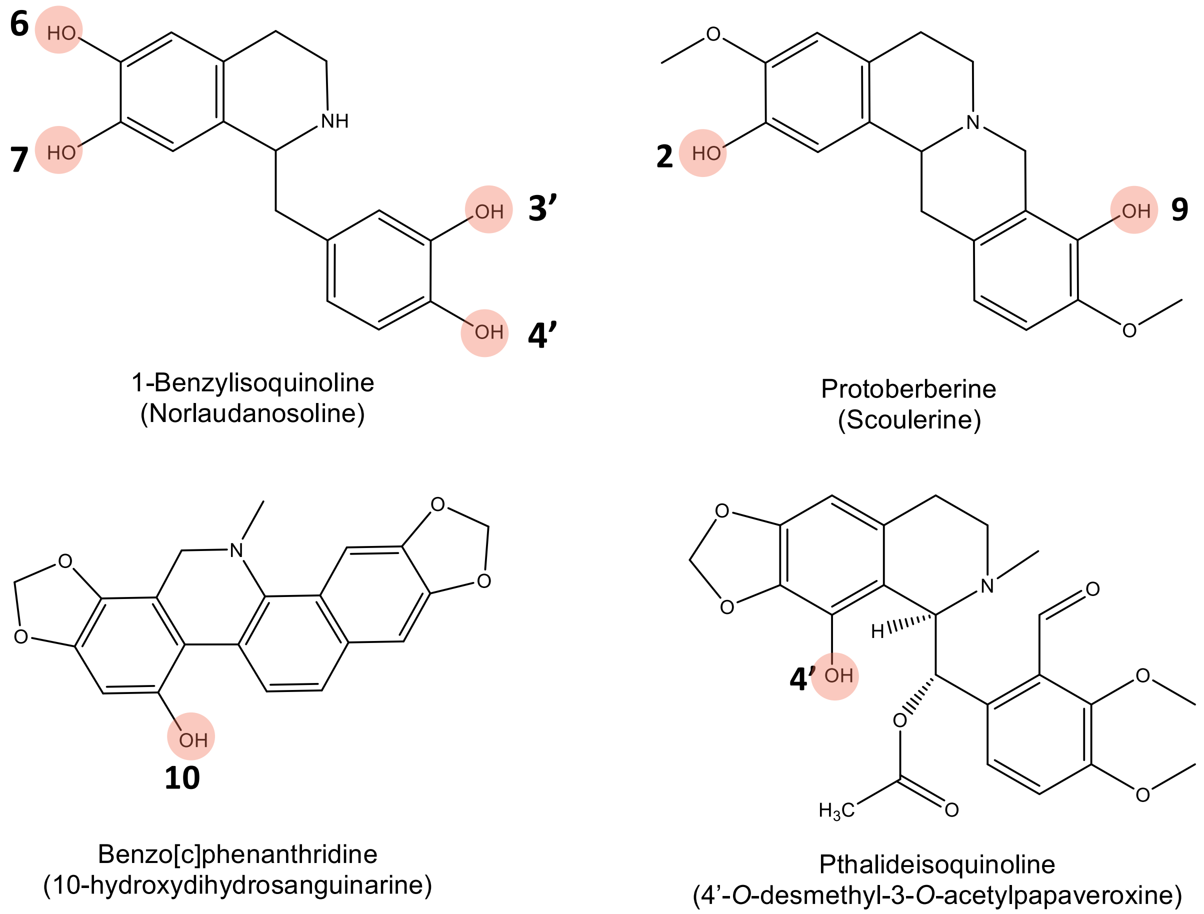

Supplementary Figure 4. Position of hydroxyl groups targeted by BIA *O*-methyltransferases. Corresponding enzymes are listed in Supplementary Table 1.
