# Supplementary material for: Molecular Origins of Functional Diversity in Benzylisoquinoline Alkaloid Methyltransferases
Source: Front Plant Sci. 2019 Aug 30;10:1058. doi: 10.3389/fpls.2019.01058 (PMC6730481; doi:10.3389/fpls.2019.01058)
Supplement: Supplementary file 7 [file Image_5.pdf]

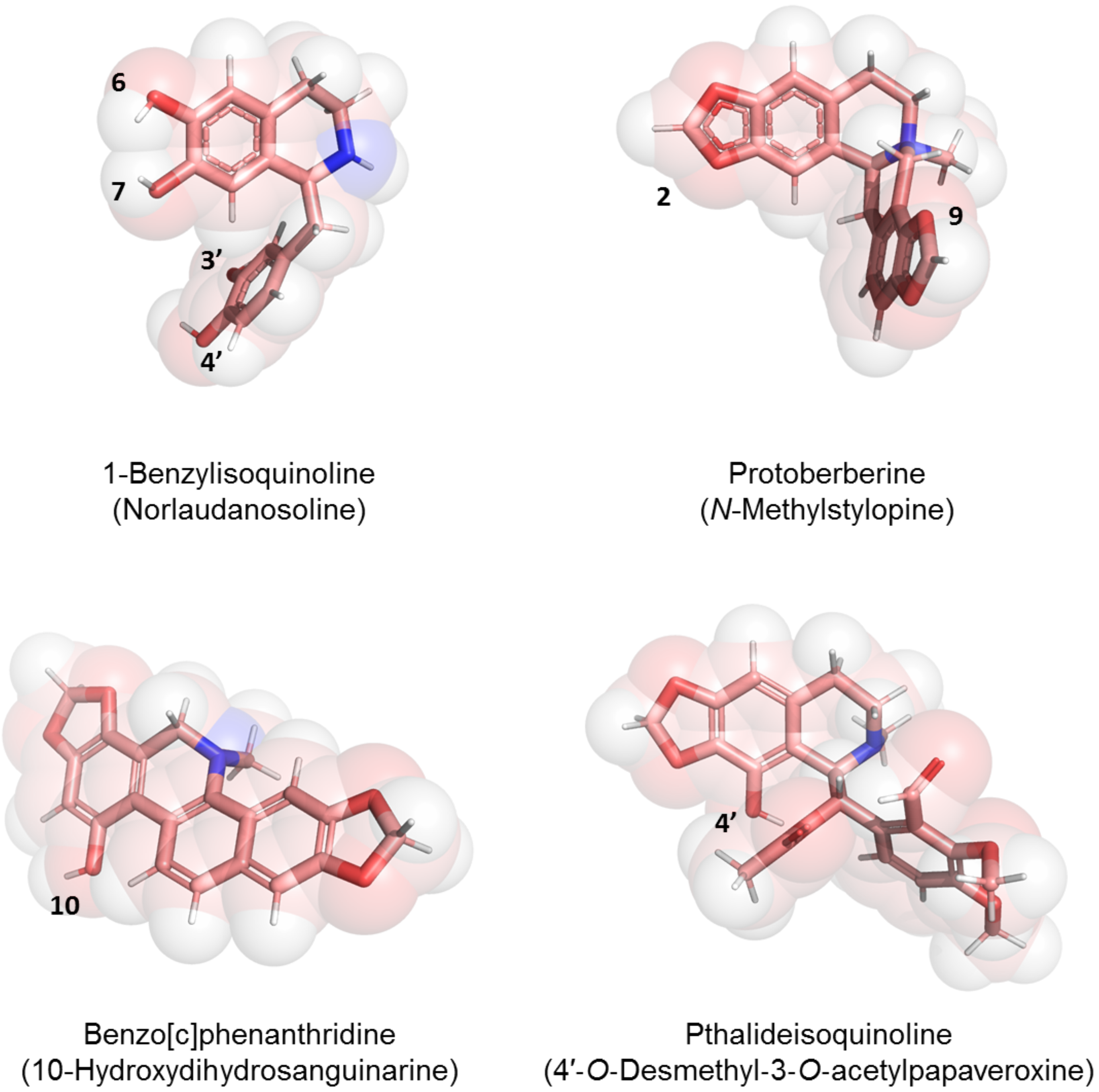


**Supplementary Figure 5. Three dimensional structures of BIAs.** Positions of hydroxyl groups targeted by BIA OMTs are labelled with atom numbers corresponding to Figure 2 and Supplementary Figure 4. Where possible, experimentally determined structures are shown. Norlaudanosoline was extracted from the Tf6OMT crystal structure (PDB 5ICE). (*S*)-*cis*-*N*-methylstylopine was extracted from the GfTNMT crystal structure (PDB 63PO). 10-Hydroxydihydrosanguinarine and 4′-*O*-desmethyl-3-*O*-acetylpapaveroxine were downloaded from PubChem.
