# Supplementary material for: Molecular Origins of Functional Diversity in Benzylisoquinoline Alkaloid Methyltransferases
Source: Front Plant Sci. 2019 Aug 30;10:1058. doi: 10.3389/fpls.2019.01058 (PMC6730481; doi:10.3389/fpls.2019.01058)
Supplement: Supplementary file 8 [file Image_6.pdf]

```
               |~~~~~~~~~~~~~~~~~~~~~~~~~~N-TERMINAL EXTENSION~~~~~~~~~~~~~~~~~~~~~~~~~|~~70s LOOP GATE~~~|~~~~~~~~~~~~~~~~~~~~~~~~~~~~~
                             HHHHHHHHHHHHHHHH    HHHHHHHHHHHHHHHHHHHH    HHHHHHHHHHHHH           HHHH     HHHHHH
               1                  20                  40                  60                  80                  100
CjCNMT         -----MAVEAK---QTKKAAIVELLKQLELGLVPYDDIKQLIRRELARRLQWGYKPTYEEQIAEIQNLTHSLRQMKIATEVET--LDSQLYEIPIEFLKIMNGSNLKGSCCYFKEDS-TT
TfCNMT         -----MAVEGKQV-APKKAIIVELLKKLELGLVPDDEIKKLIRIQLGRRLQWGCKSTYEEQIAQLVNLTHSLRQMKIATEVET--LDDQMYEVPIDFLKIMNGSNLKGSCCYFKNDS-TT
GFLNMT6        MGVEITESSSTTM-ANKKAEVGELLRKLEHGLLPYDEIRRLMRIELGRRLQWGYKPTHEQQLSQVLKLARSLRTMNIATEIDT--LDEQMYEVPIPFLQLMFGSTIKGSCCYFKDDS-TT
GFLNMT5        MG-----SNETNGELKTKEMVPDLLKRLESGLVADEELRKLIRFELERRLKWGYKPTHEQQLAELLKLAHSTKQMKIATETDS--LNSTMYEVPIPFLQLQFGSAIKESCCYFKDES-TT
GFLNMT1        MD-----LMATSKQ---VKKKEELLKNMELGLVPDEEIRRLIRIELEKRLKWGYKPTHQQQLAQLLDLVHSLKKMKIATEMES--LDLKLYEAPFSFVQIKHGSTIKESSSYFKDES-MT
PsCNMT         ------------MQ---LKAKEELLRNMELGLIPDQEIRQLIRVELEKRLQWGYKETHEEQLSQLLDLVHSLKGMKMATEMEN--LDLKLYEAPMEFLKIQHGSNMKQSAGYYTDES-TT
TfPavNMT       ----------METKQTKKEAVANLIKRIEHGEVSDEEIRGMMKIQVQKRLKWGYKPTHEQQLAQLVTFAQSLKGMEMAEEVDT--LDAELYEIPLPFLHIMCGKTLKFSPGYFKDES-TT
PsRNMT         --------MSTTMETTKISQQDDLWKNMELGQISDEEVRRLMKIGIEKRIKWGTKPTQQEQLAQLLDFNKSLRGMKMATEIDTLE-NHKIYETPESFNQIIGG---KESAGLFTDETTTT
GFLNMT4        --------MTMEANNAKKEAIENLWEQMMMGLVPDHEITRLMKSELQKRLNWGYKPTHQQQISQLLDFAKSLRRMEMSLDFDNLELDTKMYETPESFQLIMSGTTLKESSGLFTDET-AT
GfTNMT         MG--------SNEAQVKKESIGEIMGKLMQGEIGDEELSKRIKEIFGKRLQWGYKPTHQQQLAFNLDFIKSLKEMDMSGEIDT--MNEETYELPSAFLEAAFGKTIKQSGCYFKDET-TT
PsTNMT         MG--------S-IDEVKKESAGETLGRLLKGEIKDEELKKLIKFQFEKRLQWGYKSSHQEQLSFNLDFIKSLKKMEMSGEIET--MNKETYELPSEFLEAVFGKTVKQSMCYFTHES-AT
PbTNMT         MG--------S-IDEVKKESAGETLGRLLKGEIKDEELKKLIKFQFEKRLQWGYKSSHQEQLSFNLDFIKSLKKMEMSGEIET--MNKETYELPSEFLEAVFGKTVKQSMCYFKHES-AT
EcTNMT         ----------------MGSSAGEIMGRLMKGEIEDEELKKLIRHQWDRRIEWGYKPTHEKQLAFNLDFIKGLKEMVMSGEIDT--MNKETYELPTAFLEAVFGKTVKQSCCYFKDEN-ST
GFLNMT3        MD-------SKEVGIMKKESAEEILGRVMKGQIEDEELRELIKFQFERLLQWGYKPTLQQQLAFNLDFLKSLKELEMSGETEA--MNKETYELPTAFLEAIFGKTIKISACYFKDEL-MT
PsNMT4         -------------MDSKDQHGTKILERLVKGEIGDEELKELIRIRFEKRLQCGYKPTPQDQLASEMAFIKSLKDMKMSGELEA--VNTELYELPTAAVAATLGSTLKQSACYFKEES-MS
                                                             x                                             xx                x

                                  Motif I                 Motif II            Motif III         Motif IV
               ~~~~~SAM BINDING~~~~~~~~~~~~~~~~~~~~~~~~~~~~~~~~~~~~~~~~~~~~~~~~~~~~~~~~~~~~~~~~~~~~~~~~~~~~~~~~~~~~|~~~~~~~~~~~~~~~~~~~~~~~
                HHHHHHHHHHHHH          EEEEE     HHHHHHHH      EEEEE   HHHHHHHHHHH         EEEEE            EEEEEEE  HHH    HHHHHHHHH  EEEEE
                     120                  140                 160                 180                 200                 220
CjCNMT         LDEAEIAMLDLYCERAQIQDGQSVLDLGCGQGALTLHVAQKYKNCRVTAVTNSVSQKEYIEEESRRRNLLNVEVKLADITTH--EMAETYDRILVIELFEHMKNYELLLRKISEWISK-D
TfCNMT         LDEAEIAMLELYCERAQIKDGHSVLDLGCGQGALTLYVAQKYKNSRVTAVTNSVSQKEFIEEESRKRNLSNVEVLLADITTH--KMPDTYDRILVVELFEHMKNYELLLRKIKEWMAK-D
GFLNMT6        LDEAEIAMLDLYCERAQIKDGQSVLDLGCGQGALTLHVANKYKNCRVTAVTNSVSQKEFIEEQSRRLNLKNVEVMLADITTH--EMEDTYDRILVIELFEHMKNYELLLRKISKWLSK-D
GFLNMT5        LDEAEVAMMDLYLERTQIKDGQSILDLGCGLGALAFHIVQKYPNCNVLAITNSVEQKEFIEEKCKIRKVENVKVSLADICTL--EMKTTFDRIFAIGLLEHMKNYQLLLKKFSNWMKQ-D
GFLNMT1        LDEAEIAMLDLYVERAQIEDGQSVLDLGCGLGAVTLHVAKKYKNCHVTGLTNSVEQKDFIEGKCKELNLSNVKVILADVTSH--EMEDKFDRIFAVELIEHMKNYELLLRRISKWMKD-D
PsCNMT         LDEAEIAMLDLYMERAQIKDGQSVLDLGCGLGAVALFGANKFKKCQFTGVTSSVEQKDYIEGKCKELKLTNVKVLLADITTY--ETEERFDRIFAVELIEHMKNYQLLLKKISEWMKD-D
TfPavNMT       LDESEVYMMDLYCERAQIKDGQSILDLGCGHGSLTLHVAQKYRGCKVTGITNSVSQKEFIMDQCKKLDLSNVEIILEDVTKF--ETEITYDRIFAVALIEHMKNYELFLKKVSTWIAQ-Y
PsRNMT         MEEANTKMMDLYCERAGLKDGHTILDLGCGAGLLVLHLAKKYKKSKITGITNTSSHKEYILKQCKNLNLSNVEIILADVTKV--DIESTFDRVFVIGLIEHMKNFELFLRKISKWMKD-D
GFLNMT4        LDQTQIRMMDLYLEKAKIKDGQSILDLGCGHGALILHVAQKYRNCNVTGVTNSIAQKEFIFKQCKKLGLSNVEMVLADVTKC--EMKATFDHIFVIGLIEHMKNFELFLRKVSEWMKS-D
GfTNMT         IDEAEEASHELYCERAQIKDGQTVLDIGCGQGGLVLHIAQKYKNCHVTGLTNSKAQKNYILMQAEKLQLSNVDVILADVTKH--ESDKTYDRILVIETIEHMKNIQLFMKKLSTWMTE-D
PsTNMT         IDEAEEAAHELYCERAQIKDGQTVLDIGCGQGGLVLYIAQKYKNCHVTGLTNSKAQVNYLLKQAEKLGLTNVDAILADVTQY--ESDKTYDRLLMIEAIEHMKNLQLFMKKLSTWMTK-E
PbTNMT         IDEAEEAAHELYCERAQIKDGQTVLDIGCGQGGLVLYIARKYKKCHVTGLTNSKAQVNYLLKQAEKLGLTNVDAILADVTQY--ESDKTYDRLLMIEAIEHMKNLQLFMKKLSTWMTE-E
EcTNMT         IDEAEEAAHELYCERAQIKDGQTVLDIGCGQGGLVLYIAEKYKNCHVTGLTNSKAQANYIEQQAEKLELTNVDVIFADVTKF--DTDKTYDRILVVETIEHMKNIQLFMKKLSTWMTE-D
GFLNMT3        LDEAEEACHELYCERAQIRDGQTILDIGCGQGSLILHIAQKYKNCHITGITNSNGQKNYITTQAEKLQLSNVDVILADVTKF--DMEKTFDRVSVIGTIEHMKNMALFMKKVSSWMKE-D
PsNMT4         IDEAEIAAYELDCERAQIKDGQTILDIGCGFGGLVLHIAQKYKNSHVTGLTNSAEQRNYIMLQVEKLSLSNVDVILADVTKHEFENEKKFDRILVVEAIEHMKNIQLFLKKISNWMKDED
                                                                                                                   x  xx

               ~~~~~~~~~~~~~~~~~~~~~~~~~~~~~~~~~~~~~~~~~~~~~BIA BINDING~~~~~~~~~~~~~~~~~~~~~~~~~~~~~~~~~~~~~~~~~~~~~~~~~~~~~~~~~~~~~~~
               EEEEEEEEEE    EE                    EE E  HHHH     EEEEEEEE HHHHHHHHHHHHHHHHH  HHHHHHHHHH   HHHHHHHHHHHHHHHHHHHHHHHHHHHHH
                       240                 260                 280                 300                 320                 340
CjCNMT         GLLFLEHICHKTFAYHYEPLDDDDWFTEYVFPAGTMIIPSASFFLYFQDDVSVVNHWTLSGKHFSRTNEEWLKRLDANLDVIKPMFETLMGNE-EEAVKLINYWRGFCLSGMEMFGYNNG
TfCNMT         GLLFVEHICHKTFAYHYEPIDEDDWFTEYVFPAGTMIIPSASFFLYFQDDVSVVNHWTLSGKHFSRTNEEWLKRLDANVELIKPMFVTITGQCRQEAMKLINYWRGFCLSGMEMFGYNNG
GFLNMT6        GFLFIEHICHKTFAYHYEPIDEDDWYTEYIFPAGTMIIPSASFFLYFQDDLTVAKQWTLSGKHFARTKEEWLKRLNANVDEVMKIMESFSGS-KEGAVKWTNYWRGFCLSGMEMYGYNNG
GFLNMT5        GLLFIEHLCHKTLAYHYEPLDEDDWYTEYFFPAGTLTIISSSFLLYFQDDVSIVNHWSLSGKHFSRSNEEWLKRMDMKIDEVKEILEAAFENKDHDITKLINHWRFLAINATEMFGYNNG
GFLNMT1        GLLFIEHVCHKTFAYHYEPIDEDDWYTEYIFPAGTLTLSSASLLLYFQDDVSVVNHWTLSGKHYSRSHEEWLKRIDGNMDAVKEIMKSITK-TEEEAVKLINFWRIFCMCGAELFGYKDG
PsCNMT         GLLFVEHVCHKTLAYHYEPVDAEDWYTNYIFPAGTLTLSSASMLLYFQDDVSVVNQWTLSGKHYSRSHEEWLKNMDKNIVEFKEIMRSITK-TEKEAIKLLNFWRIFCMCGAELFGYKNG
TfPavNMT       GLLFVEHHCHKVFAYQYEPLDEDDWYTEYIFPSGTLVMSSSSILLYFQEDVSVVNHWTLSGKHPSLGFKQWLKRLDDNIDEVKEIFESFYGS-KEKAMKFITYWRVFCIAHSQMYSTNNG
PsRNMT         GLLLLEHLCHKSFSDHWEPLSEDDWYAKNFFPSGTLVIPSATCLLYFQEDVTVIDHWILSGNNFARSNEVILKRIDGKIEEVKDIFMSFYGIGREEAVKLINWWRLLCITANELFKYNNG
GFLNMT4        GLLFMEHYCHKSFAYQWEPMDDDDLFSKYVFPPGSAIIPSASFLLYFQDDLTVVDHWTLSGNHFARTHQEWLKRIDSQSDEIKGIFESFYGISKEEAVKLINYWRVFCLFGVEMFGYNNG
GfTNMT         SLLFVDHICHKTFSHHFEAIDEDDWYSGFIFPKGCVTILSASALLYFQDDVTILDHWVVNGMHMARSVDAWRKKLDKNMELAREILLPGLG-SKEAVNGVITHIRTFCMGGYEQFSYNNG
PsTNMT         SLLFVDHVCHKTFAHFFEAVDEDDWYSGFIFPPGCATILAANSLLYFQDDVSVVDHWVVNGMHMARSVDIWRKALDKNMEAAKEILLPGLGGSHETVNGVVTHIRTFCMGGYEQFSMNNG
PbTNMT         SLLFVDHVCHKTFAHFFEAVDEDDWYSGFIFPPGCATILAANSLLYFQDDVSVVDHWVVNGMHMARSVDIWRKALDKNMEAAKEILLPGLGGSHEAVNGVVTHIRTFCMGGYEQFSMNDG
EcTNMT         SLLFVDHISHKTFNHNFEALDEDDWYSGFIFPKGCVTILSSSTLLYFQDDVSALDHWVVNGMHMARSVEAWRKKLDETIEAAREILEPGLG-SKEAVNQVITHIRTFCIGGYEQFSYNNG
GFLNMT3        GLLFVDHVCNKTFSHHYEALDEDDWYSGYKFPKGSVTILWANALLYFQDDVSVVDHWLLNGMHMARTQVEWGKKLNKNIEAVKEILEAGLG-SKEAANQVITHMRTCGIGGYEQFSYNNG
PsNMT4         SFLFVVHLCHKAFSQHFEALDEDDWYTSYVLPEGSVTYLSASALLYFQDDVSVVDQWLLSGTHMARSQEEWFKRFKINLEAASKALTVGLG-SEEAANQVNIQYKTFFMGYVVQFSFNNG
                                x                                                  x  x                                  xxxxx

               ~~~~~~~~~~~~~~~~~~|
                 EEEEEEEEE

CjCNMT         EEWMASHVLFKKK------      358
TfCNMT         EEWMASHVLFKKK------      361
GFLNMT6        EEWMACHVLFKK-------      364
GFLNMT5        EEWMVSQVLFKKK------      362
GFLNMT1        EEWMMSHVLFKKKQLLQQC      364
PsCNMT         EEWMLTHLLFKKK------      351
TfPavNMT       EEWMLSQVLFKKK------      356
PsRNMT         EEWLISQLLFKKKLMTCI-      363
GFLNMT4        EEWMISHLLFKKK------      361
GfTNMT         EEWMVAQMLFKKK------      358
PsTNMT         DEWMVAQLLFKKK------      358
PbTNMT         DEWMVAQLLFKKK------      358
EcTNMT         EEWMITQILFKKK------      350
GFLNMT3        EEWMISHVLFKKK------      359
PsNMT4         EEWMISHYLFKKK------      356
```

Supplementary Figure 6. Amino acid sequence alignment of select BIA *N*-methyltransferases characterized *in vitro*. Protein sequences were obtained by translation of nucleotide sequences deposited in Genbank (accession numbers listed in Supplementary Table 1) and aligned using Clustal Omega under default parameters (Chojnacki et al., 2017). Major domains and key residues are adapted from structural studies of TfPavNMT and CjCNMT (Torres et al., 2016; Bennett et al., 2018). Residues shaded in *yellow* form hydrogen bonds (directly or water-mediated) with SAM/SAH. Residues shaded in *grey* form part of the dimerization interface. Residues shaded in *cyan* interact with the alkaloid substrate. Residues

(*continued on next page*)

shaded in *red* contribute to catalysis and/or hydrogen bond with the target nitrogen atom. Residues shaded in *purple* contribute to positioning the active site gate upon substrate binding. Secondary structure is given according to DSSP analysis of CjCNMT (Touw et al., 2015). “X” indicates residues subjected to mutational analysis. Percentage identities are provided in Supplementary Figure 2.

Bennett, M. R., Thompson, M. L., Shepherd, S. A., Dunstan, M. S., Herbert, A. J., Smith, D. R. M., et al. (2018). Structure and biocatalytic scope of coclaurine *N*-methyltransferase. *Angew. Chemie - Int. Ed.* 57, 10600–10604. doi:10.1002/anie.201805060.

Chojnacki, S., Cowley, A., Lee, J., Foix, A., and Lopez, R. (2017). Programmatic access to bioinformatics tools from EMBL-EBI update: 2017. *Nucleic Acids Res.* 45, W550–W553. doi:10.1093/nar/gkx273.

Torres, M. A., Hoffarth, E., Eugenio, L., Savtchouk, J., Chen, X., Morris, J. S., et al. (2016). Structural and functional studies of pavine *N*-methyltransferase from *Thalictrum flavum* reveal novel insights into substrate recognition and catalytic mechanism. *J. Biol. Chem.* 291, 23403–23415. doi:10.1074/jbc.M116.747261.

Touw, W. G., Baakman, C., Black, J., te Beek, T. A. H., Krieger, E., Joosten, R. P., et al. (2015). A series of PDB-related databanks for everyday needs. *Nucleic Acids Res.* 43, D364–D368. doi:10.1093/nar/gku1028.
